# Supplementary material for: Sex-disaggregated data along the gendered health pathways: A review and analysis of global data on hypertension, diabetes, HIV, and AIDS
Source: PLoS Med. 2025 May 1;22(5):e1004592. doi: 10.1371/journal.pmed.1004592 (PMC12045488; doi:10.1371/journal.pmed.1004592)
Supplement: S1 Text — Table A. List of countries with available data on risk factors, disease prevalence and death rate, and care cascade for hypertension, diabetes, and HIV and AIDS. Fig A. Sex differences (significant when non-overlapping confidence intervals of estimates between females and males) in health pathways of hypertension, by income group. Fig B. Sex differences (significant when non-overlapping confidence intervals of estimates between females and males) in health pathways of hypertension, by region. Fig C. Sex differences (significant when non-overlapping confidence intervals of estimates between females and males) in health pathways of diabetes, by income group. Diabetes care cascade data are available only for age groups 30–44, 45–59, and 60–79. Fig D. Sex differences (significant when non-overlapping confidence intervals of estimates between females and males) in health pathways of diabetes, by region. Diabetes care cascade data are available only for age groups 30–44, 45–59, and 60–79. Fig E. Sex differences (significant when non-overlapping confidence intervals of estimates between females and males) in health pathways of HIV and AIDS, by income group. HIV and AIDS care cascade data are available only for the age group 15+ . Fig F. Sex differences (significant when non-overlapping confidence intervals of estimates between females and males) in health pathways of HIV and AIDS, by region. HIV and AIDS care cascade data are available only for the age group 15+ . Fig G. Percentages of countries with significant sex differences in global health pathways of hypertension, diabetes, and HIV and AIDS (significant when non-overlapping confidence intervals of estimates between females and males). Fig H. Percentages of countries with significant sex differences in health pathways of hypertension, by income group (significant when non-overlapping confidence intervals of estimates between females and males). Fig I. Percentages of countries with significant sex differences in health [file pmed.1004592.s001.docx]

**Supporting Information**

**Table of contents**

**Table A.** List of countries with available data on risk factors, disease prevalence and death rate, and care cascade

for hypertension, diabetes, and HIV and AIDS.

**Fig A.** Sex differences (significant when non-overlapping confidence intervals of estimates between females and males) in health pathways of hypertension, by income group.

**Fig B.** Sex differences (significant when non-overlapping confidence intervals of estimates between females and males) in health pathways of hypertension, by region.

**Fig C.** Sex differences (significant when non-overlapping confidence intervals of estimates between females and males) in health pathways of diabetes, by income group. Diabetes care cascade data are available only for age groups 30-44, 45-59, and 60-79.

**Fig D.** Sex differences (significant when non-overlapping confidence intervals of estimates between females and males) in health pathways of diabetes, by region. Diabetes care cascade data are available only for age groups 30-44, 45-59, and 60-79.

**Fig E.** Sex differences (significant when non-overlapping confidence intervals of estimates between females and males) in health pathways of HIV and AIDS, by income group. HIV and AIDS care cascade data are available only for age group 15+.

**Fig F.** Sex differences (significant when non-overlapping confidence intervals of estimates between females and males) in health pathways of HIV and AIDS, by region. HIV and AIDS care cascade data are available only for age group 15+.

Fig G. Percentages of countries with significant sex differences in global health pathways of hypertension, diabetes, and HIV and AIDS (significant when non-overlapping confidence intervals of estimates between females and males).

Fig H. Percentages of countries with significant sex differences in health pathways of hypertension, by income group (significant when non-overlapping confidence intervals of estimates between females and males).

Fig I. Percentages of countries with significant sex differences in health pathways of hypertension, by region (significant when non-overlapping confidence intervals of estimates between females and males).

Fig J. Percentages of countries with significant sex differences in health pathways of diabetes, by income group (significant when non-overlapping confidence intervals of estimates between females and males).

Fig K. Percentages of countries with significant sex differences in health pathways of diabetes, by region (significant when non-overlapping confidence intervals of estimates between females and males).

Fig L. Percentages of countries with significant sex differences in health pathways of HIV and AIDS, by income group (significant when non-overlapping confidence intervals of estimates between females and males).

Fig M. Percentages of countries with significant sex differences in health pathways of HIV and AIDS, by region (significant when non-overlapping confidence intervals of estimates between females and males).

**Table A.** List of countries with available data on risk factors, disease prevalence and death rate, and care cascade for hypertension, diabetes, and HIV and AIDS.

| **Risk Factors, Disease Prevalence, and Death Rates**  **(204 countries)** | **Hypertension  Care Cascade**  **(200 countries)** | **Diabetes  Care Cascade (39 countries)** | **HIV and AIDS**  **Care Cascade**  **(76 countries)** |
| --- | --- | --- | --- |
| Afghanistan, Albania, Algeria, American Samoa, Andorra, Angola, Antigua and Barbuda, Argentina, Armenia, Australia, Austria, Azerbaijan, Bahamas, Bahrain, Bangladesh, Barbados, Belarus, Belgium, Belize, Benin, Bermuda, Bhutan, Bolivia, Bosnia and Herzegovina, Botswana, Brazil, Brunei Darussalam, Bulgaria, Burkina Faso, Burundi, Cape Verde, Cambodia, Cameroon, Canada, Central African Republic, Chad, Chile, China, Colombia, Comoros, Republic of Congo, Cook Islands, Costa Rica, Ivory Coast, Croatia, Cuba, Cyprus, Czech Republic, Denmark, Djibouti, Dominica, Dominican Republic, Democratic Republic of the Congo, Ecuador, Egypt, El Salvador, Equatorial Guinea, Eritrea, Estonia, Ethiopia, Fiji, Finland, France, Gabon, Gambia, Georgia, Germany, Ghana, Greece, Greenland, Grenada, Guam, Guatemala, Guinea, Guinea-Bissau, French Guiana, Haiti, Honduras, Hungary, Iceland, India, Indonesia, Iran, Iraq, Ireland, Israel, Italy, Jamaica, Japan, Jordan, Kazakhstan, Kenya, Kiribati, Kuwait, Kyrgyzstan, Laos, Latvia, Lebanon, Lesotho, Liberia, Libya, Lithuania, Luxembourg, Macedonia, Madagascar, Malawi, Malaysia, Maldives, Mali, Malta, Marshall Islands, Mauritania, Mauritius, Mexico, Micronesia, Moldova, Monaco, Mongolia, Montenegro, Morocco, Mozambique, Myanmar, Namibia, Nauru, Nepal, Netherlands, New Zealand, Nicaragua, Niger, Nigeria, Niue, North Korea, Northern Mariana Islands, Norway, Occupied Palestinian Territory, Oman, Pakistan, Palau, Panama, Papua New Guinea, Paraguay, Peru, Philippines, Poland, Portugal, Puerto Rico, Qatar, Romania, Russia, Rwanda, Saint Kitts and Nevis, Saint Lucia, Saint Vincent and the Grenadines, Samoa, San Marino, Sao Tome and Principe, Saudi Arabia, Senegal, Serbia, Seychelles, Sierra Leone, Singapore, Slovakia, Slovenia, Solomon Islands, Somalia, South Africa, South Korea, South Sudan, Spain, Sri Lanka, Sudan, Suriname, Swaziland, Sweden, Switzerland, Syria, Taiwan, Tajikistan, Tanzania, Thailand, Timor-Leste, Togo, Tokelau, Tonga, Trinidad and Tobago, Tunisia, Turkey, Turkmenistan, Tuvalu, Uganda, Ukraine, United Arab Emirates, UK, USA, United States Virgin Islands, Uruguay, Uzbekistan, Vanuatu, Venezuela, Vietnam, Yemen, Zambia, Zimbabwe | Afghanistan, Albania, Algeria, American Samoa, Andorra, Angola, Antigua and Barbuda, Argentina, Armenia, Australia, Austria, Azerbaijan, Bahamas, Bahrain, Bangladesh, Barbados, Belarus, Belgium, Belize, Benin, Bermuda, Bhutan, Bolivia, Bosnia and Herzegovina, Botswana, Brazil, Brunei Darussalam, Bulgaria, Burkina Faso, Burundi, Cape Verde, Cambodia, Cameroon, Canada, Central African Republic, Chad, Chile, China, Colombia, Comoros, Republic of Congo, Cook Islands, Costa Rica, Ivory Coast, Croatia, Cuba, Cyprus, Czech Republic, Denmark, Djibouti, Dominica, Dominican Republic, Democratic Republic of the Congo, Ecuador, Egypt, El Salvador, Equatorial Guinea, Eritrea, Estonia, Ethiopia, Fiji, Finland, France, French Polynesia, Gabon, Gambia, Georgia, Germany, Ghana, Greece, Greenland, Grenada, Guatemala, Guinea, Guinea-Bissau, French Guiana, Haiti, Honduras, Hungary, Iceland, India, Indonesia, Iran, Iraq, Ireland, Israel, Italy, Jamaica, Japan, Jordan, Kazakhstan, Kenya, Kiribati, Kuwait, Kyrgyzstan, Laos, Latvia, Lebanon, Lesotho, Liberia, Libya, Lithuania, Luxembourg, Macedonia, Madagascar, Malawi, Malaysia, Maldives, Mali, Malta, Marshall Islands, Mauritania, Mauritius, Mexico, Micronesia, Moldova, Mongolia, Montenegro, Morocco, Mozambique, Myanmar, Namibia, Nauru, Nepal, Netherlands, New Zealand, Nicaragua, Niger, Nigeria, Niue, North Korea, Norway, Occupied Palestinian Territory, Oman, Pakistan, Palau, Panama, Papua New Guinea, Paraguay, Peru, Philippines, Poland, Portugal, Puerto Rico, Qatar, Romania, Russia, Rwanda, Saint Kitts and Nevis, Saint Lucia, Saint Vincent and the Grenadines, Samoa, Sao Tome and Principe, Saudi Arabia, Senegal, Serbia, Seychelles, Sierra Leone, Singapore, Slovakia, Slovenia, Solomon Islands, Somalia, South Africa, South Korea, South Sudan, Spain, Sri Lanka, Sudan, Suriname, Swaziland, Sweden, Switzerland, Syria, Taiwan, Tajikistan, Tanzania, Thailand, Timor-Leste, Togo, Tokelau, Tonga, Trinidad and Tobago, Tunisia, Turkey, Turkmenistan, Tuvalu, Uganda, Ukraine, United Arab Emirates, UK, USA, Uruguay, Uzbekistan, Vanuatu, Venezuela, Vietnam, Yemen, Zambia, Zimbabwe | Afghanistan,  Algeria,  Armenia,  Azerbaijan,  Bangladesh,  Belarus,  Benin,  Botswana,  Brunei,  Cape Verde,  Ecuador,  Swaziland,  Ethiopia,  Georgia,  Guyana,  Iraq,  Jordan,  Kenya,  Kiribati,  Kuwait,  Kyrgyzstan,  Lebanon,  Malawi,  Morocco,  Myanmar,  Nauru,  Nepal,  Republic of Moldova,  Sao Tome,  Solomon Islands,  Sri Lanka,  Sudan,  Tajikistan,  Timor Leste, Turkmenistan,  Tuvalu,  Uganda,  Vietnam,  Zambia | Albania, Algeria, Armenia, Australia, Azerbaijan, Barbados, Belarus, Belize, Bhutan, Botswana, Bulgaria, Cape Verde, Cambodia, Chile, Colombia, Comoros, Cuba, Czechia, Ivory Coast, Djibouti, Dominican Republic, Ecuador, El Salvador, Eritrea, Swaziland, Ethiopia, Fiji, Georgia, Ghana, Guatemala, Haiti, Honduras, Italy,, Kazakhstan, Kenya, Kuwait, Kyrgyzstan, Lao People's Democratic Republic, Lebanon, Lesotho, Lithuania, Luxembourg, Malawi, Mauritius, Mexico, Mongolia, Montenegro, Morocco, Mozambique, Myanmar, Nepal, Netherlands, Nicaragua, Niger, Nigeria, North Macedonia, Oman, Peru, Qatar, Republic of Moldova, Rwanda, Saudi Arabia, Senegal, Singapore, Slovenia, South Africa, Sri Lanka, Suriname, Switzerland, Tajikistan, Thailand, Uganda, Ukraine, United Republic of Tanzania, Uruguay  Zambia |

**Fig A.** Sex differences (significant when non-overlapping confidence intervals of estimates between females and males) in health pathways of hypertension, by income group.

| High-income countries* | Upper-middle-income countries* |
| --- | --- |
| 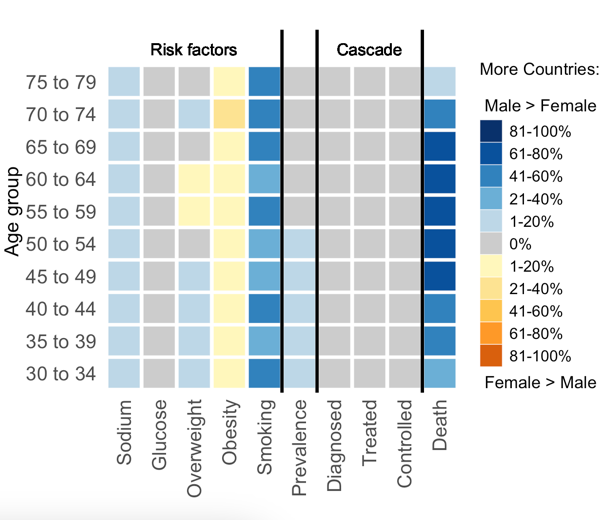  * Risk factors, prevalence, death: 67 countries.  Cascade: 63 countries. | 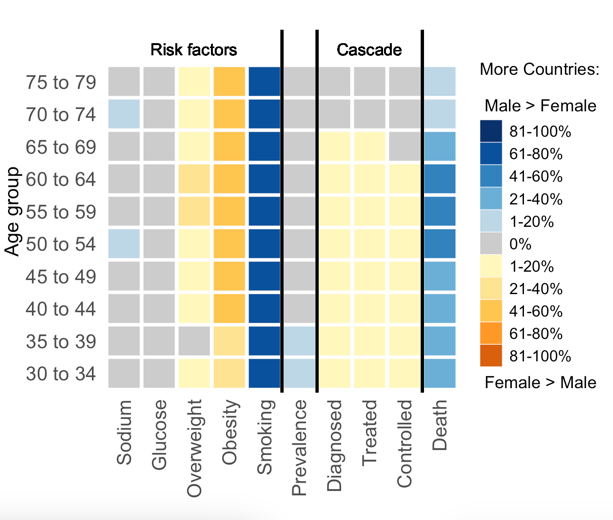  * Risk factors, prevalence, death: 54 countries. Cascade: 54 countries. |
| Lower-middle-income countries* | Low-income countries* |
| 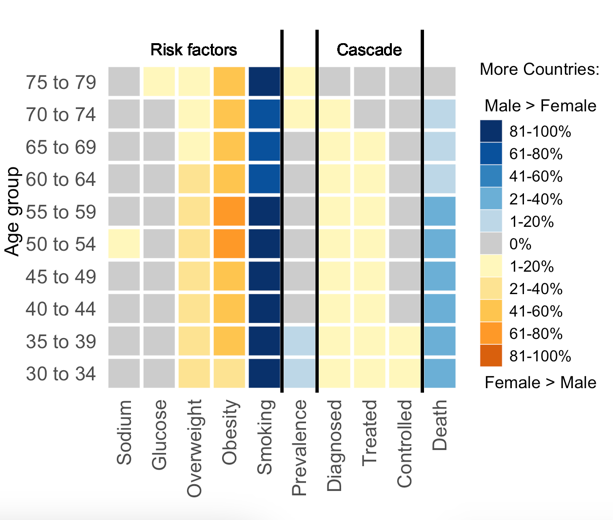  * Risk factors, prevalence, death: 54 countries. Cascade: 54 countries. | 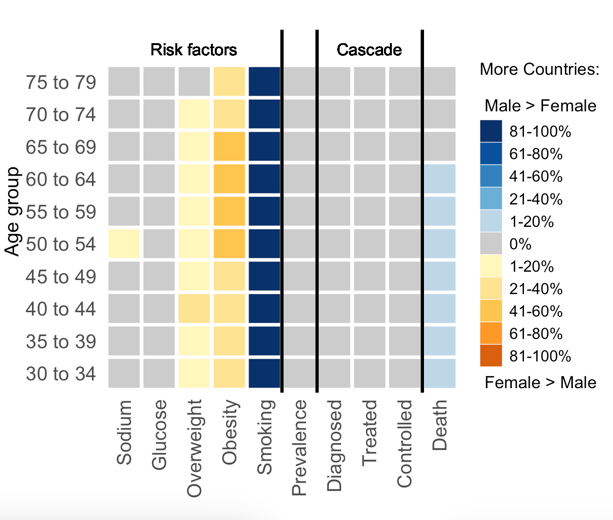  * Risk factors, prevalence, death: 26 countries. Cascade: 26 countries. |

**Fig B.** Sex differences (significant when non-overlapping confidence intervals of estimates between females and males) in health pathways of hypertension, by region.

| Europe and Central Asia* | South Asia* |
| --- | --- |
| 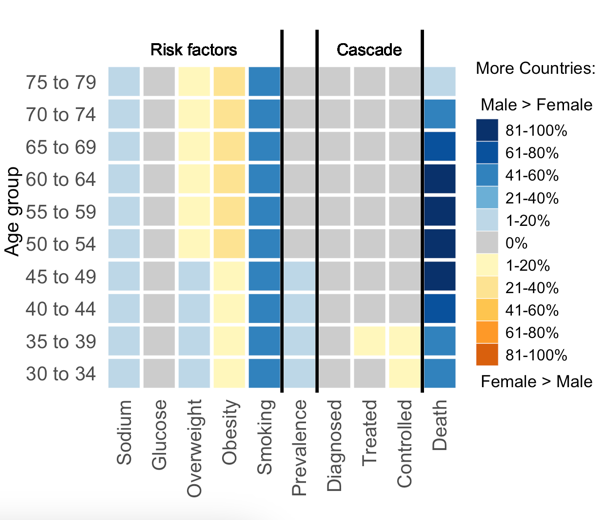  * Risk factors, prevalence, death: 52 countries. Cascade: 50 countries. | 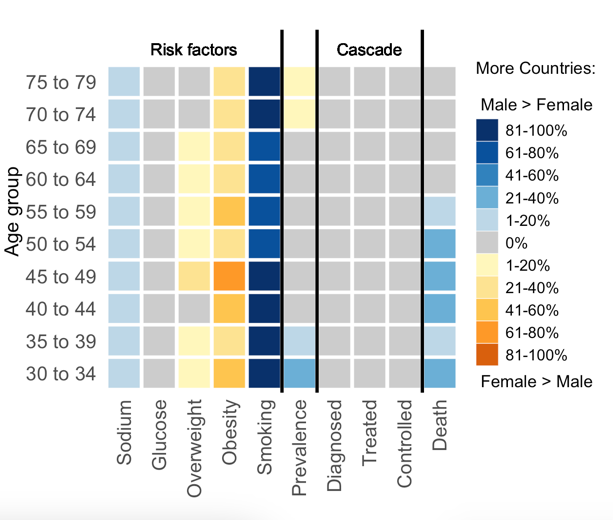  * Risk factors, prevalence, death: 8 countries. Cascade: 8 countries. |
| Sub-Saharan Africa* | Middle East and North Africa* |
| 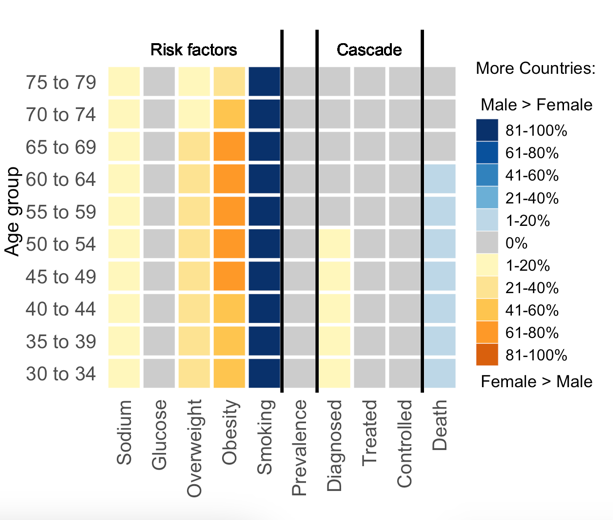  * Risk factors, prevalence, death: 44 countries. Cascade: 44 countries. | 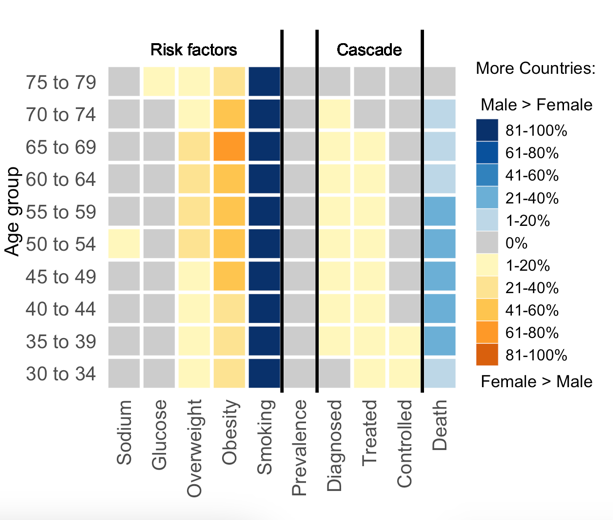  * Risk factors, prevalence, death: 22 countries. Cascade: 22 countries. |
| Latin America & the Caribbean* | East Asia and Pacific* |
| 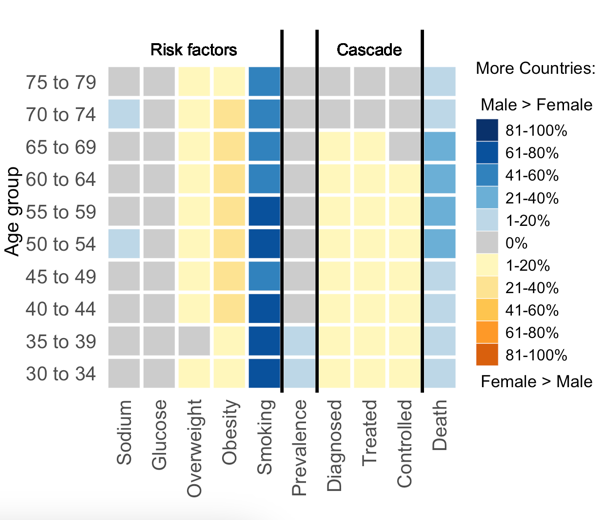  * Risk factors, prevalence, death: 38 countries. Cascade: 37 countries. | 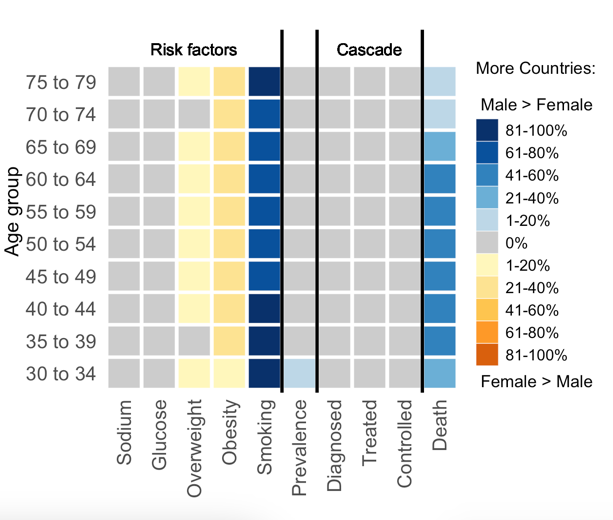  * Risk factors, prevalence, death: 34 countries. Cascade: 33 countries. |
| North America* |  |
| 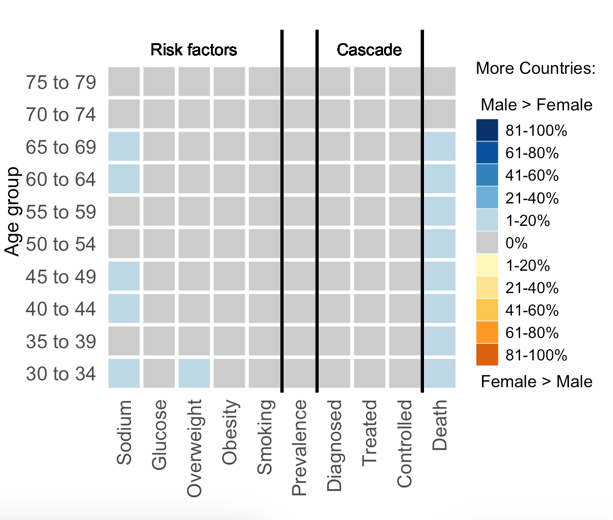  * Risk factors, prevalence, death: 3 countries. Cascade: 3 countries. |  |

**Fig C.** Sex differences (significant when non-overlapping confidence intervals of estimates between females and males) in health pathways of diabetes, by income group. Diabetes care cascade data are available only for age groups 30-44, 45-59, and 60-79.

| High-income countries* | Upper-middle-income countries* |
| --- | --- |
| 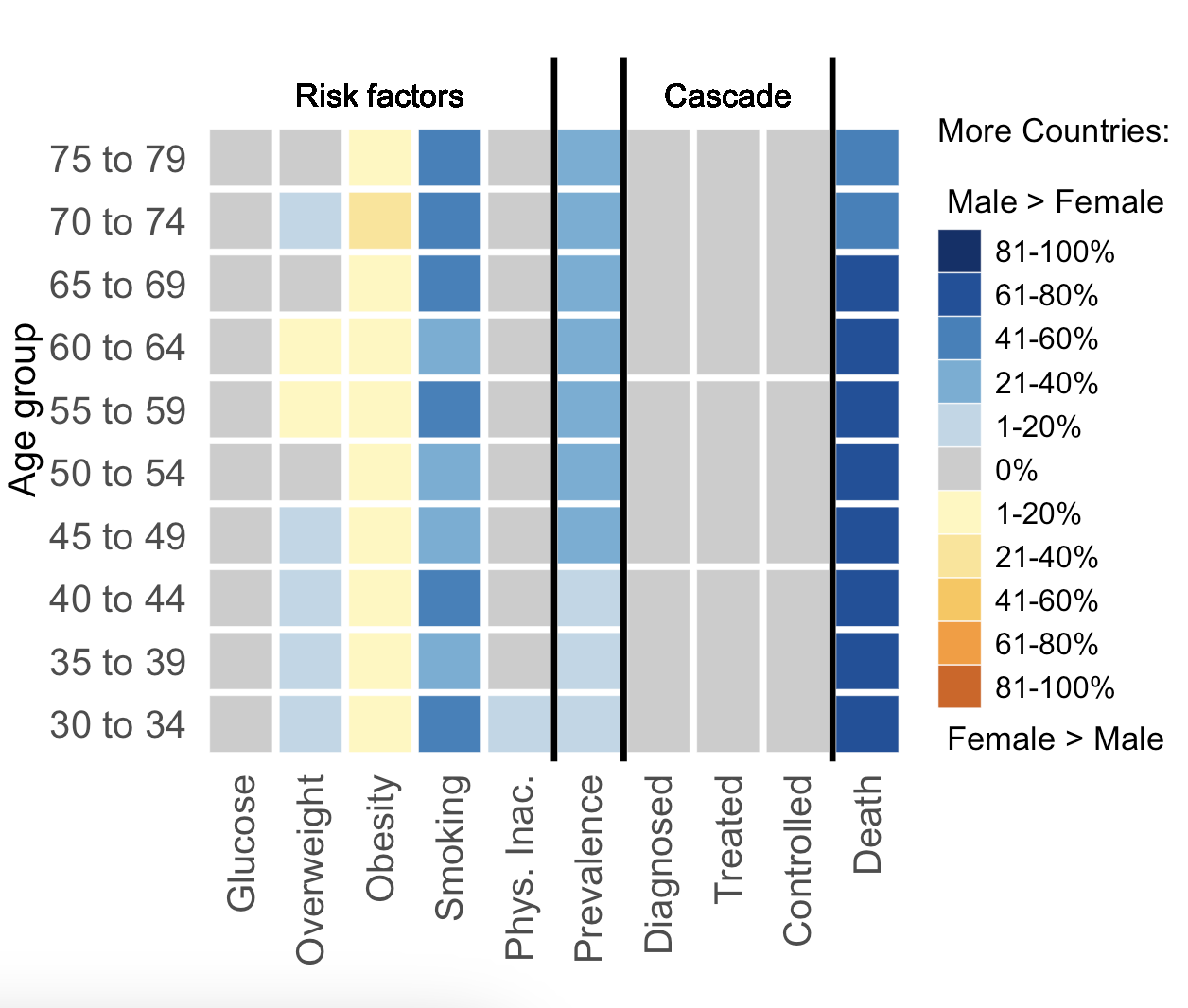  * Risk factors, prevalence, death: 67 countries.  Cascade: 4 countries.  Phys. Inac.: Physical Inactivity. | 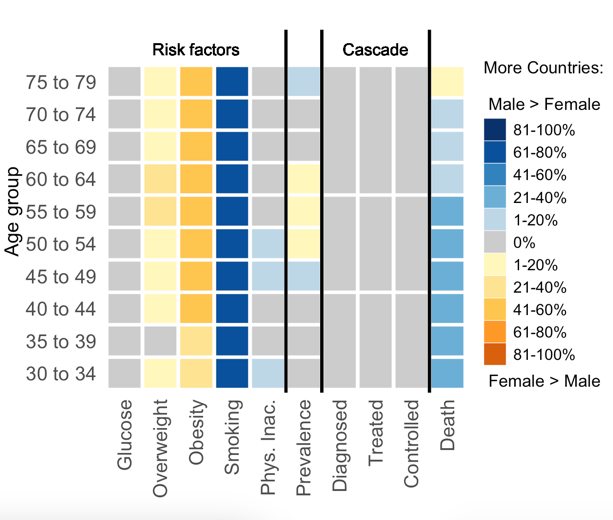  * Risk factors, prevalence, death: 54 countries. Cascade: 10 countries.  Phys. Inac.: Physical Inactivity. |
| Lower-middle-income countries* | Low-income countries* |
| 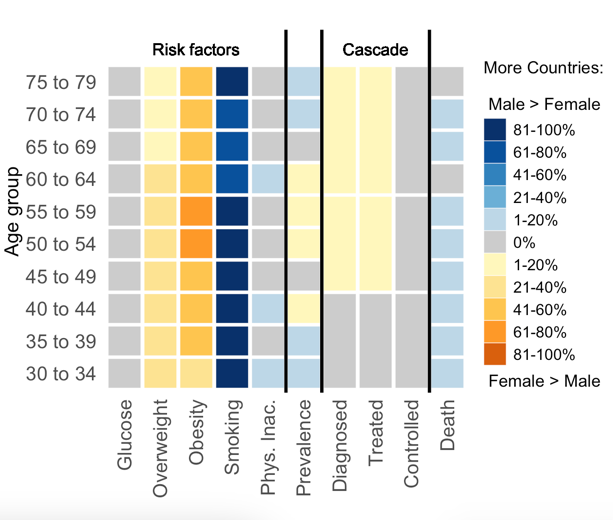  * Risk factors, prevalence, death: 54 countries. Cascade: 20 countries.  Phys. Inac.: Physical Inactivity. | 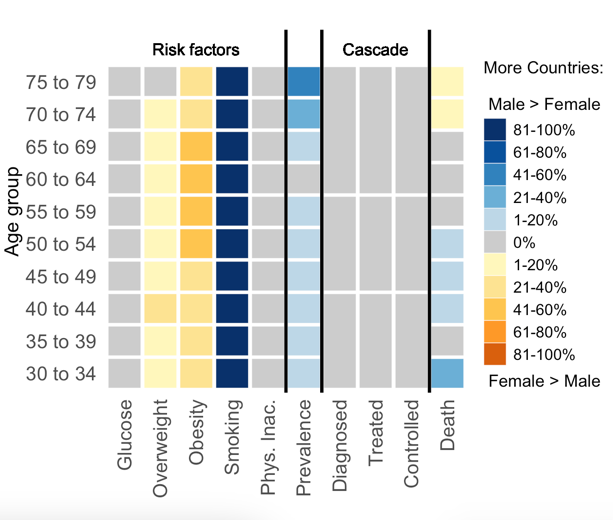  * Risk factors, prevalence, death: 26 countries. Cascade: 5 countries.  Phys. Inac.: Physical Inactivity. |

**Fig D.** Sex differences (significant when non-overlapping confidence intervals of estimates between females and males) in health pathways of diabetes, by region. Diabetes care cascade data are available only for age groups 30-44, 45-59, and 60-79.

| Europe and Central Asia* | South Asia* |
| --- | --- |
| 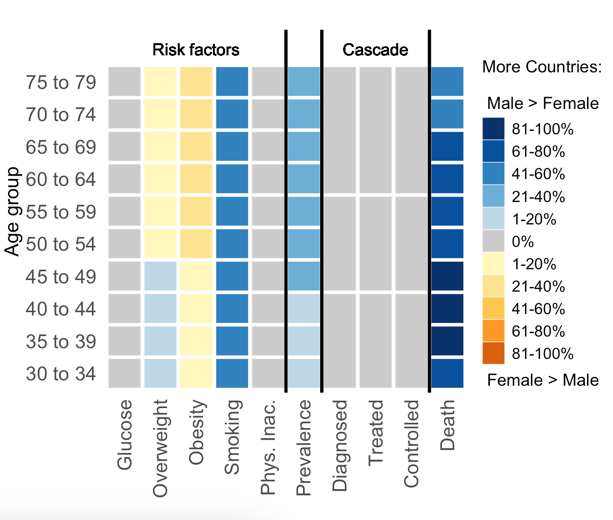  * Risk factors, prevalence, death: 52 countries. Cascade: 8 countries.  Phys. Inac.: Physical Inactivity. | 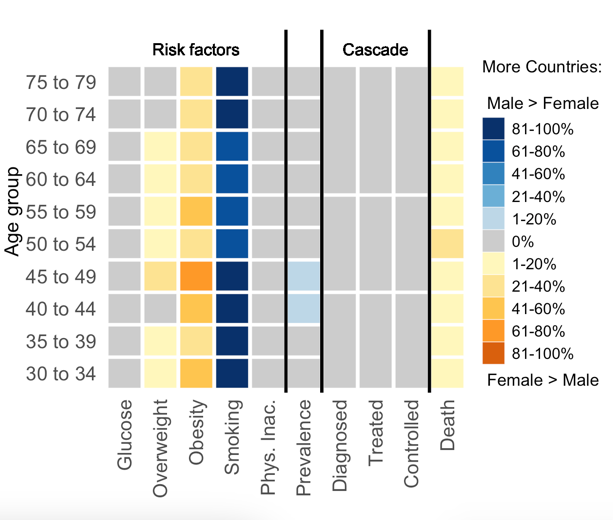  * Risk factors, prevalence, death: 8 countries. Cascade: 4 countries.  Phys. Inac.: Physical Inactivity. |
| Sub-Saharan Africa* | Middle East and North Africa* |
| 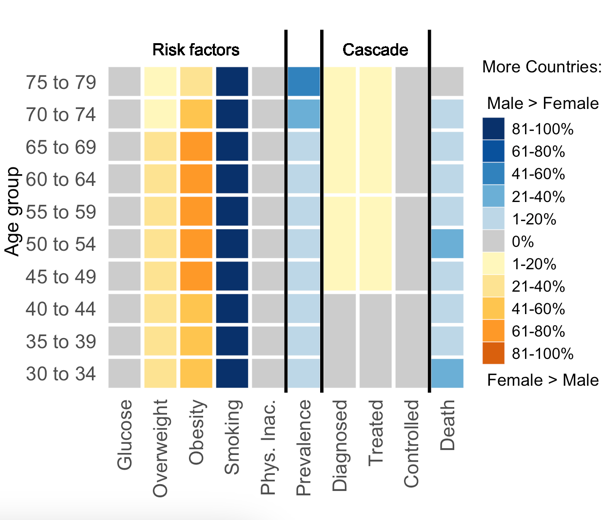  * Risk factors, prevalence, death: 44 countries. Cascade: 11 countries.  Phys. Inac.: Physical Inactivity. | 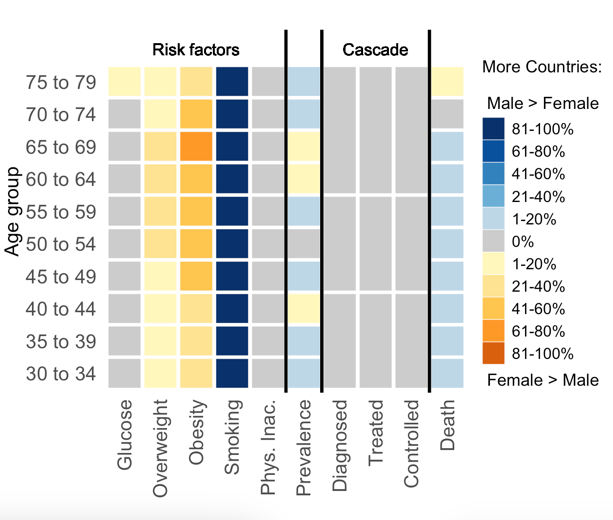  * Risk factors, prevalence, death: 22 countries. Cascade: 6 countries.  Phys. Inac.: Physical Inactivity. |
| Latin America & the Caribbean* | East Asia and Pacific* |
| 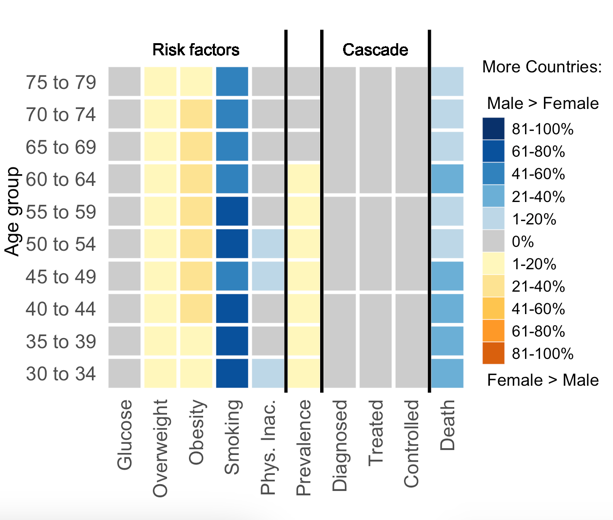  * Risk factors, prevalence, death: 38 countries. Cascade: 2 countries.  Phys. Inac.: Physical Inactivity. | 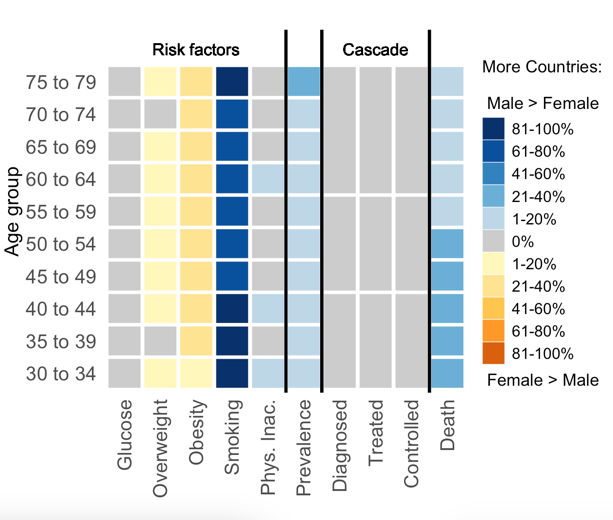  * Risk factors, prevalence, death: 34 countries. Cascade: 8 countries.  Phys. Inac.: Physical Inactivity. |
| North America* |  |
| 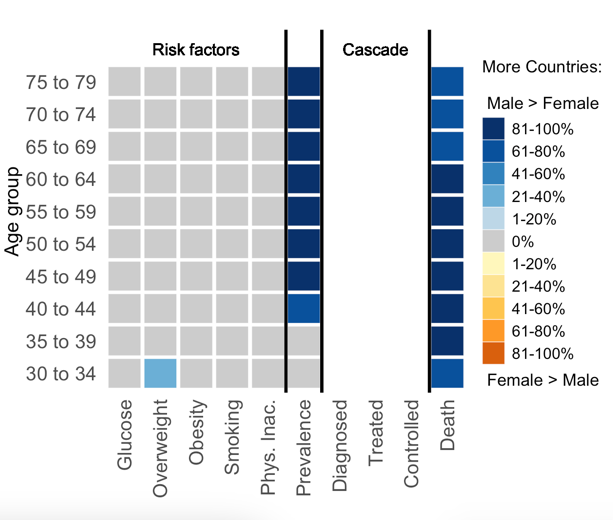  * Risk factors, prevalence, death: 3 countries. Cascade: No countries.  Phys. Inac.: Physical Inactivity. |  |

**Fig E.** Sex differences (significant when non-overlapping confidence intervals of estimates between females and males) in health pathways of HIV and AIDS, by income group. HIV and AIDS care cascade data are available only for age group 15+.

| High-income countries* | Upper-middle-income countries* |
| --- | --- |
| 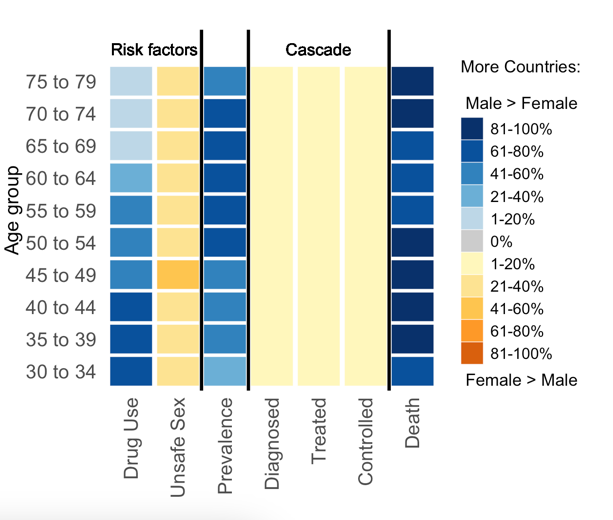  * Risk factors, prevalence, death: 67 countries.  Cascade: 16 countries. | 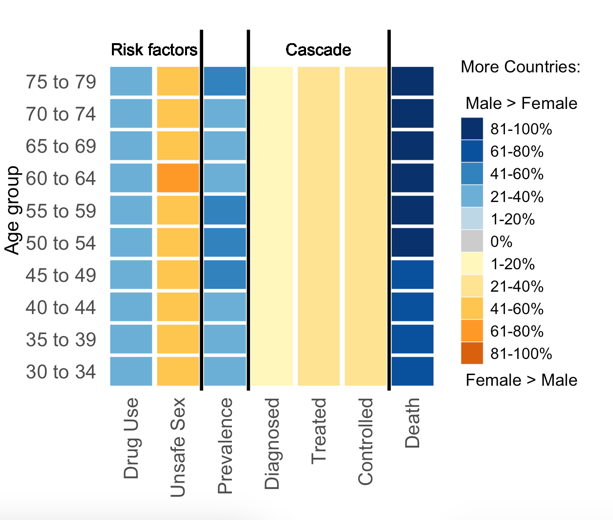  * Risk factors, prevalence, death: 54 countries. Cascade: 25 countries. |
| Lower-middle-income countries* | Low-income countries* |
| 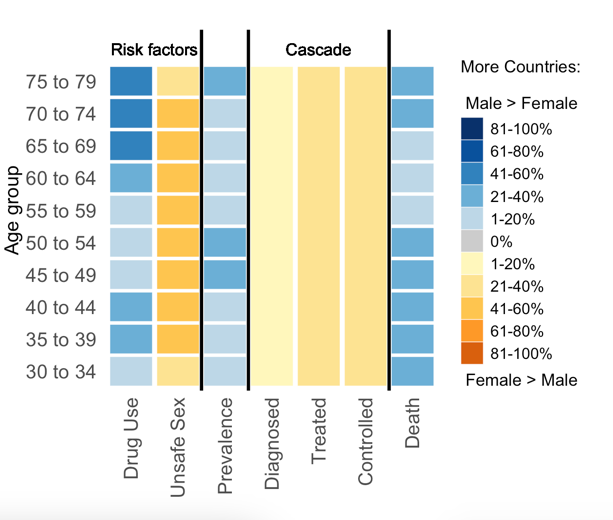  * Risk factors, prevalence, death: 54 countries. Cascade: 28 countries. | 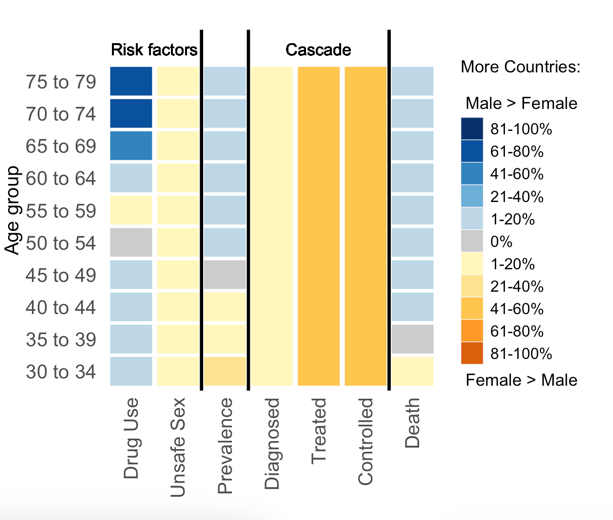  * Risk factors, prevalence, death: 26 countries. Cascade: 7 countries. |

**Fig F.** Sex differences (significant when non-overlapping confidence intervals of estimates between females and males) in health pathways of HIV and AIDS, by region. HIV and AIDS care cascade data are available only for age group 15+.

| Europe and Central Asia* | South Asia* |
| --- | --- |
| 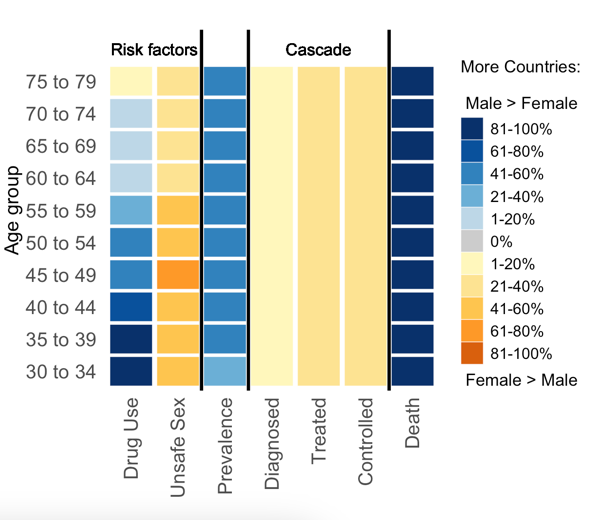  * Risk factors, prevalence, death: 52 countries. Cascade: 20 countries. | 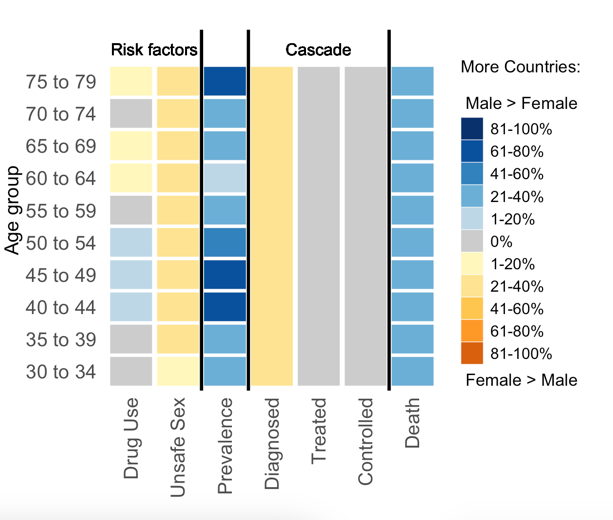  * Risk factors, prevalence, death: 8 countries. Cascade: 3 countries. |
| Sub-Saharan Africa* | Middle East and North Africa* |
| 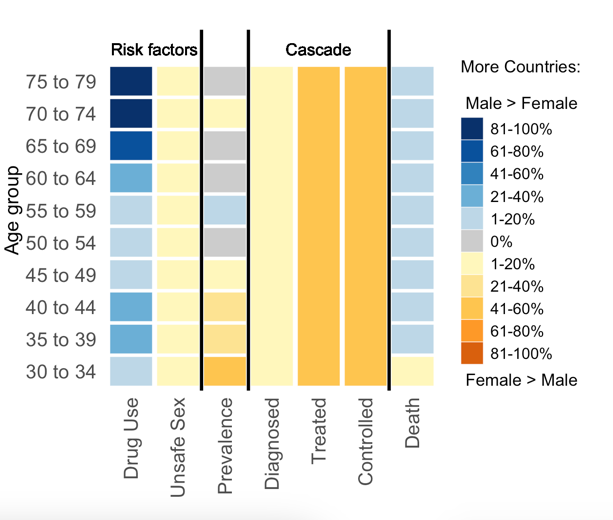  * Risk factors, prevalence, death: 44 countries. Cascade: 21 countries. | 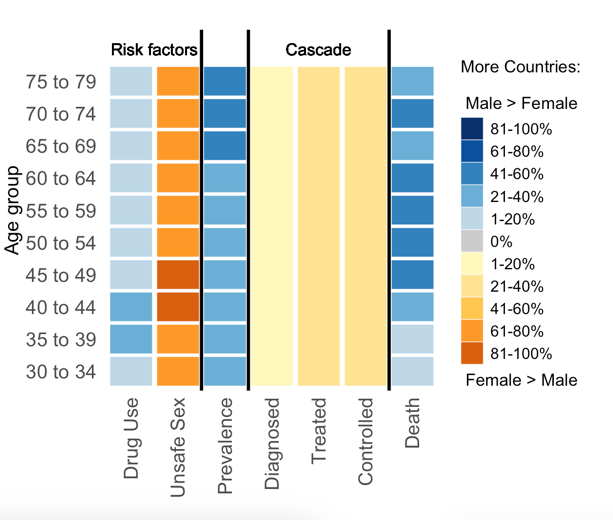  * Risk factors, prevalence, death: 22 countries. Cascade: 8 countries. |
| Latin America & the Caribbean* | East Asia and Pacific* |
| 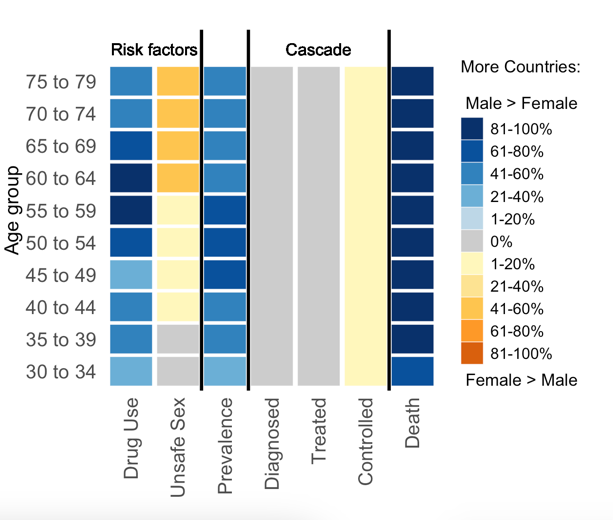  * Risk factors, prevalence, death: 38 countries. Cascade: 16 countries. | 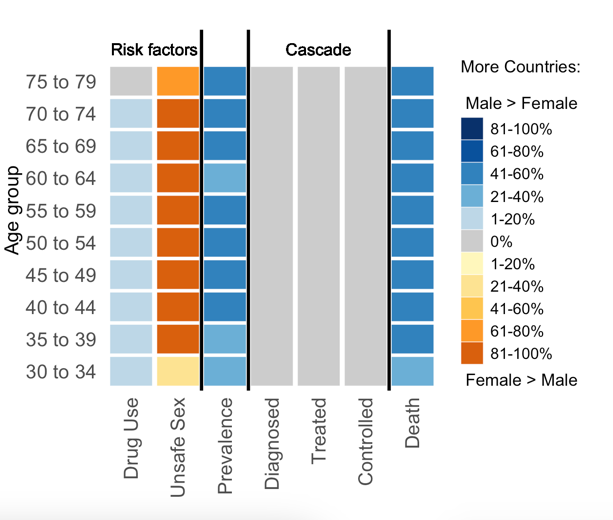  * Risk factors, prevalence, death: 34 countries. Cascade: 8 countries. |
| North America* |  |
| 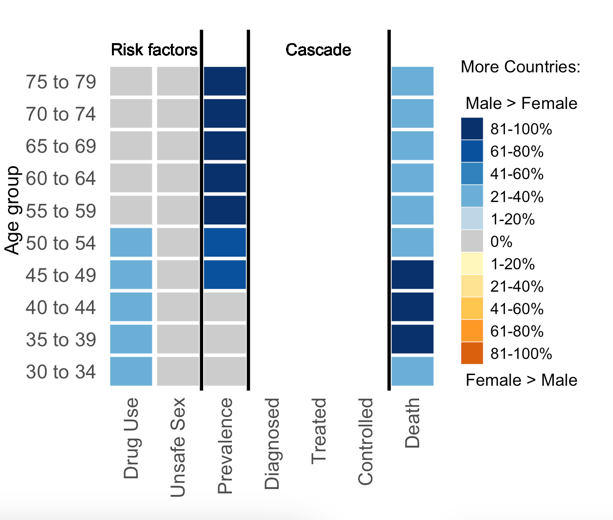  * Risk factors, prevalence, death: 3 countries. Cascade: No countries. |  |

**Fig G.** Percentages of countries with significant sex differences in global health pathways of hypertension, diabetes, and HIV and AIDS (significant when non-overlapping confidence intervals of estimates between females and males).

**Hypertension Health Pathways ***

| **Panel A** | **Panel B** |
| --- | --- |
| 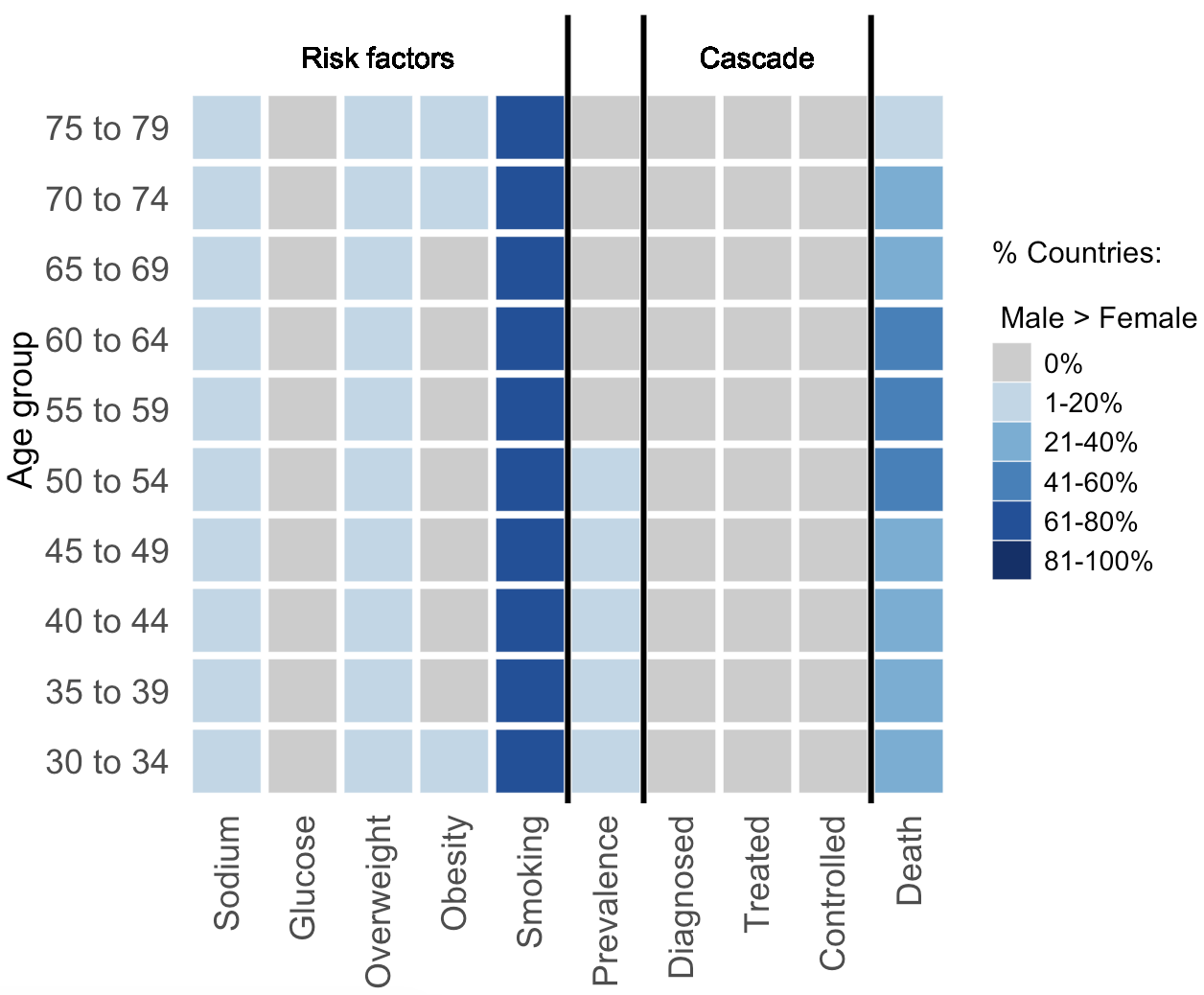 | **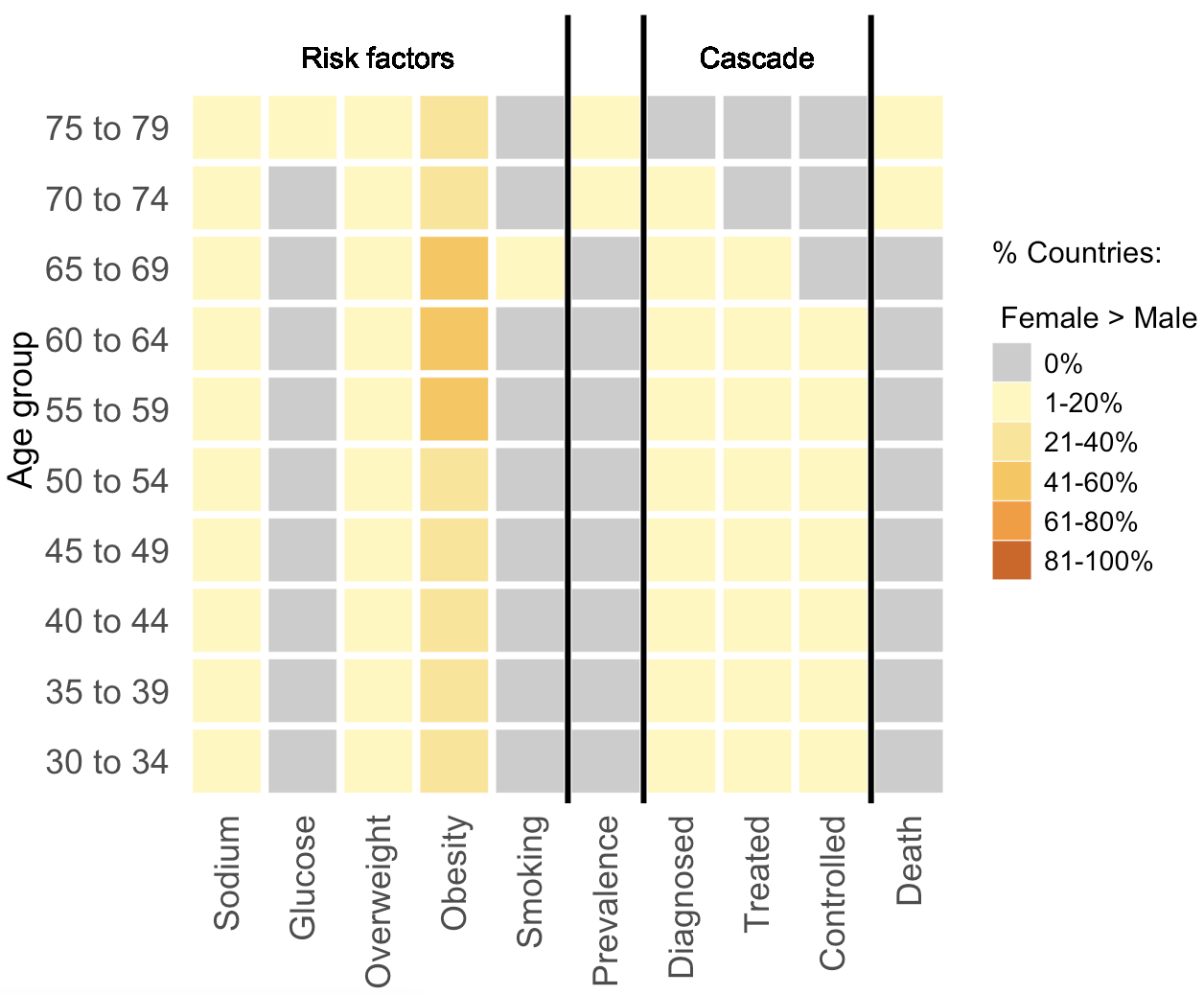** |

* Risk factors, disease prevalence, and death rates are available for 204 countries and 5-years age groups. Hypertension care cascade data are available for 200 countries and 5-years age groups.

**Diabetes Health Pathways ***

| **Panel C** | **Panel D** |
| --- | --- |
| 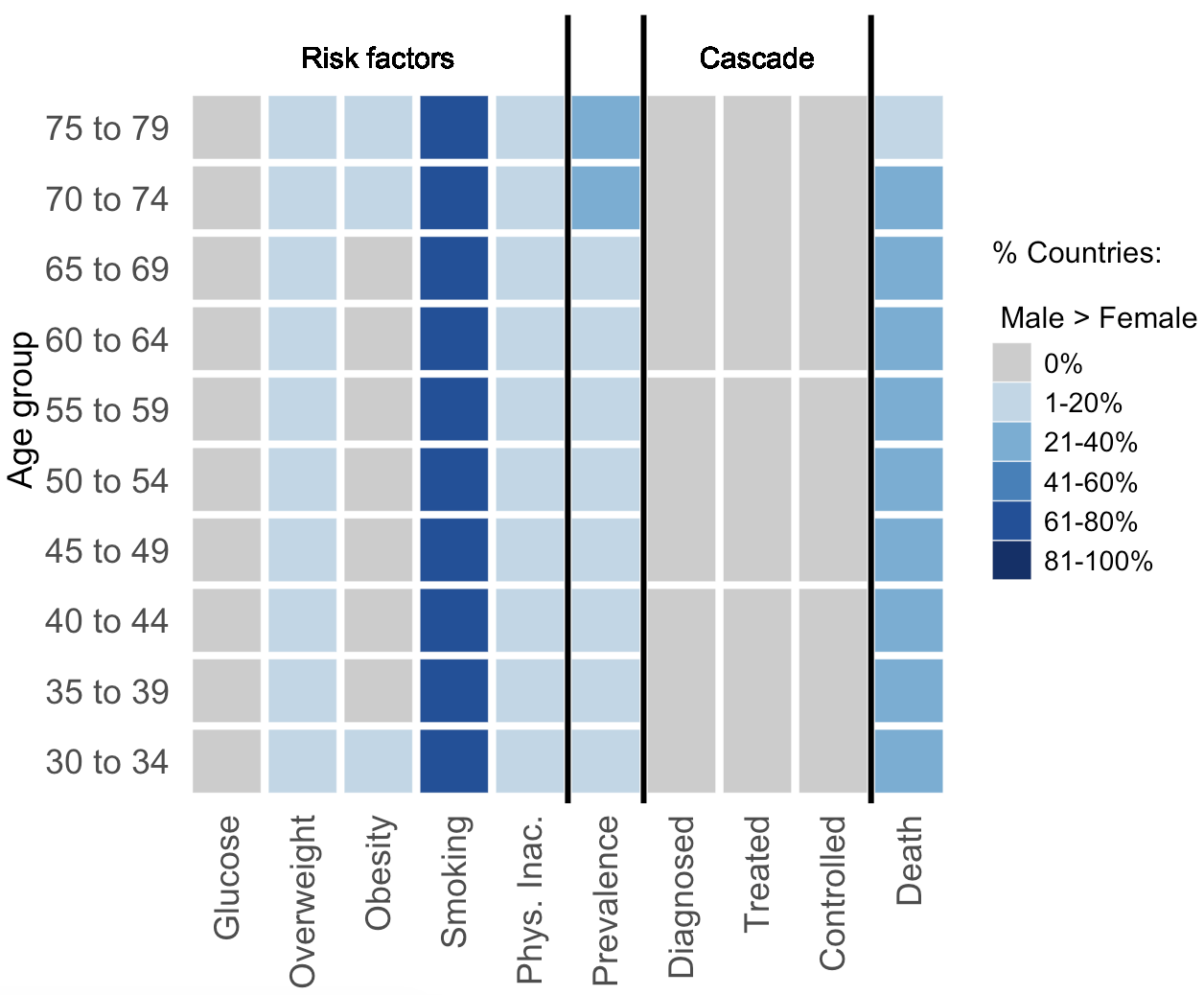 | **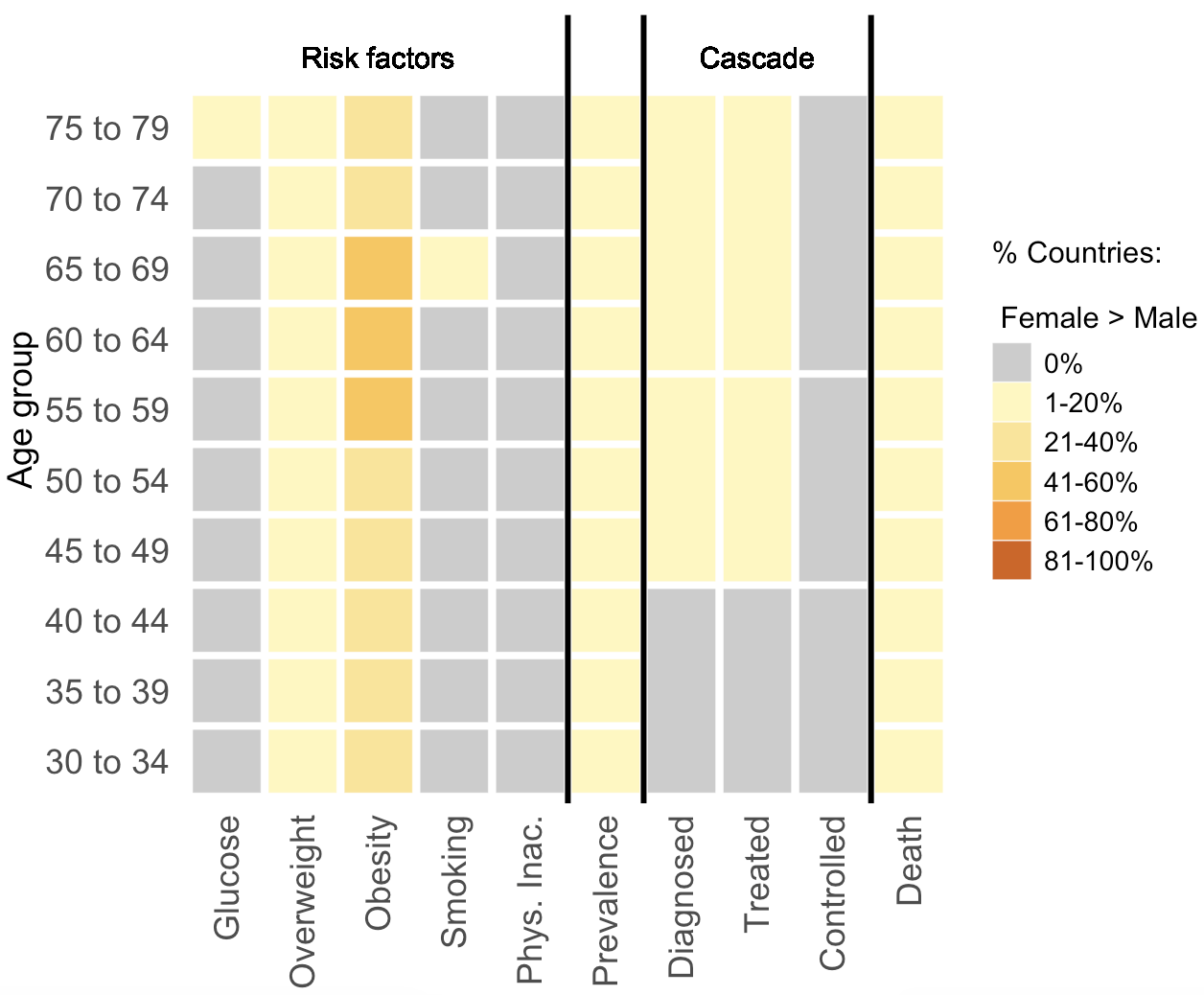** |

* Risk factors, disease prevalence, and death rates are available for 204 countries and 5-years age groups. Diabetes care cascade data are available only for 39 countries and age groups 30-44, 45-59, and 60-79. Phys. Inac.: Physical inactivity.

**HIV and AIDS Health Pathways ***

| **Panel E** | **Panel F** |
| --- | --- |
| 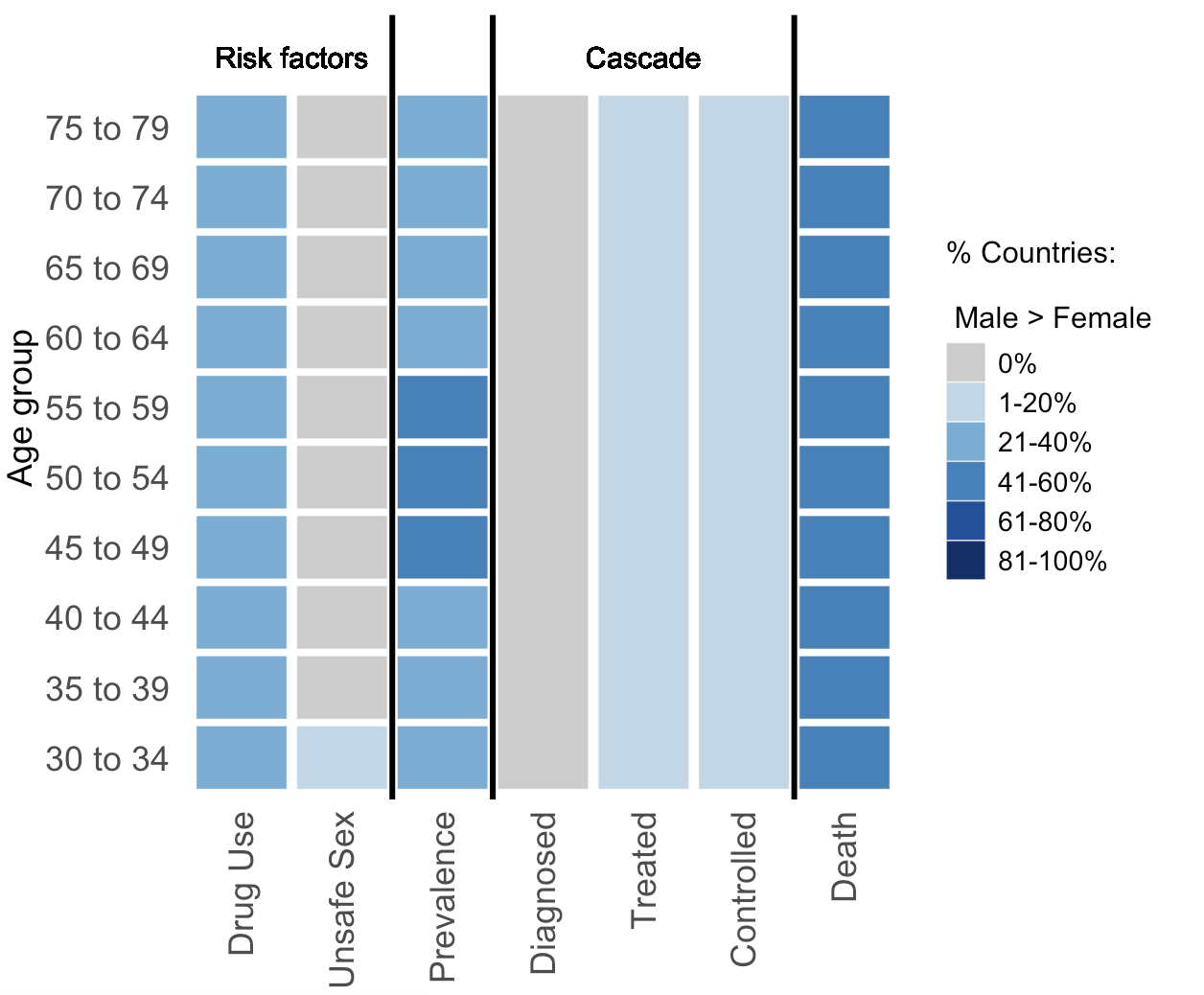 | **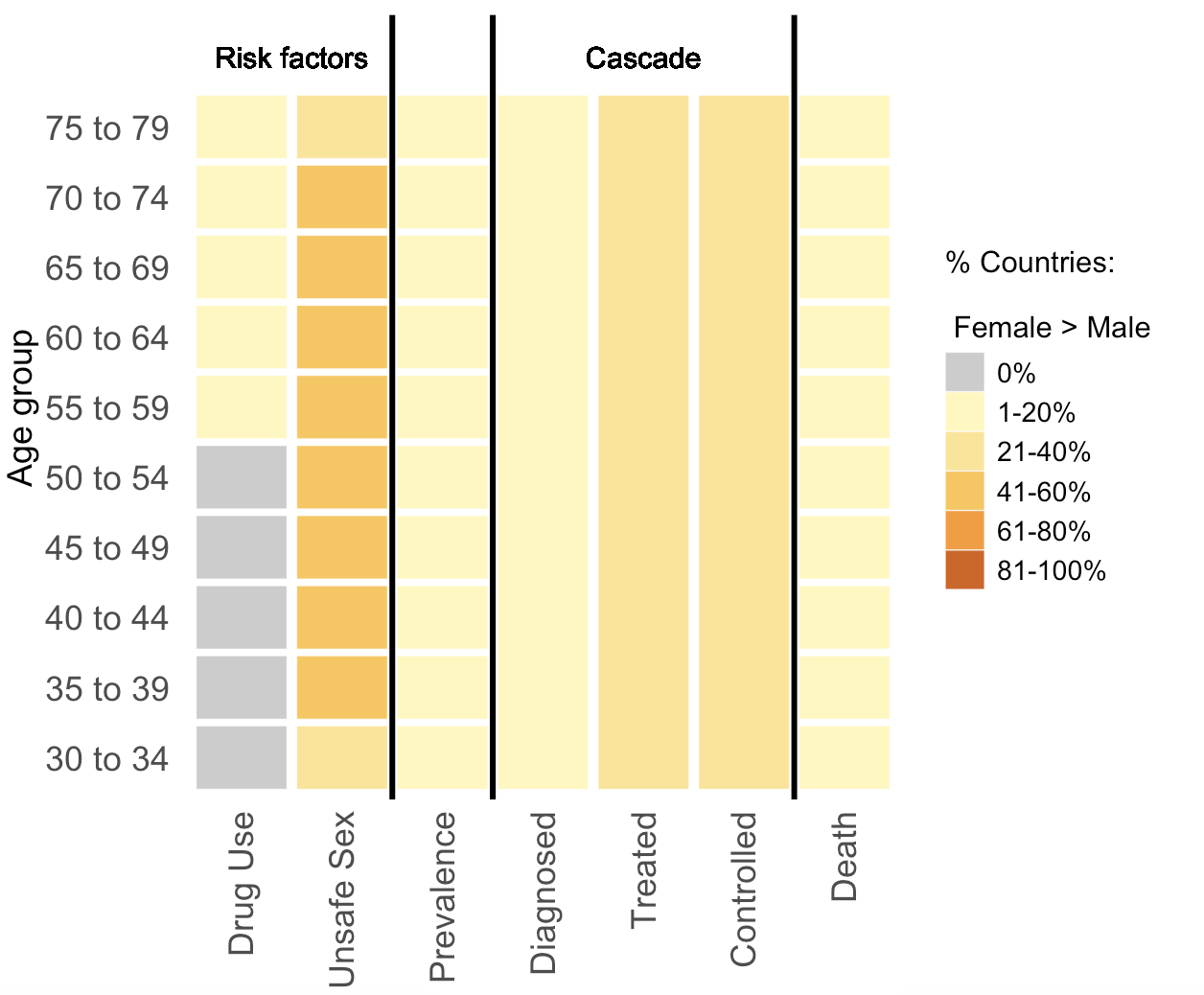** |

* Risk factors, disease prevalence, and death rates are available for 204 countries and 5-years age groups. HIV and AIDS care cascade data are available only for 76 countries and one age group (15+).

**Fig H.** Percentages of countries with significant sex differences in health pathways of hypertension, by income group (significant when non-overlapping confidence intervals of estimates between females and males).

| High-income countries* | |
| --- | --- |
| 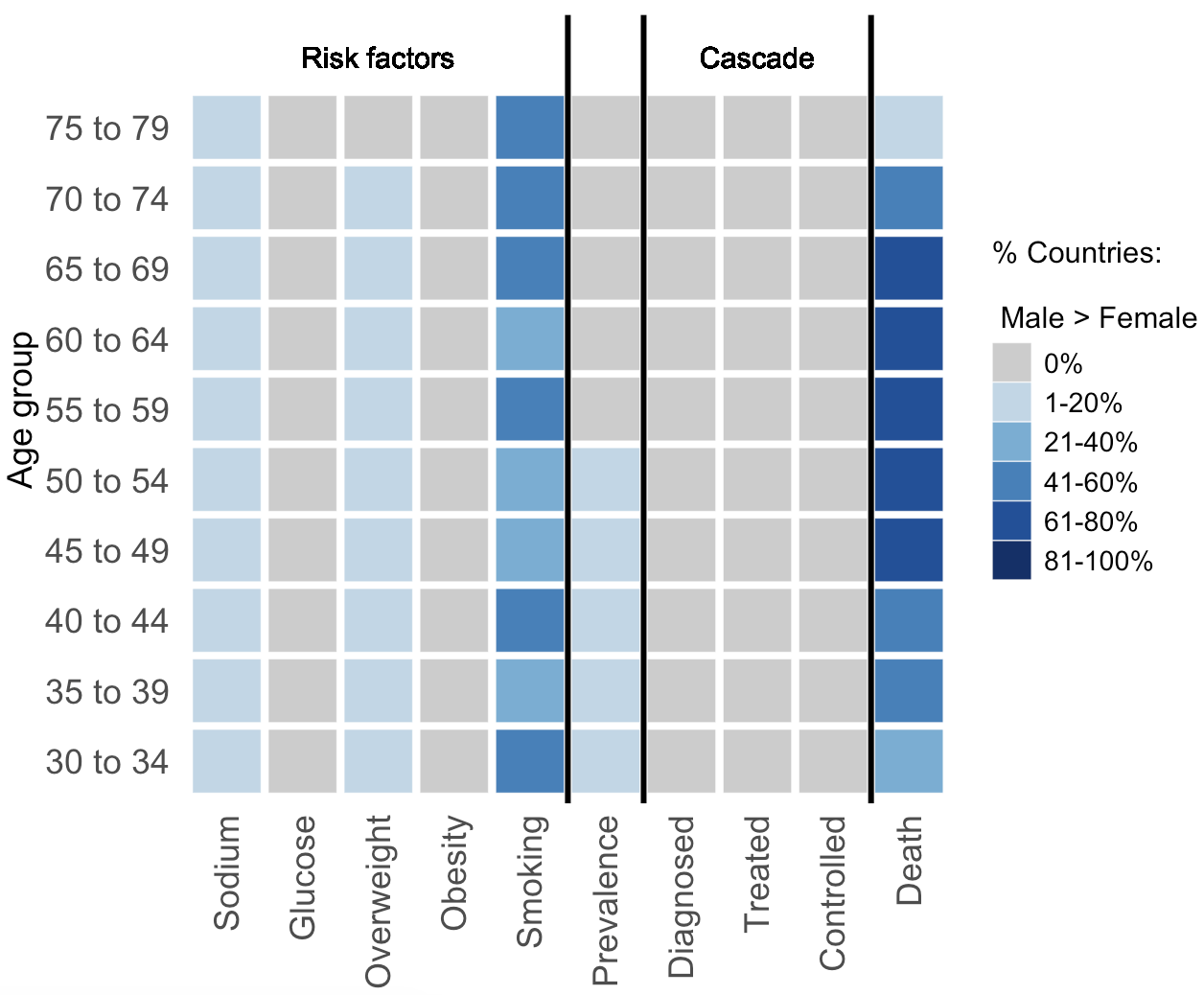 | 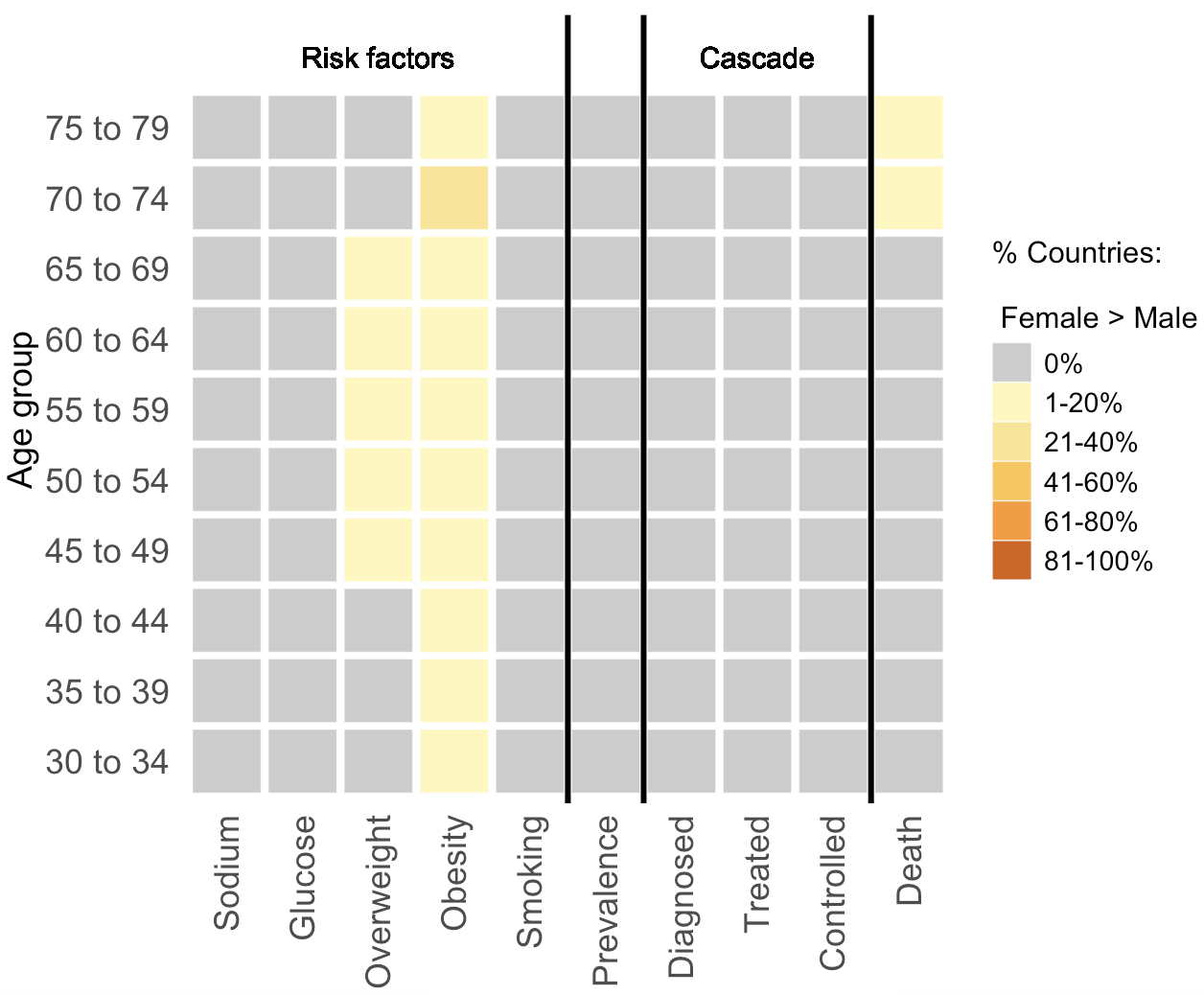 |
| * Risk factors, prevalence, death: 67 countries. Cascade: 63 countries. | |
| Upper-middle-income countries* | |
| 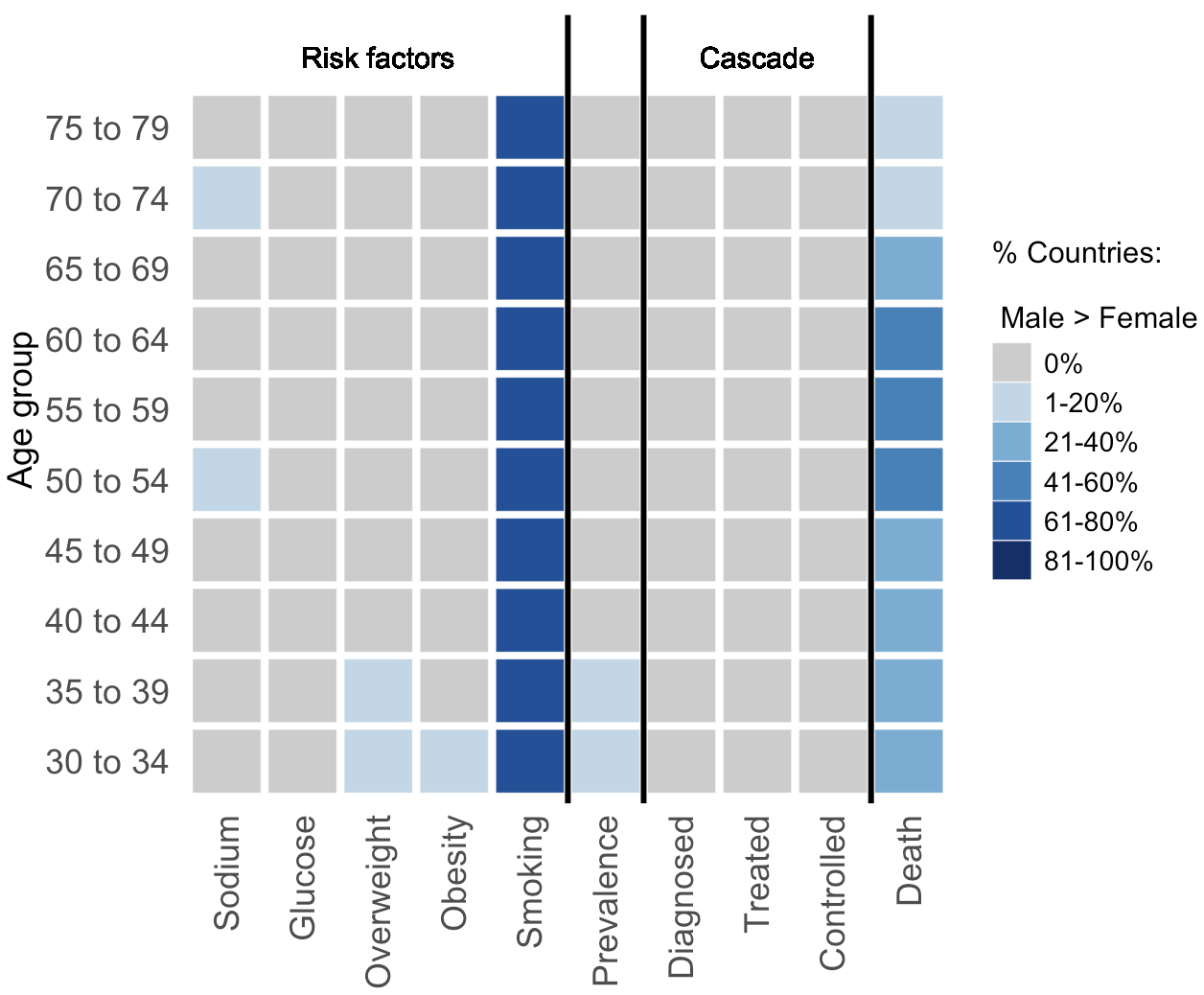 | 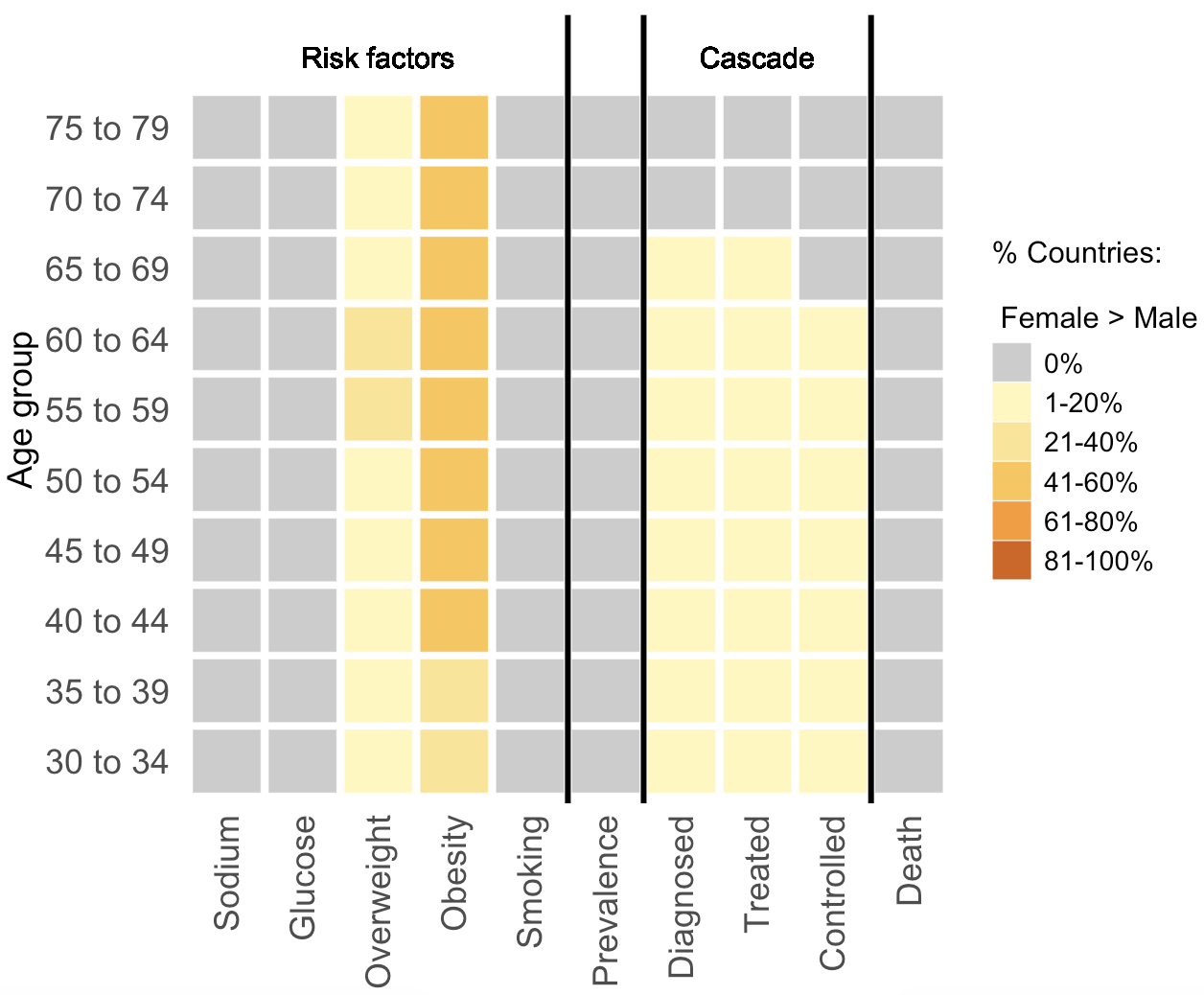 |
| * Risk factors, prevalence, death: 54 countries. Cascade: 54 countries. | |
| Lower-middle-income countries* | |
| 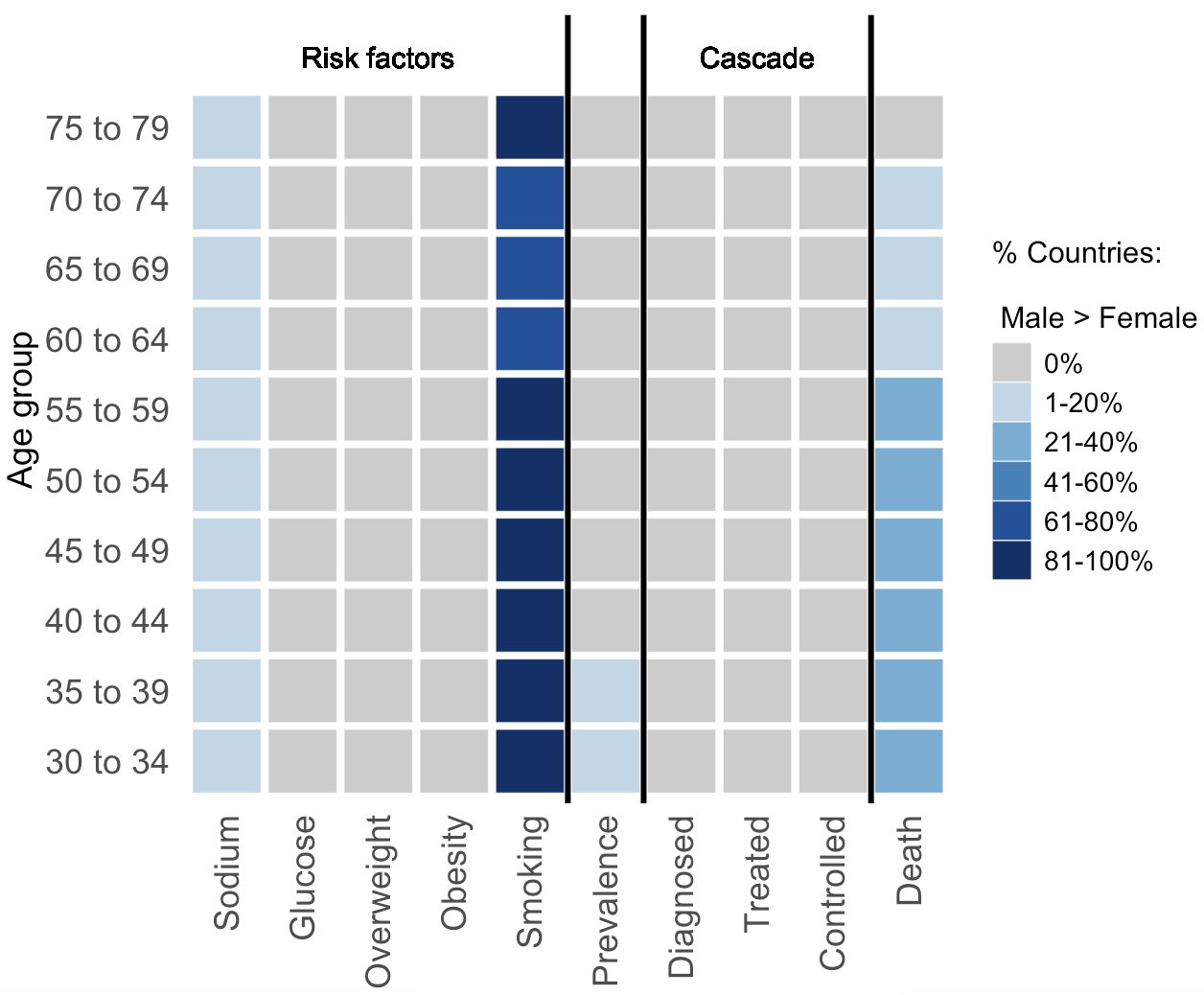 | 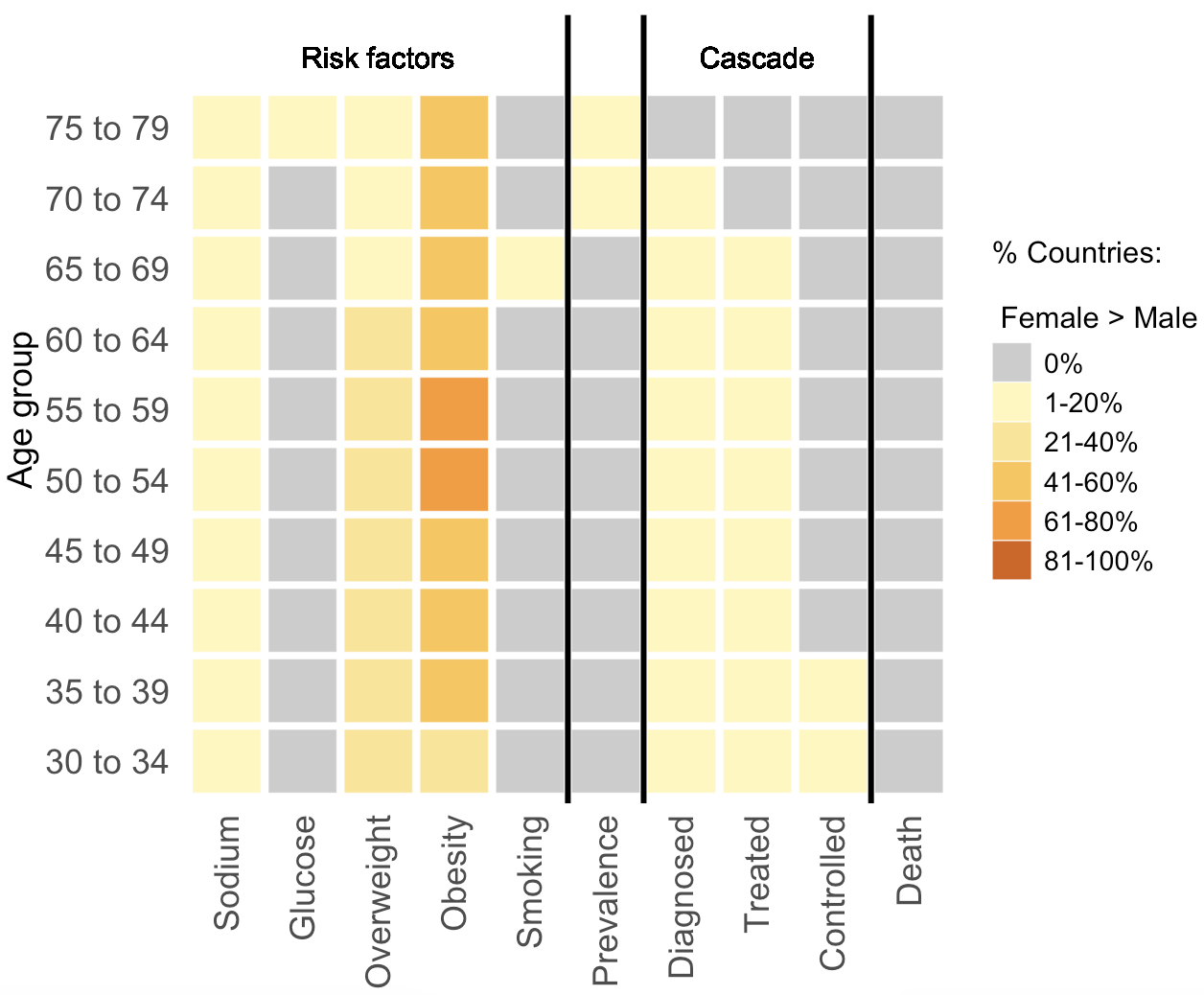 |
| * Risk factors, prevalence, death: 54 countries. Cascade: 54 countries. | |
| Low-income countries* | |
| 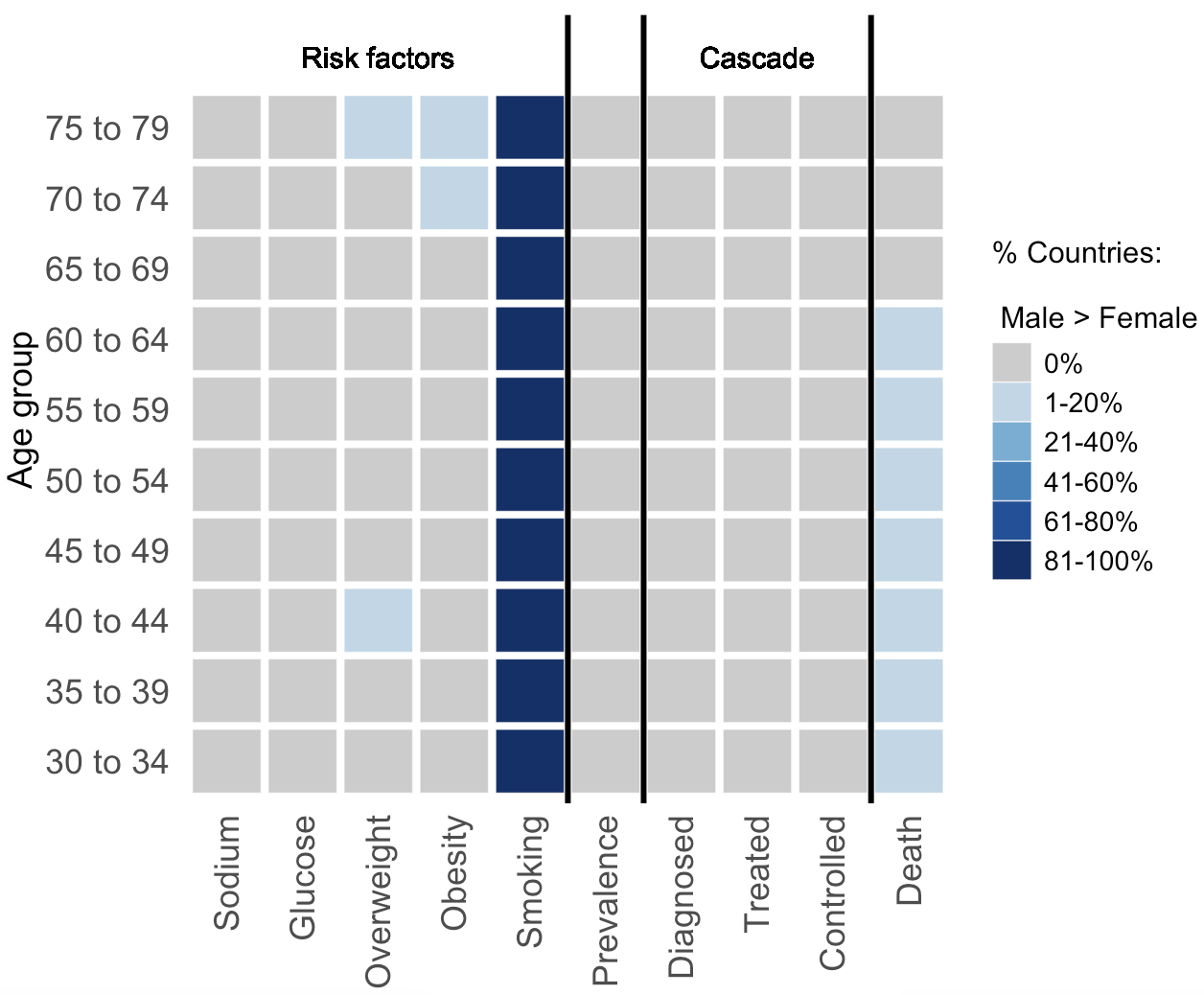 | 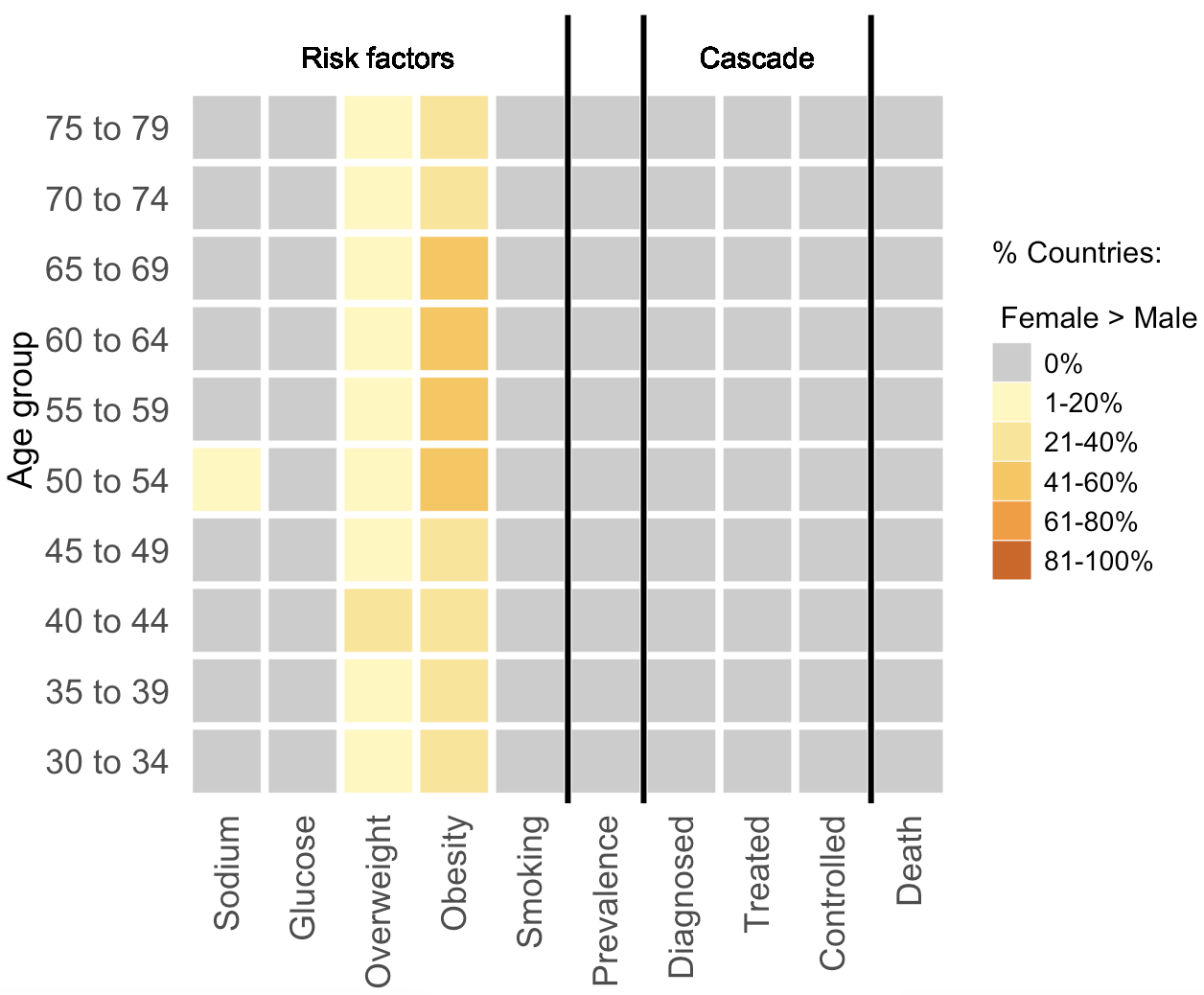 |
| * Risk factors, prevalence, death: 26 countries. Cascade: 26 countries. | |

**Fig I.** Percentages of countries with significant sex differences in health pathways of hypertension, by region (significant when non-overlapping confidence intervals of estimates between females and males).

| Europe and Central Asia* | |
| --- | --- |
| 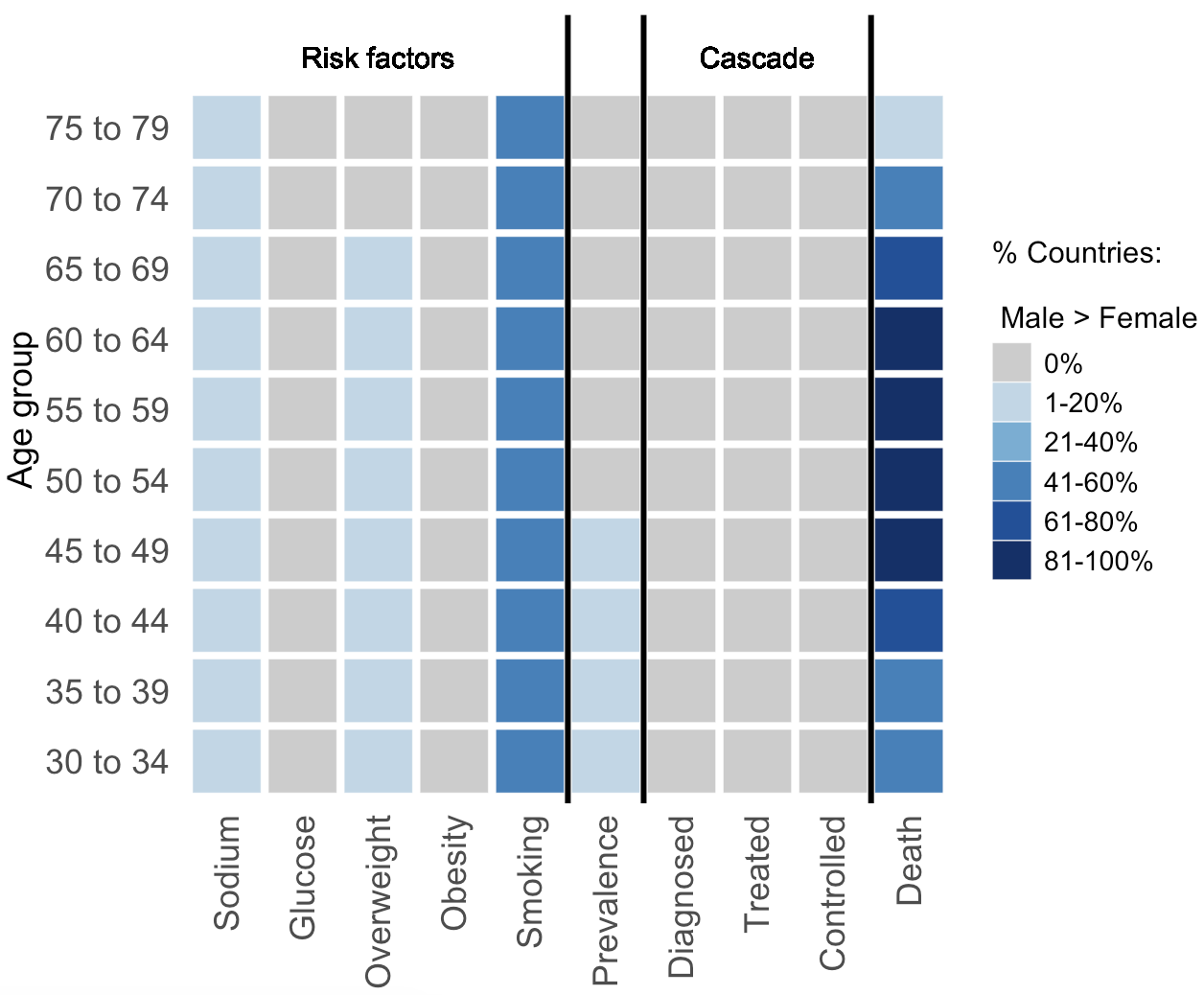 | 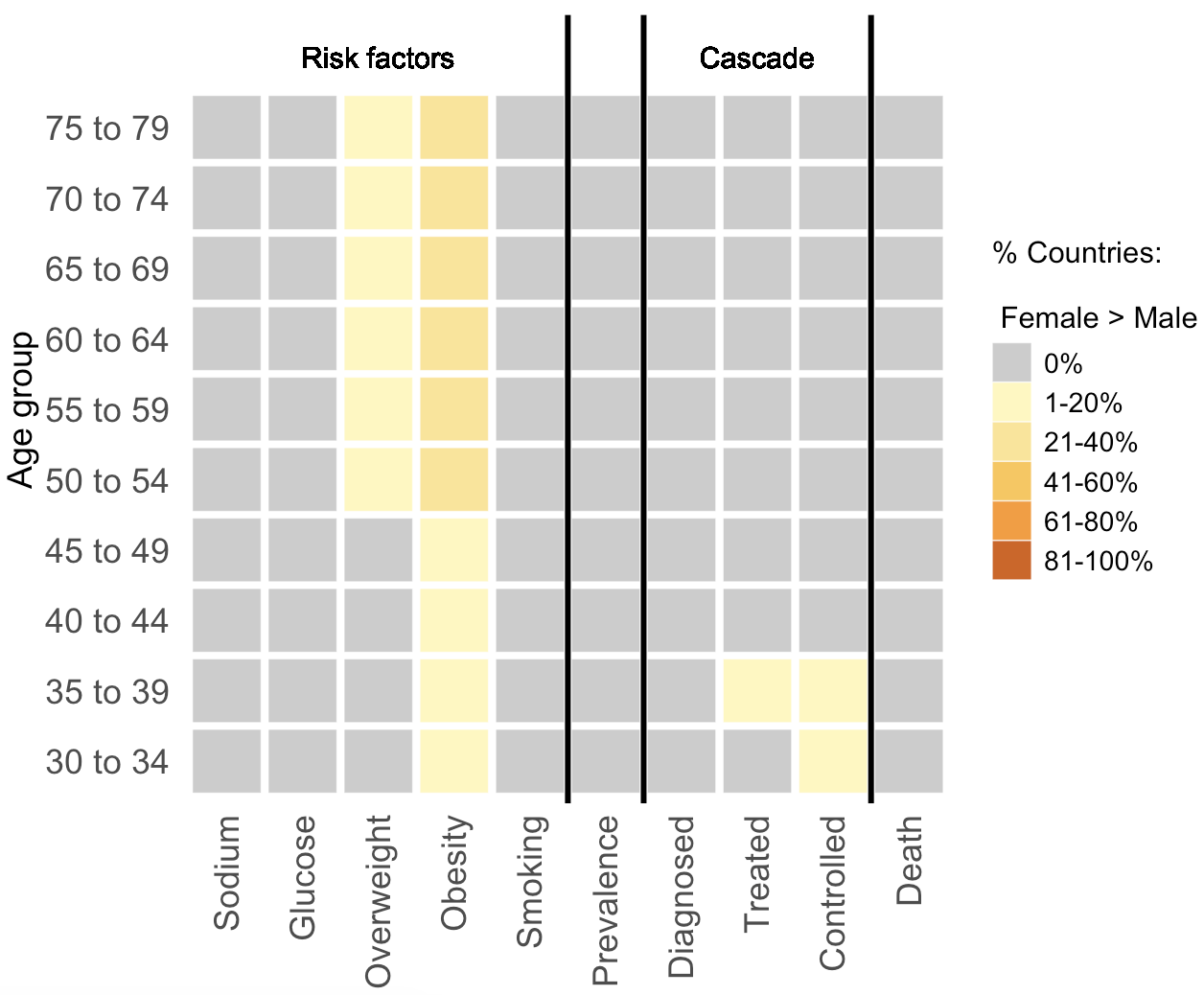 |
| * Risk factors, prevalence, death: 52 countries. Cascade: 50 countries. | |
| South Asia* | |
| 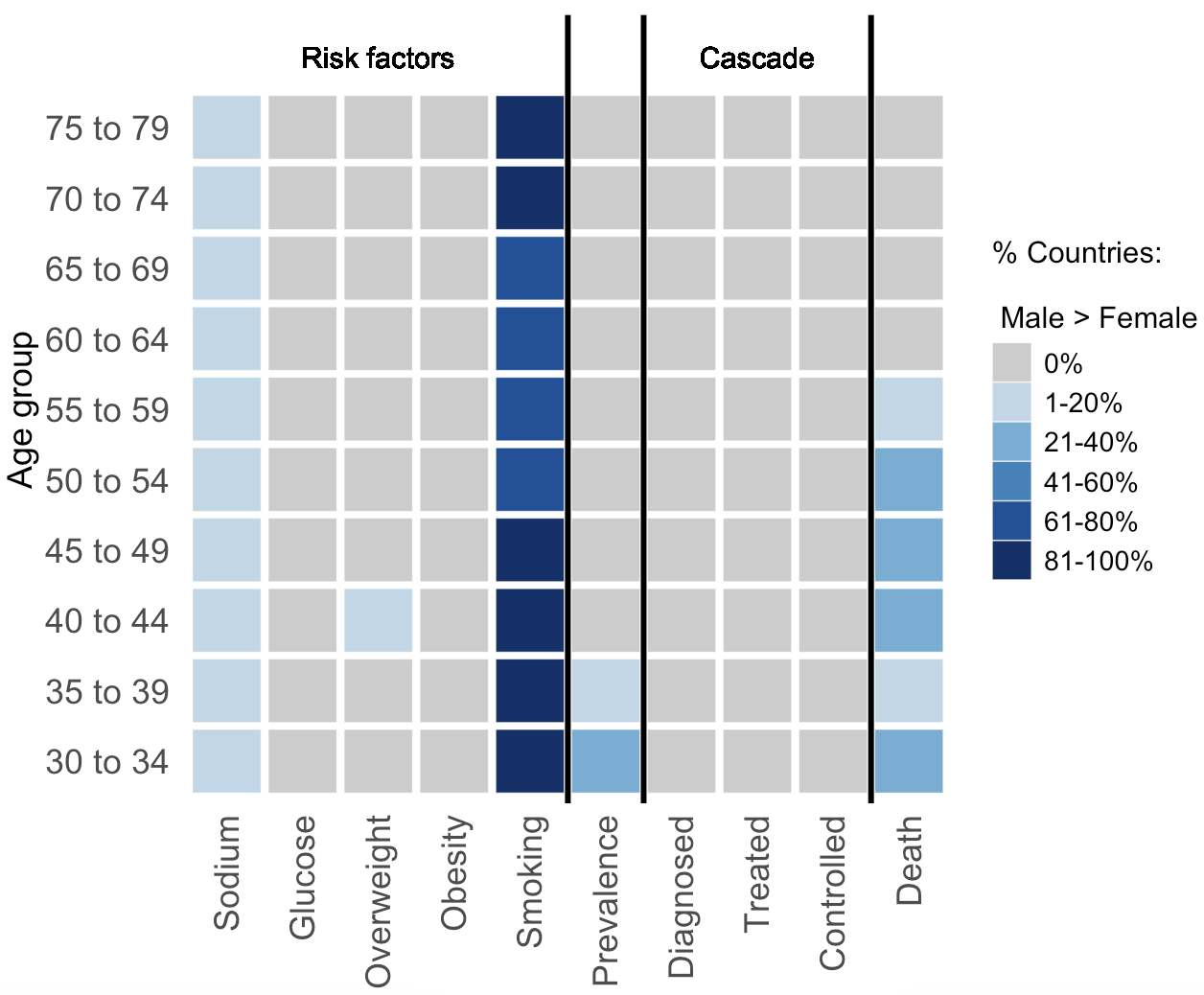 | 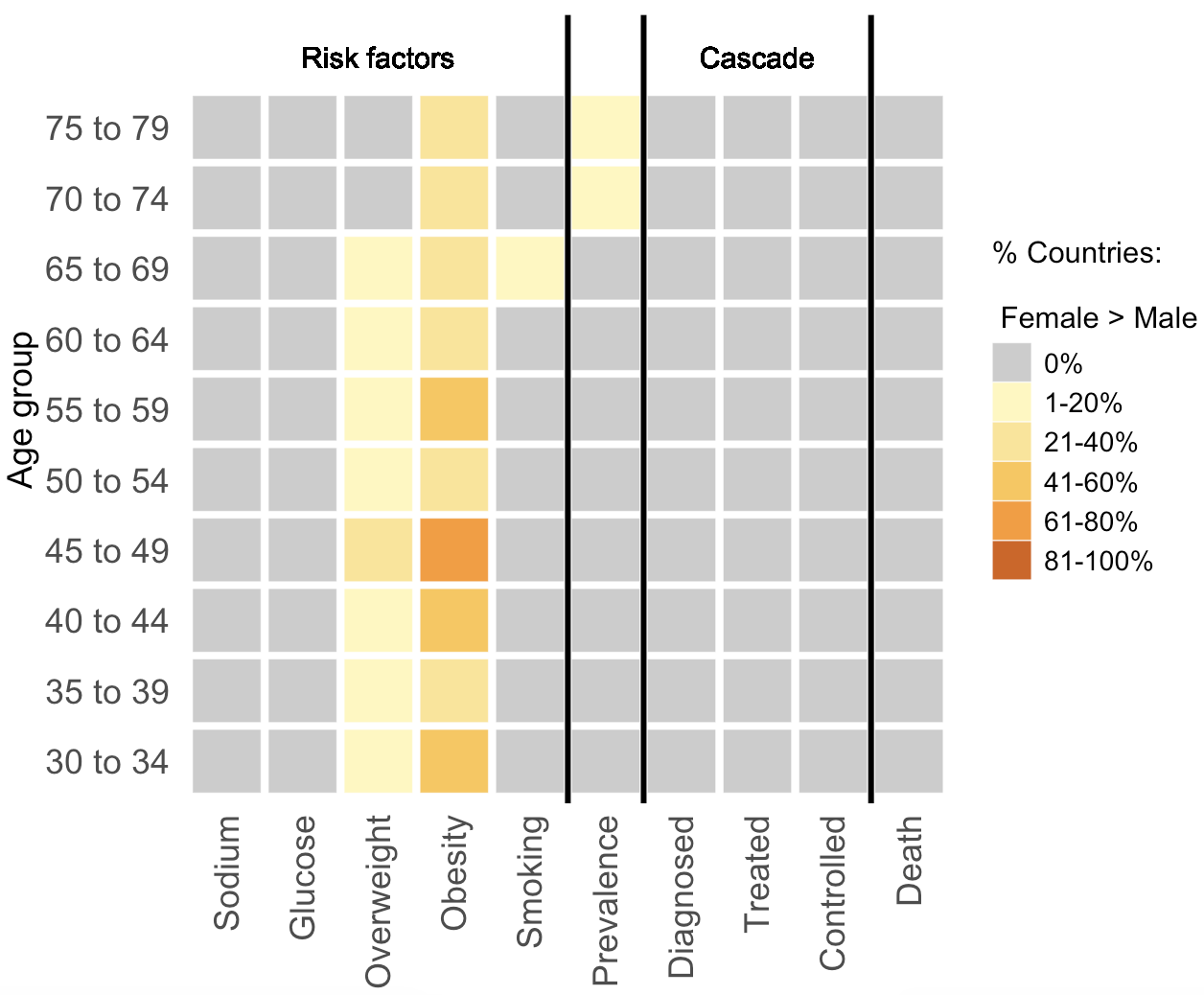 |
| * Risk factors, prevalence, death: 8 countries. Cascade: 8 countries. | |
| Sub-Saharan Africa* | |
| 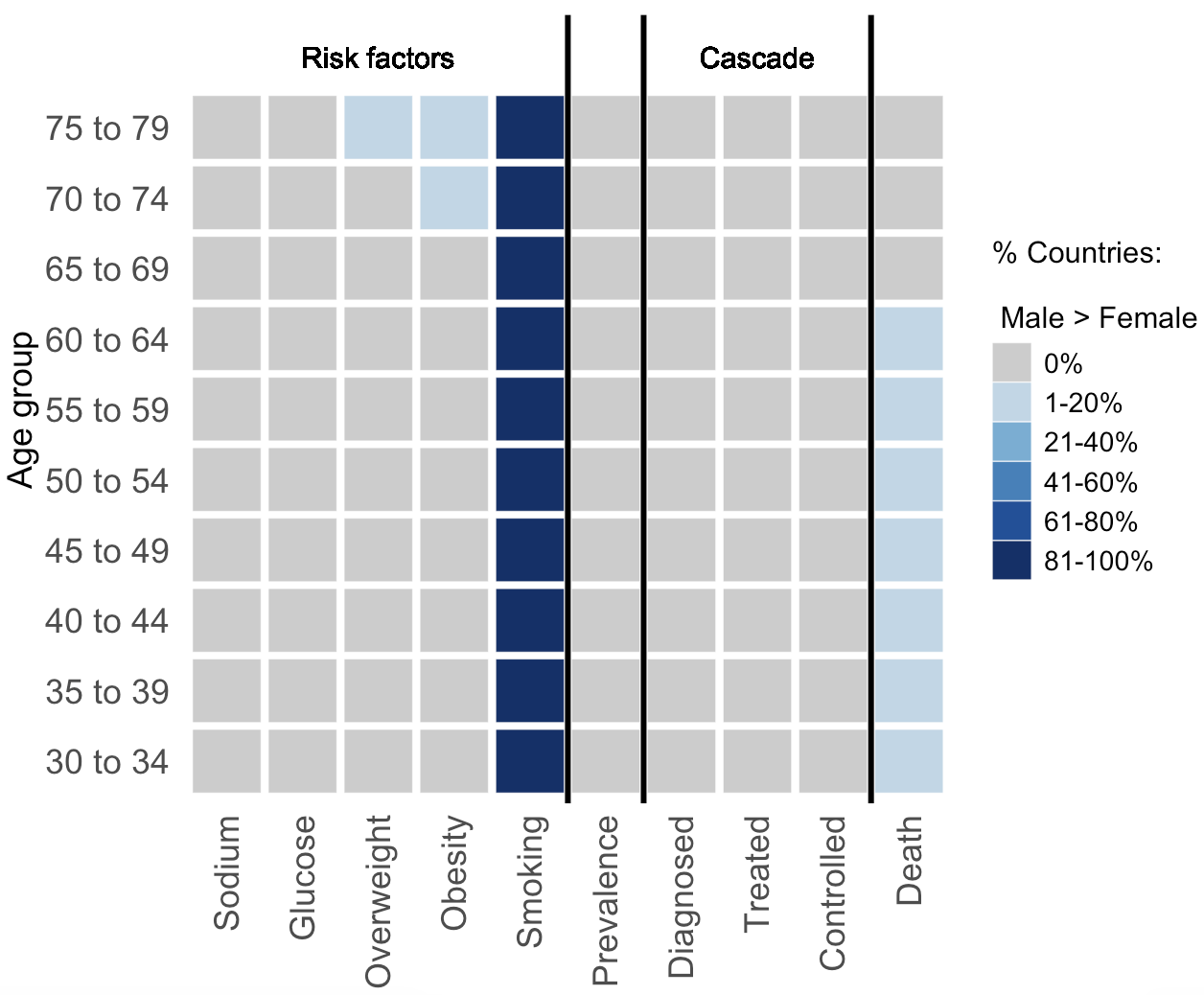 | 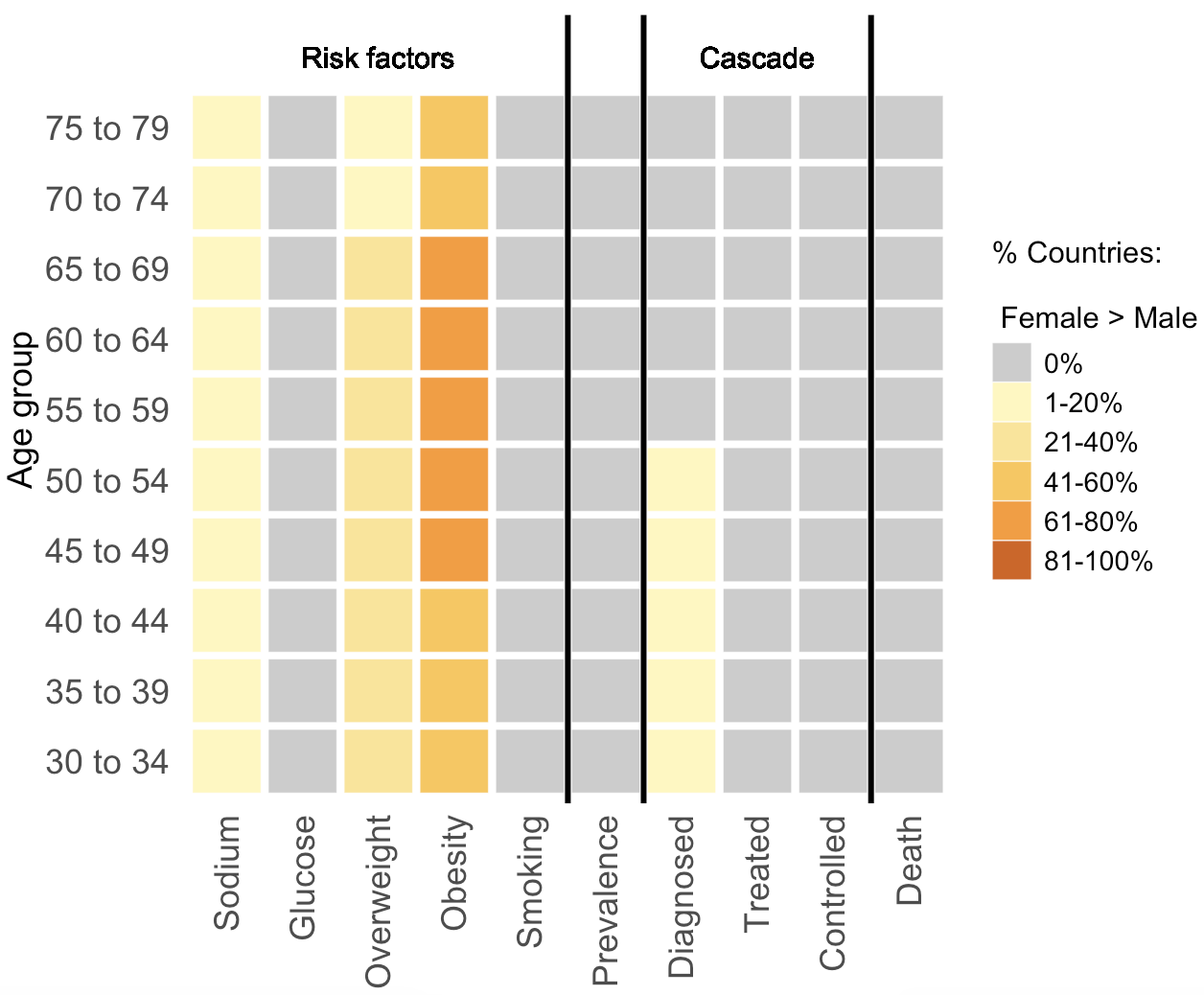 |
| * Risk factors, prevalence, death: 44 countries. Cascade: 44 countries. | |
| Middle East and North Africa* | |
| 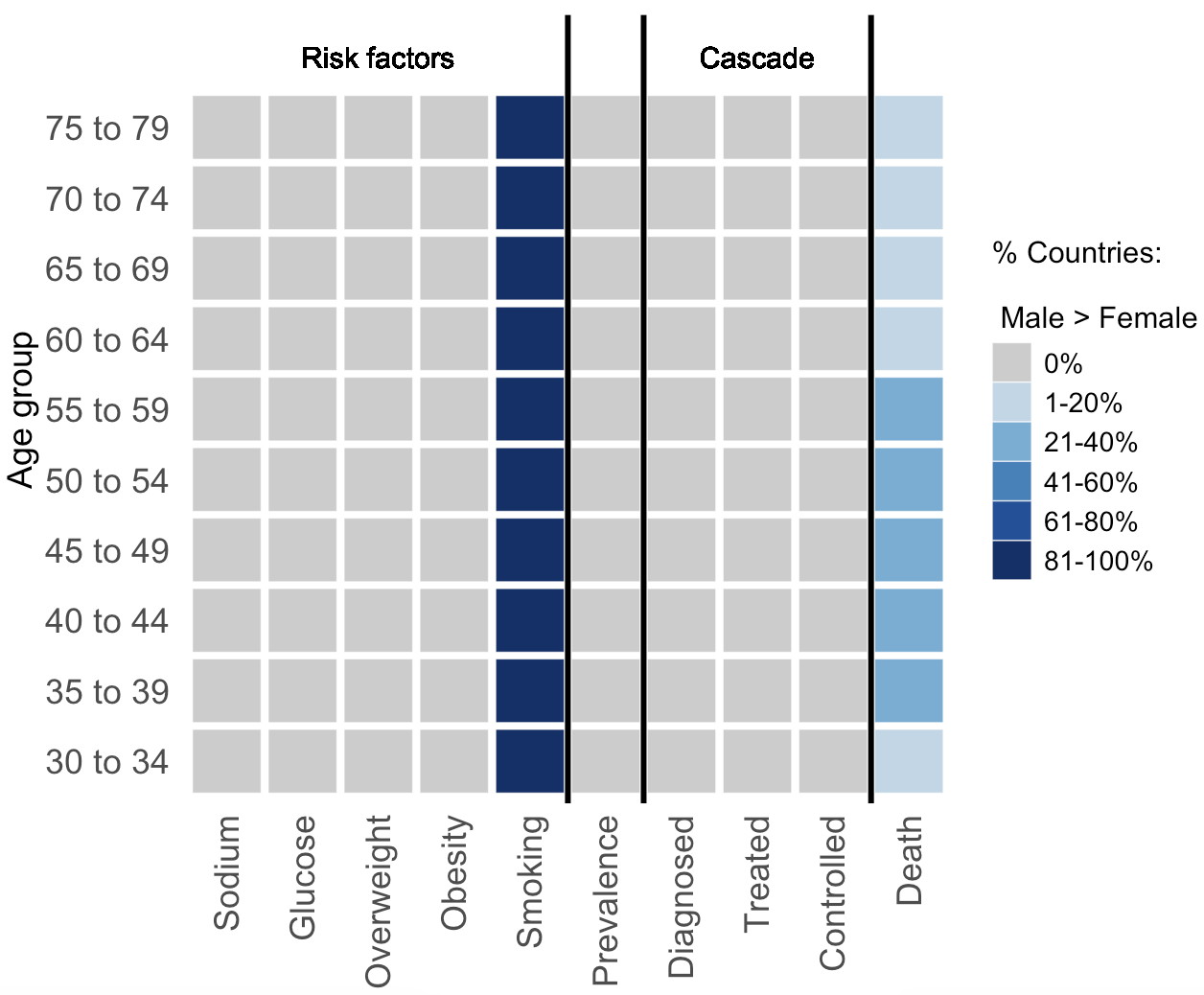 | 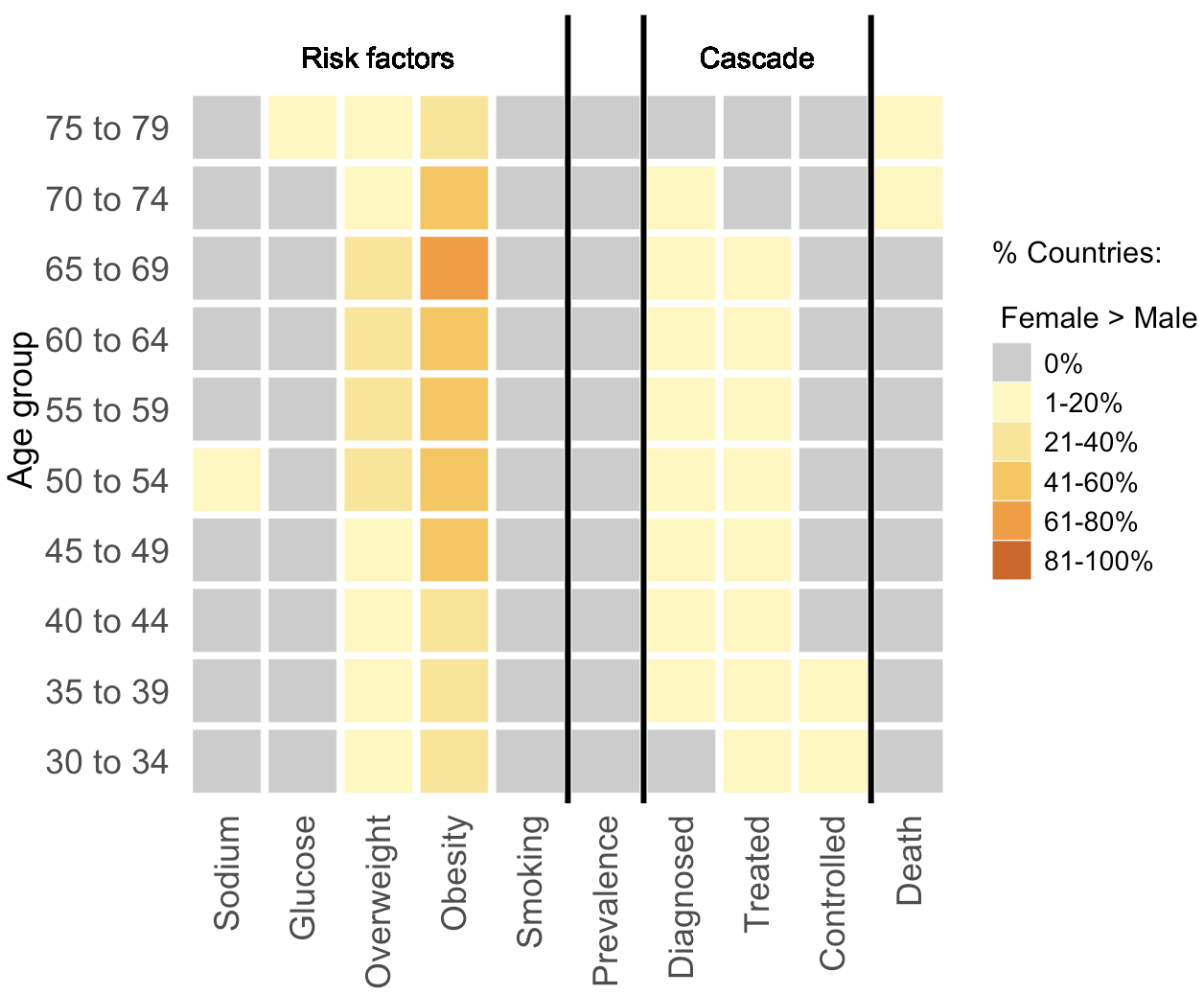 |
| * Risk factors, prevalence, death: 22 countries. Cascade: 22 countries. | |
| Latin America & the Caribbean* | |
| 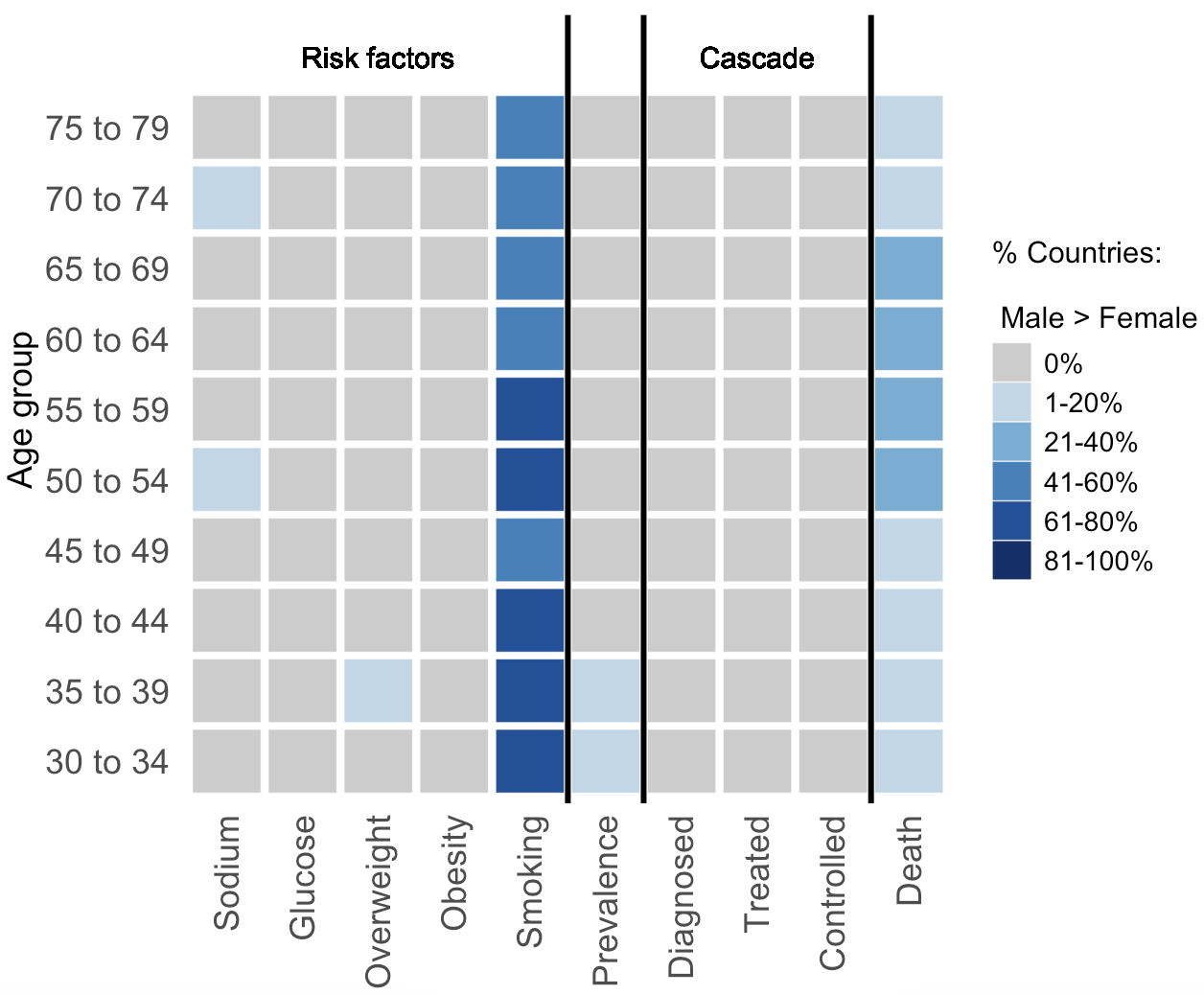 | 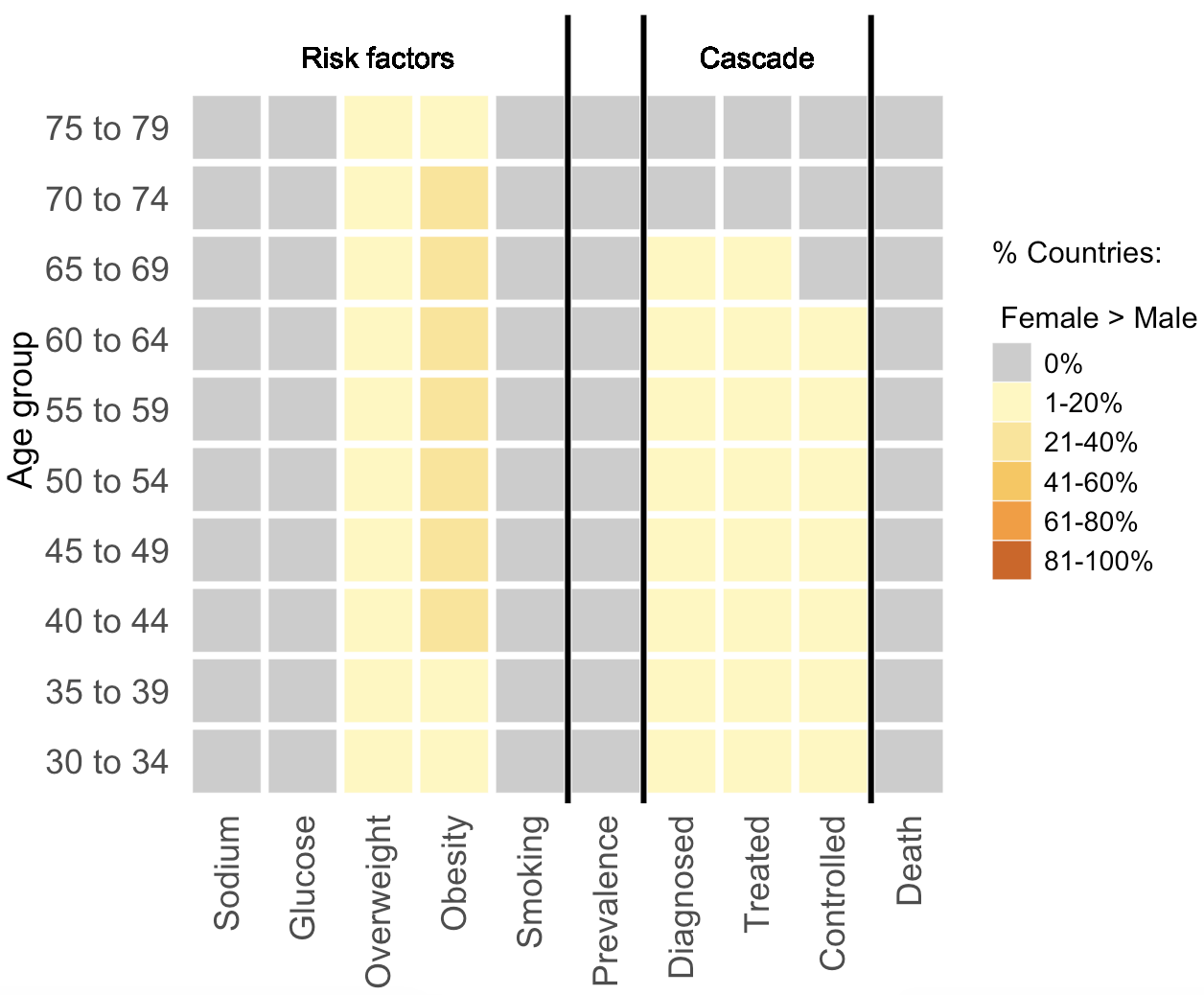 |
| * Risk factors, prevalence, death: 38 countries. Cascade: 37 countries. | |
| East Asia and Pacific* | |
| 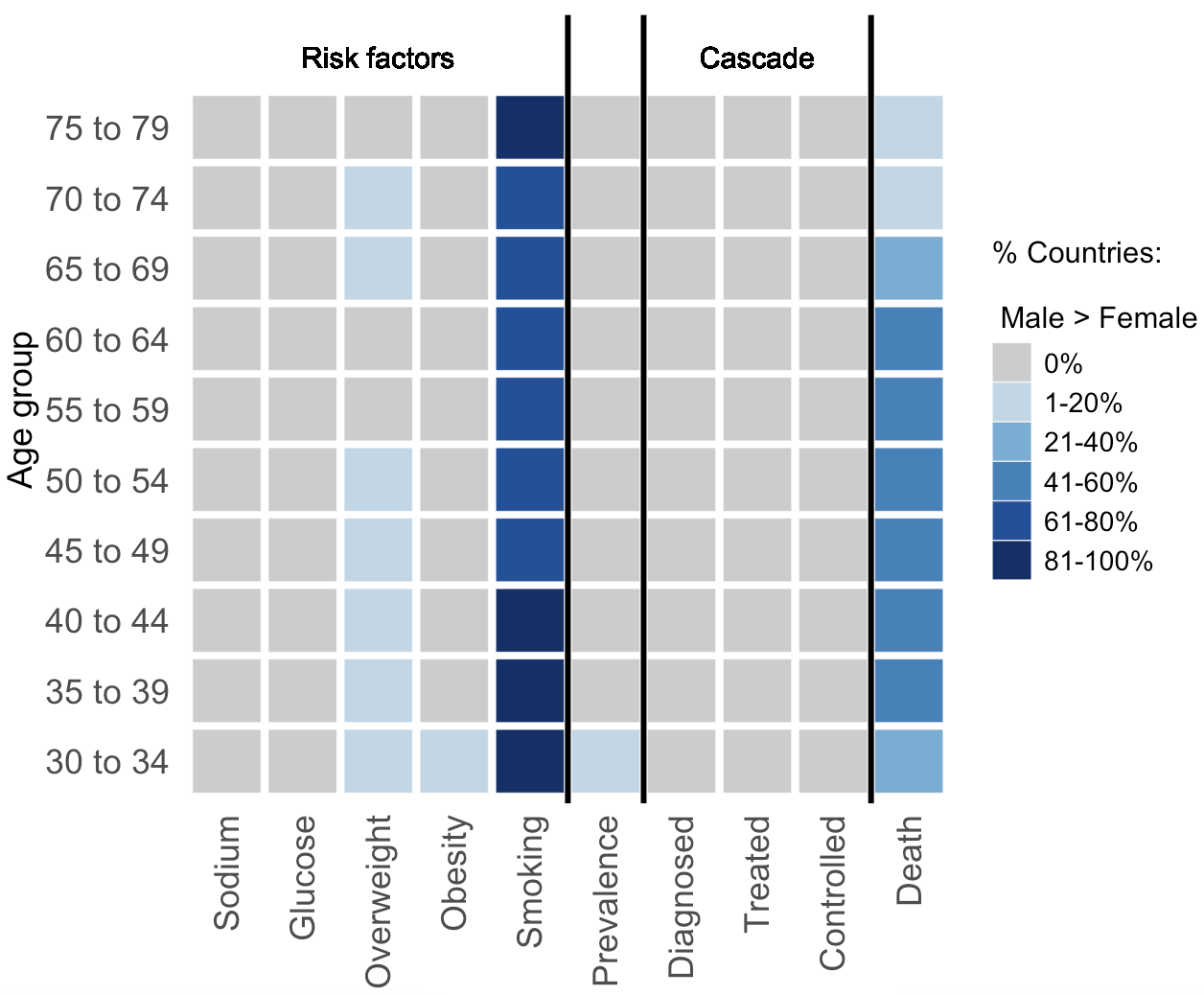 | 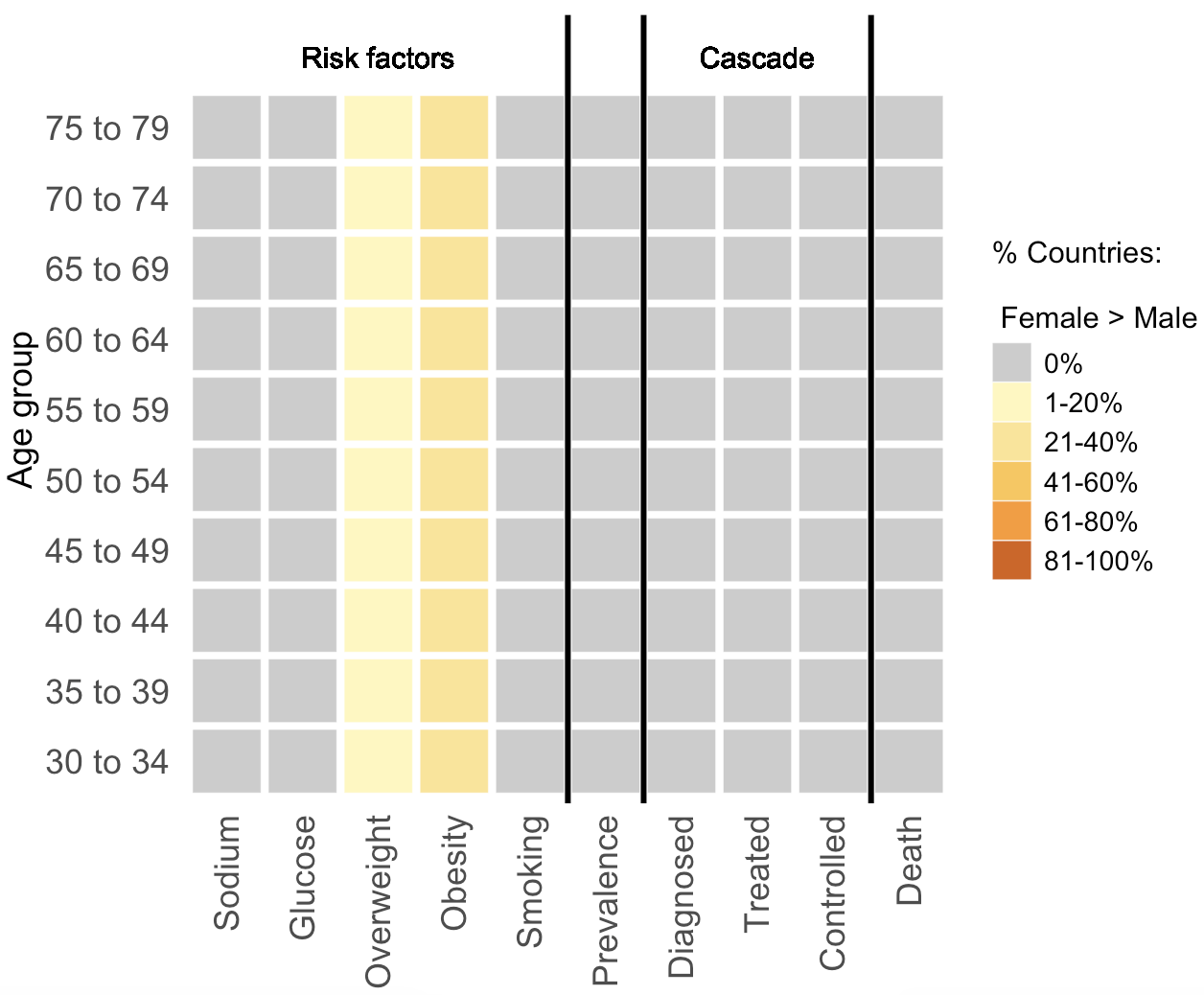 |
| * Risk factors, prevalence, death: 34 countries. Cascade: 33 countries. | |
| North America* | |
| 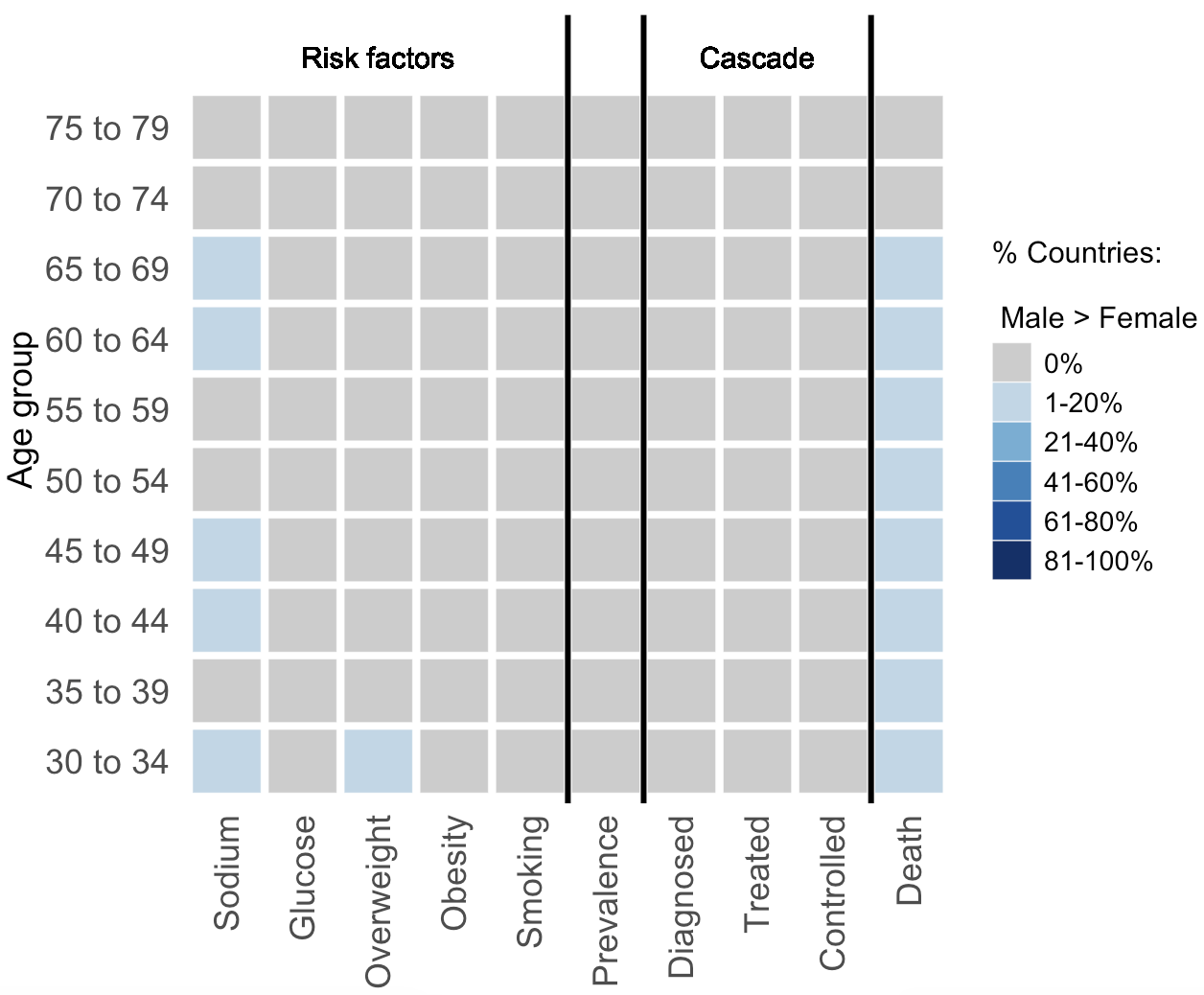 | 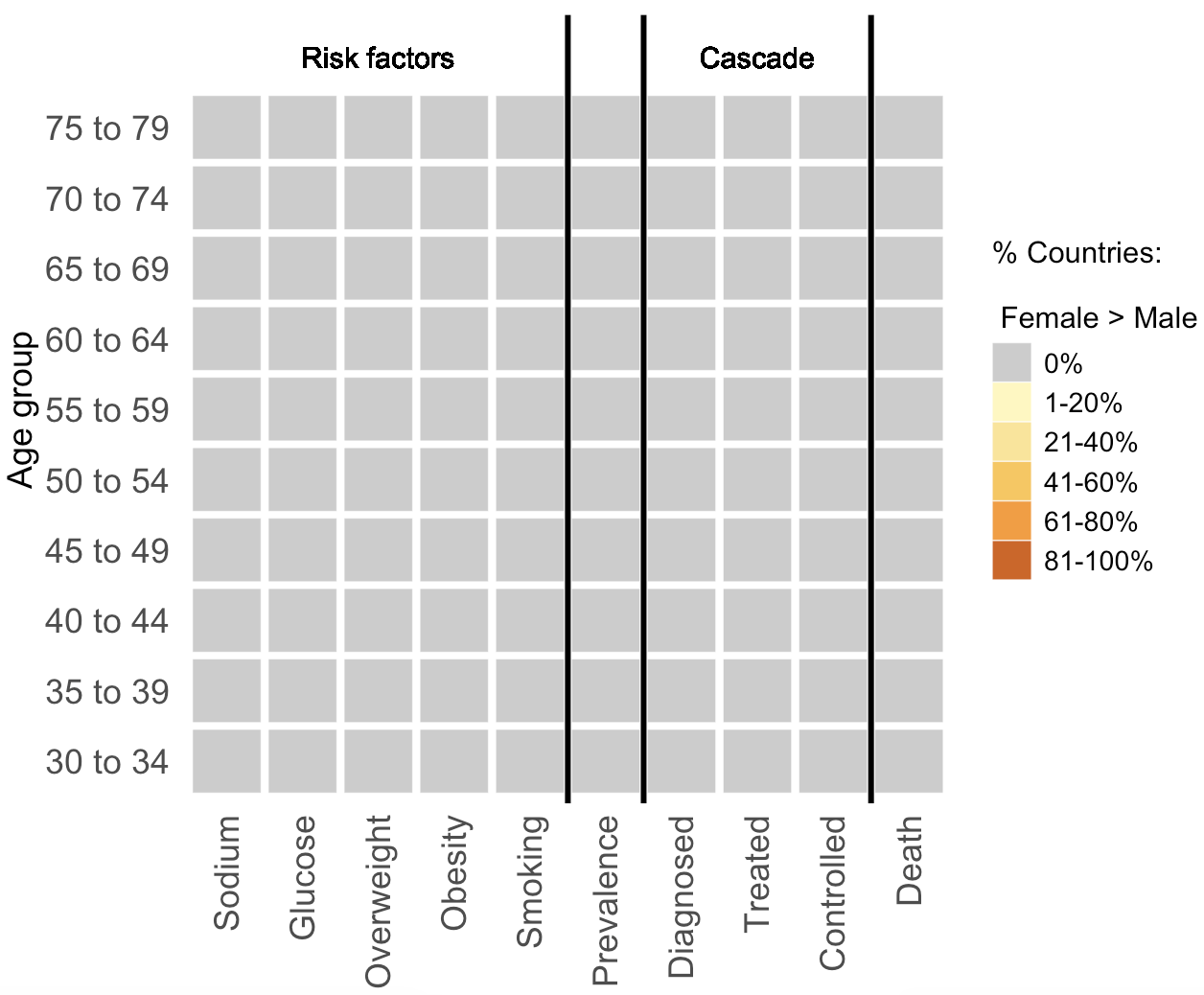 |
| * Risk factors, prevalence, death: 3 countries. Cascade: 3 countries. | |

**Fig J.** Percentages of countries with significant sex differences in health pathways of diabetes, by income group (significant when non-overlapping confidence intervals of estimates between females and males).

| High-income countries* | |
| --- | --- |
| 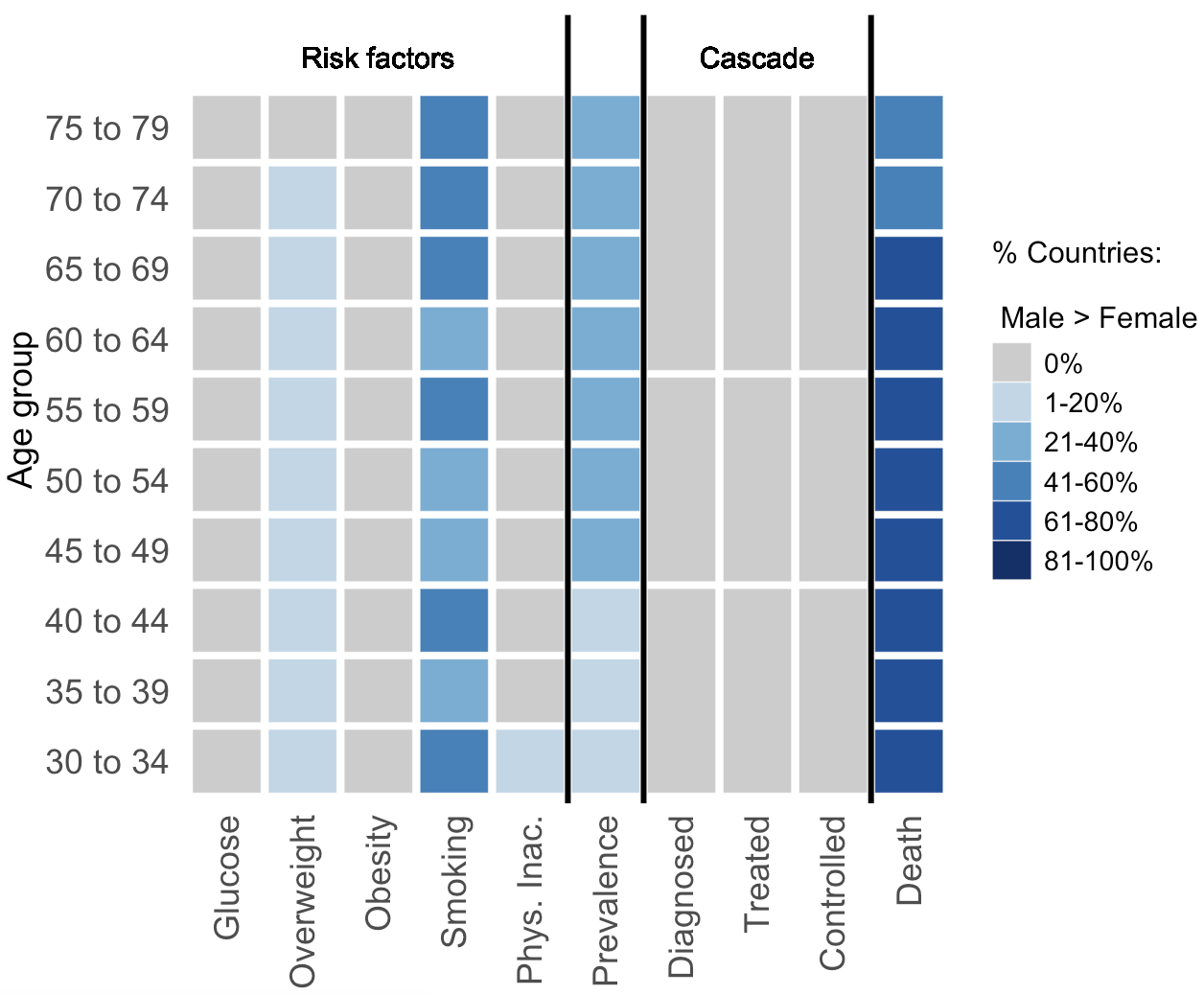 | 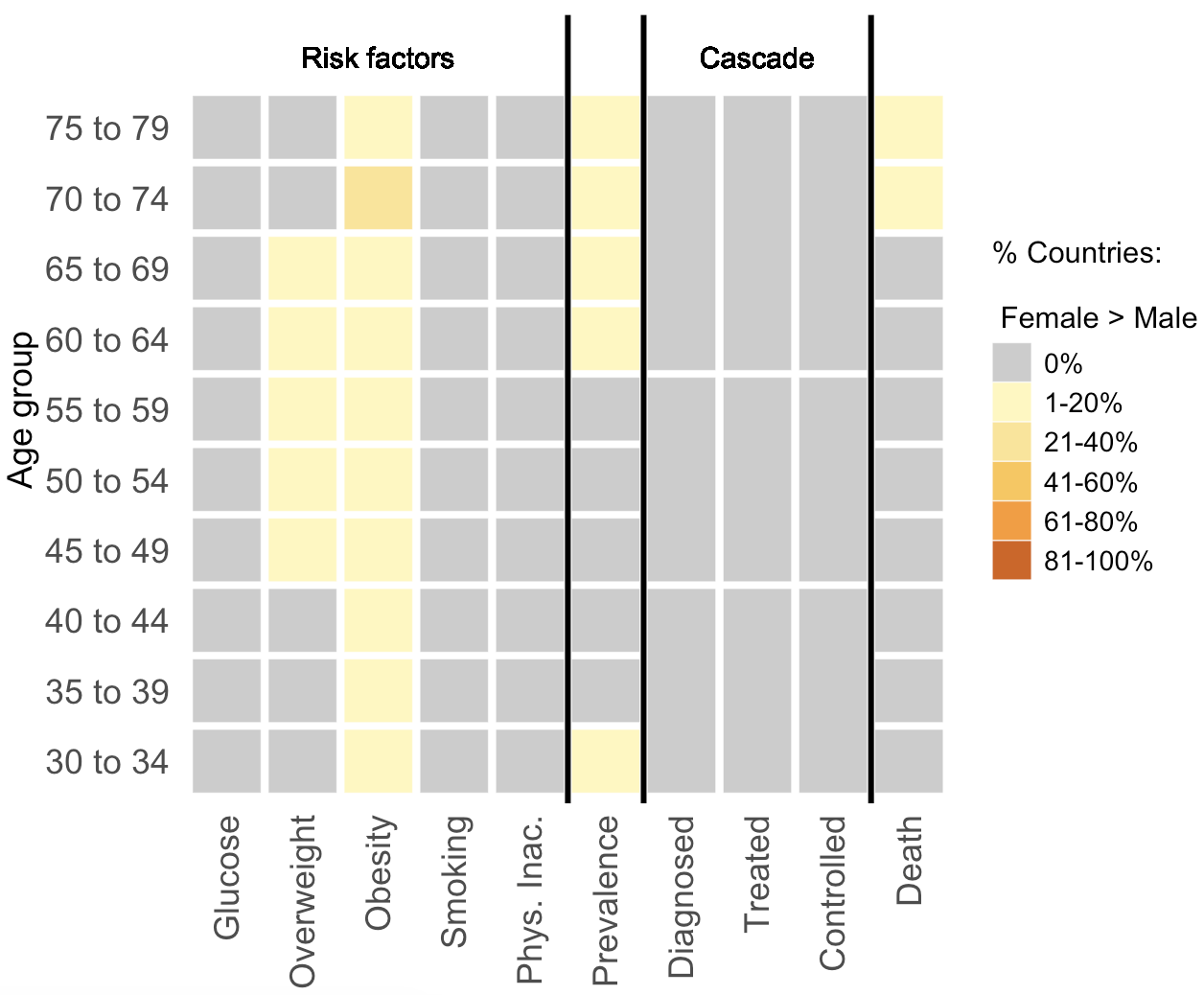 |
| * Risk factors, prevalence, death: 67 countries. Cascade: 4 countries. Phys. Inac.: Physical Inactivity. | |
| Upper-middle-income countries* | |
| 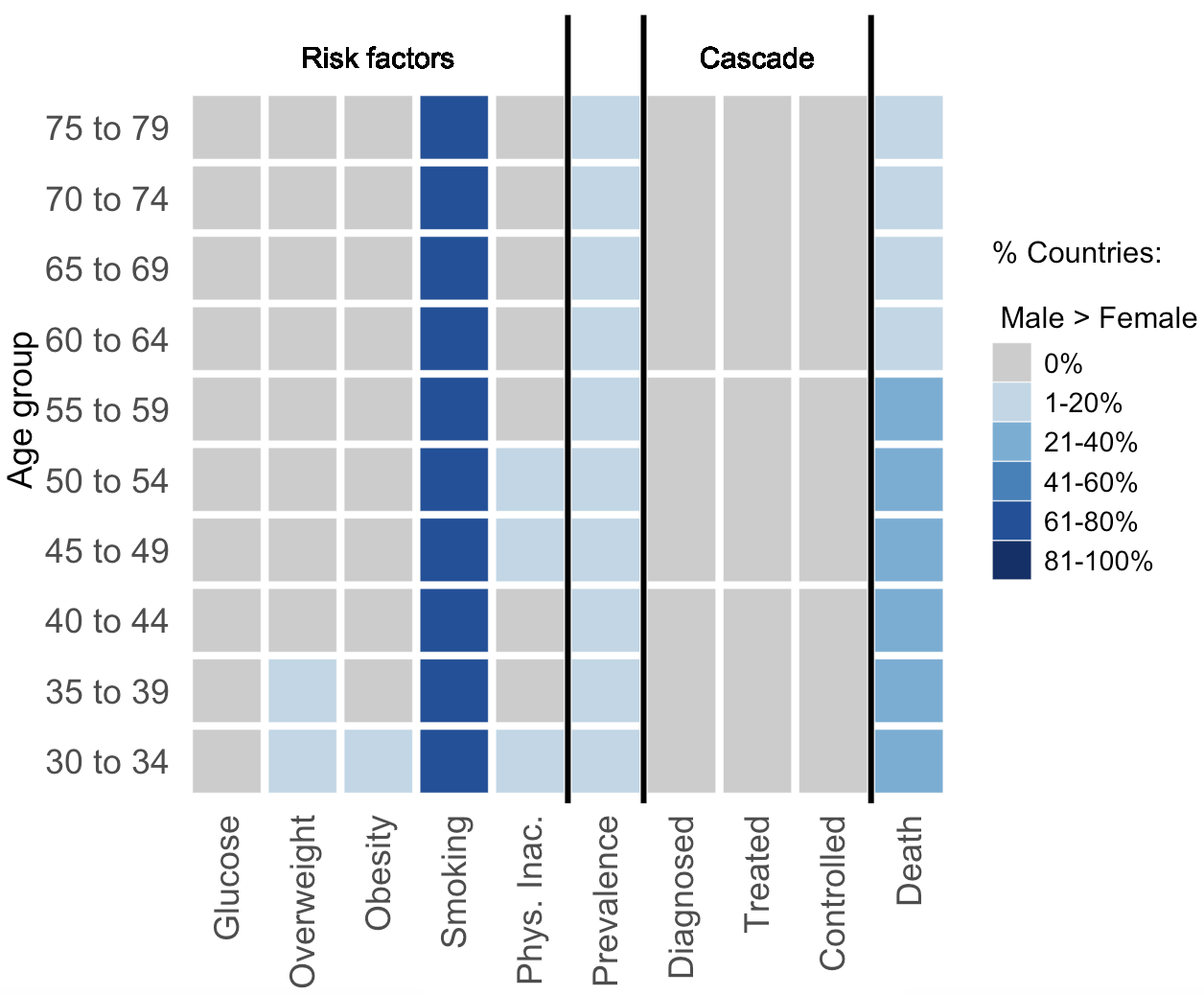 | 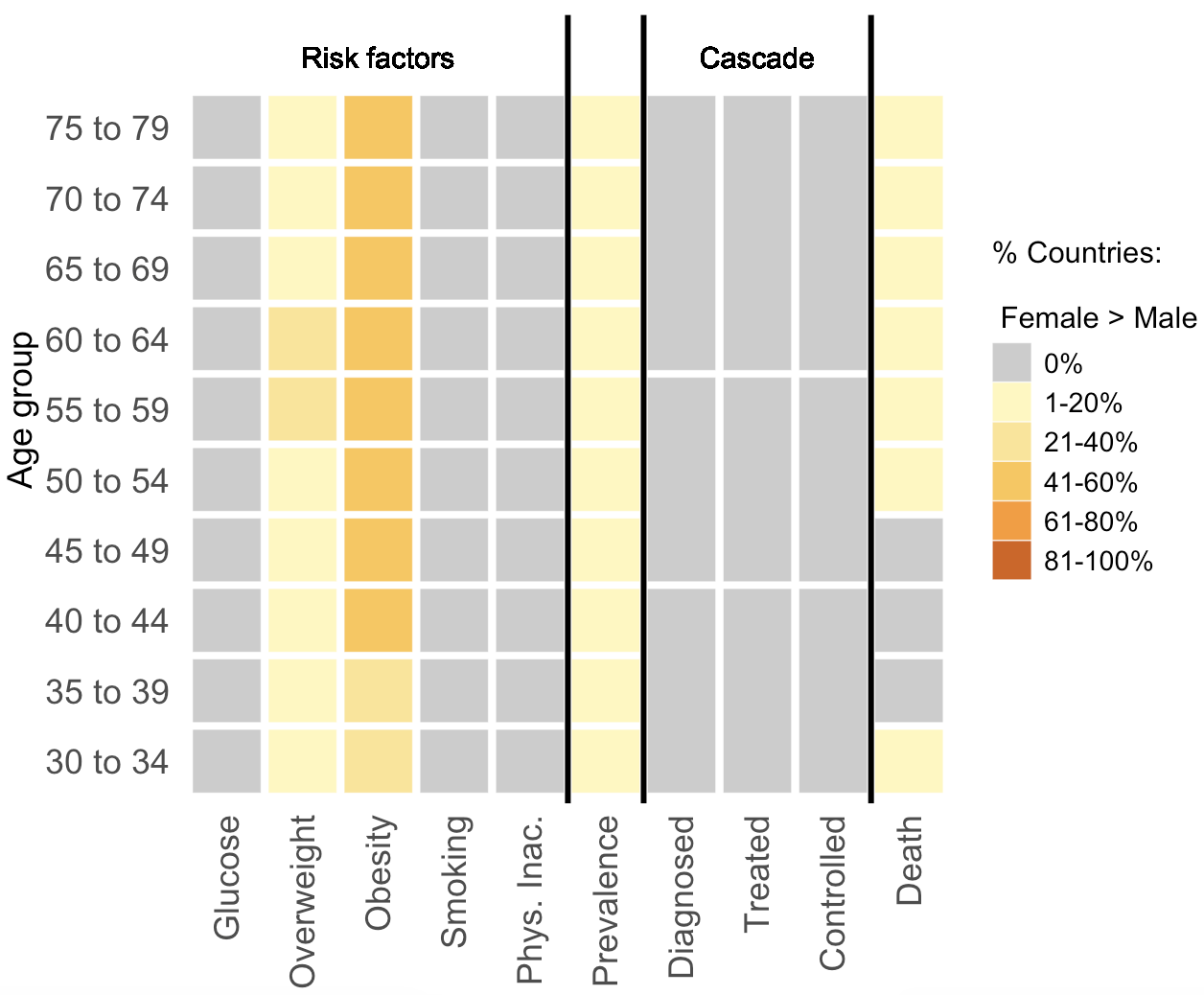 |
| * Risk factors, prevalence, death: 54 countries. Cascade: 10 countries. Phys. Inac.: Physical Inactivity. | |
| Lower-middle-income countries* | |
| 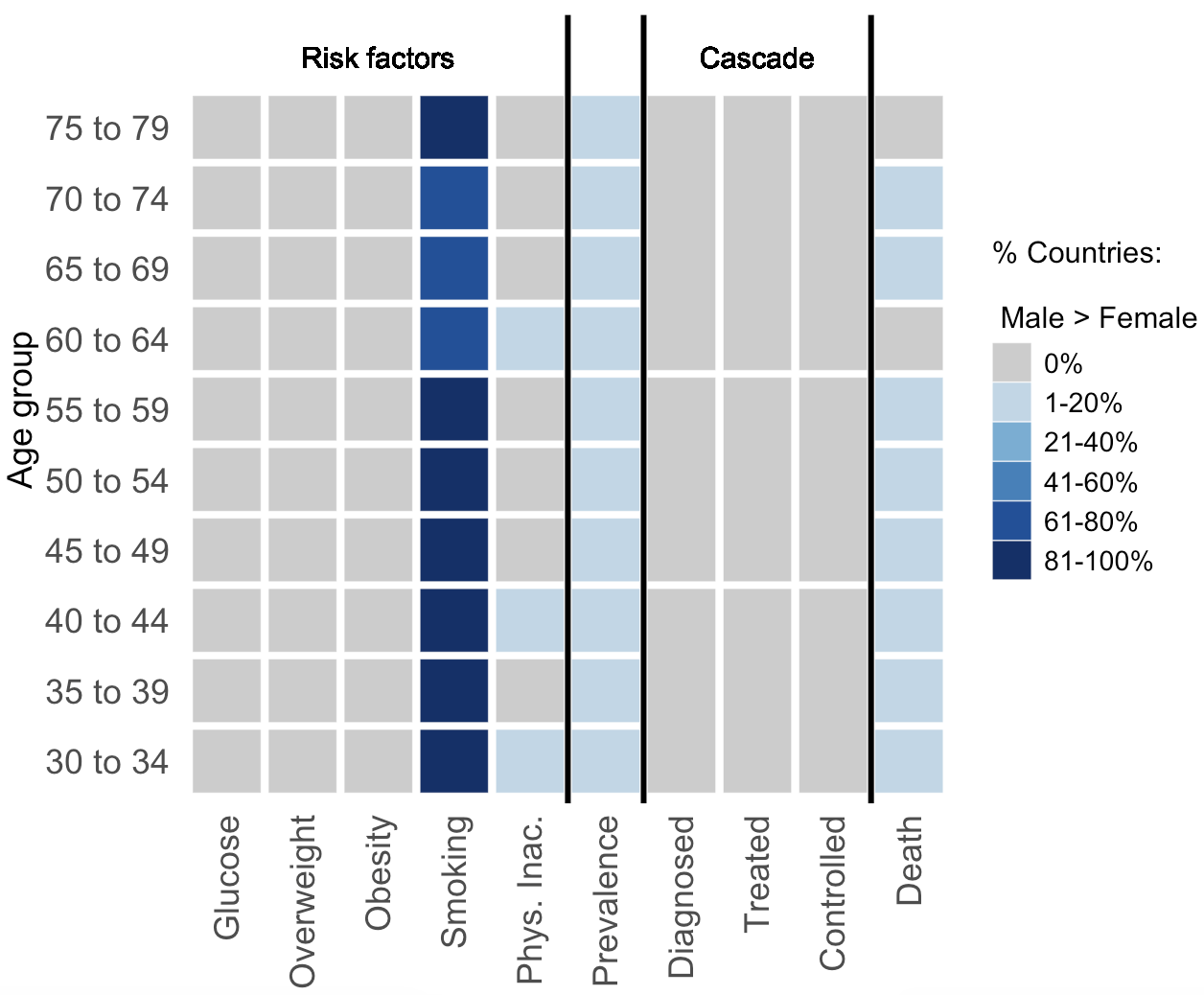 | 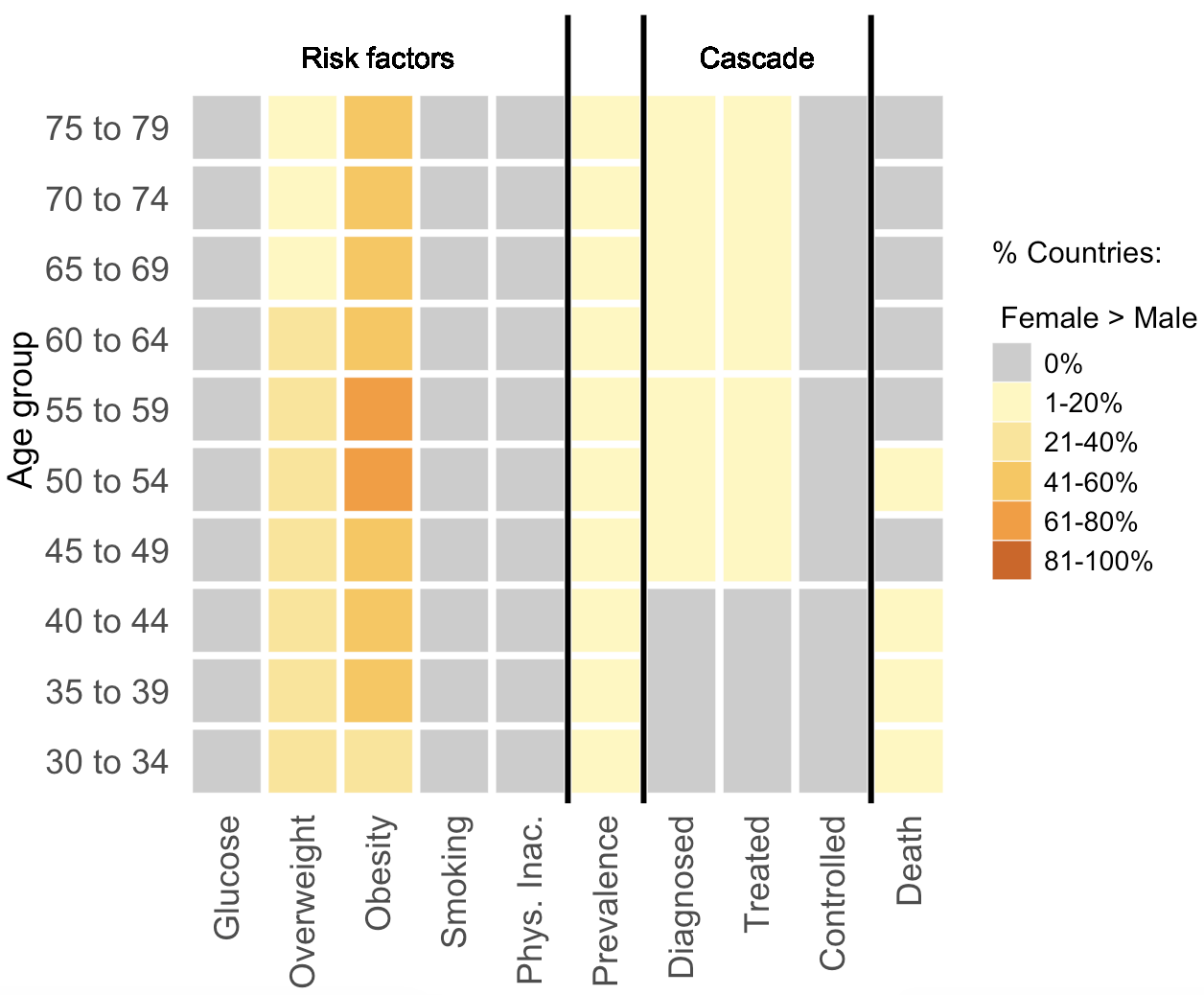 |
| * Risk factors, prevalence, death: 54 countries. Cascade: 20 countries. Phys. Inac.: Physical Inactivity. | |
| Low-income countries* | |
| 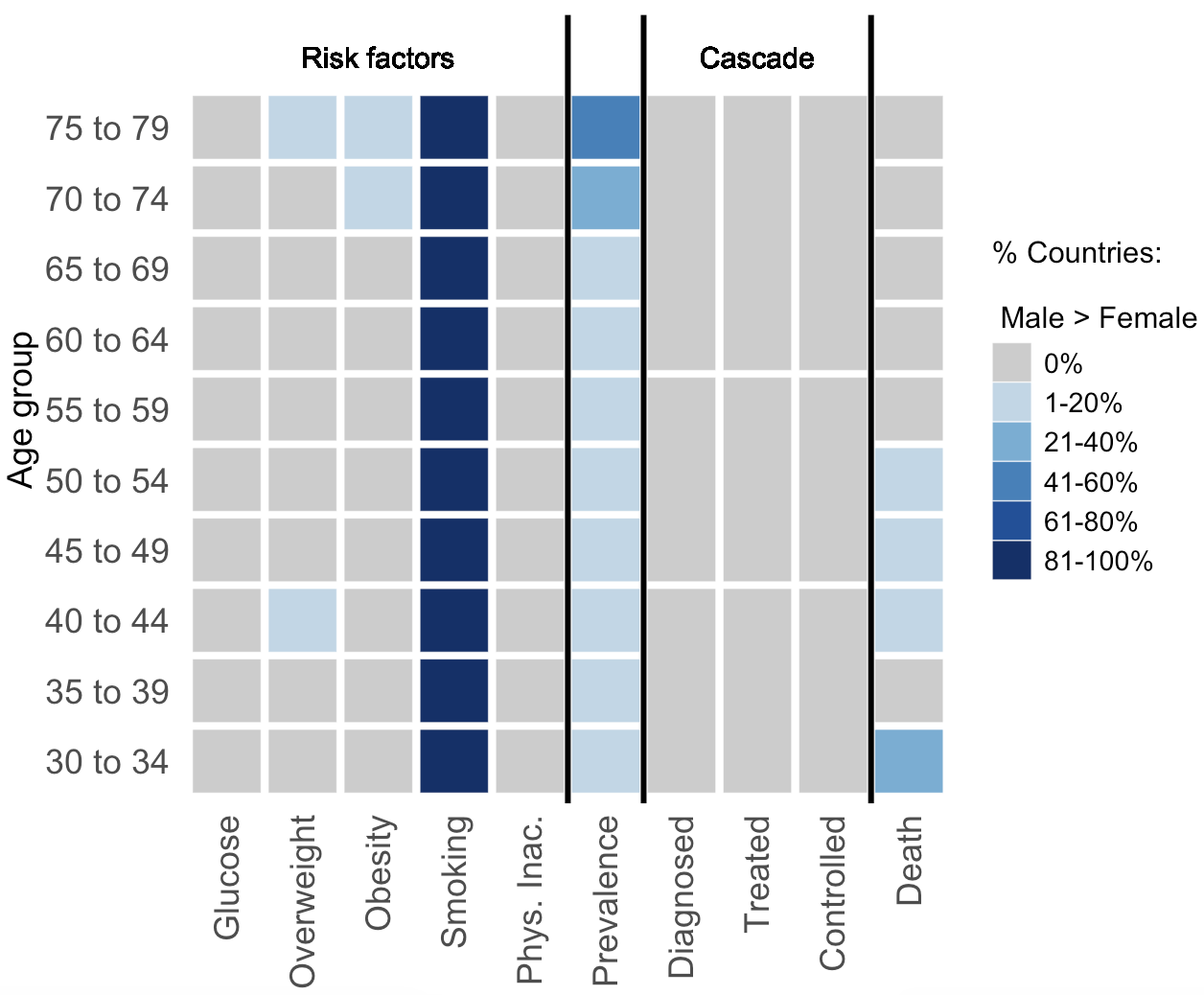 | 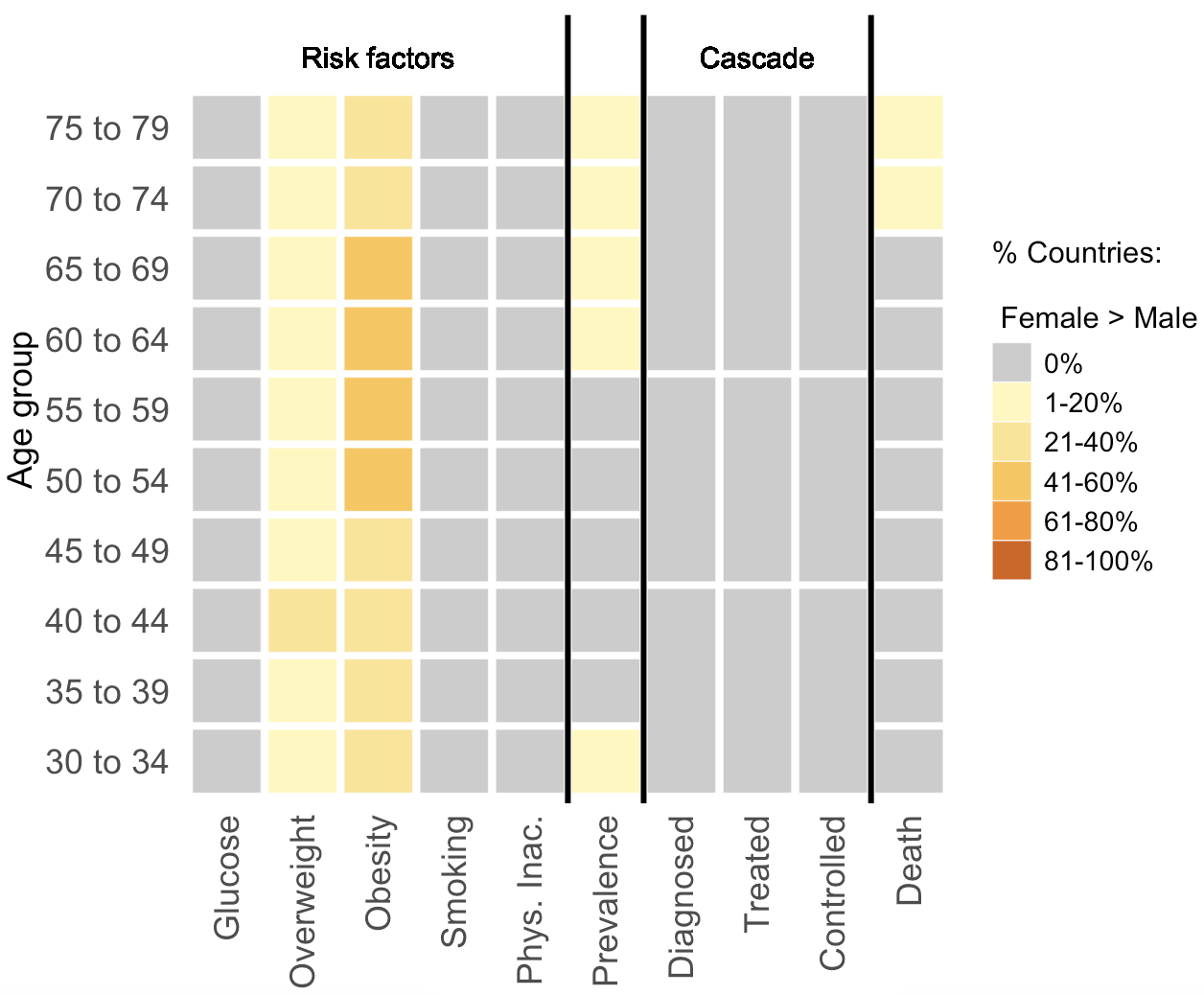 |
| * Risk factors, prevalence, death: 26 countries. Cascade: 5 countries. Phys. Inac.: Physical Inactivity. | |

**Fig K.** Percentages of countries with significant sex differences in health pathways of diabetes, by region (significant when non-overlapping confidence intervals of estimates between females and males).

| Europe and Central Asia* | |
| --- | --- |
| 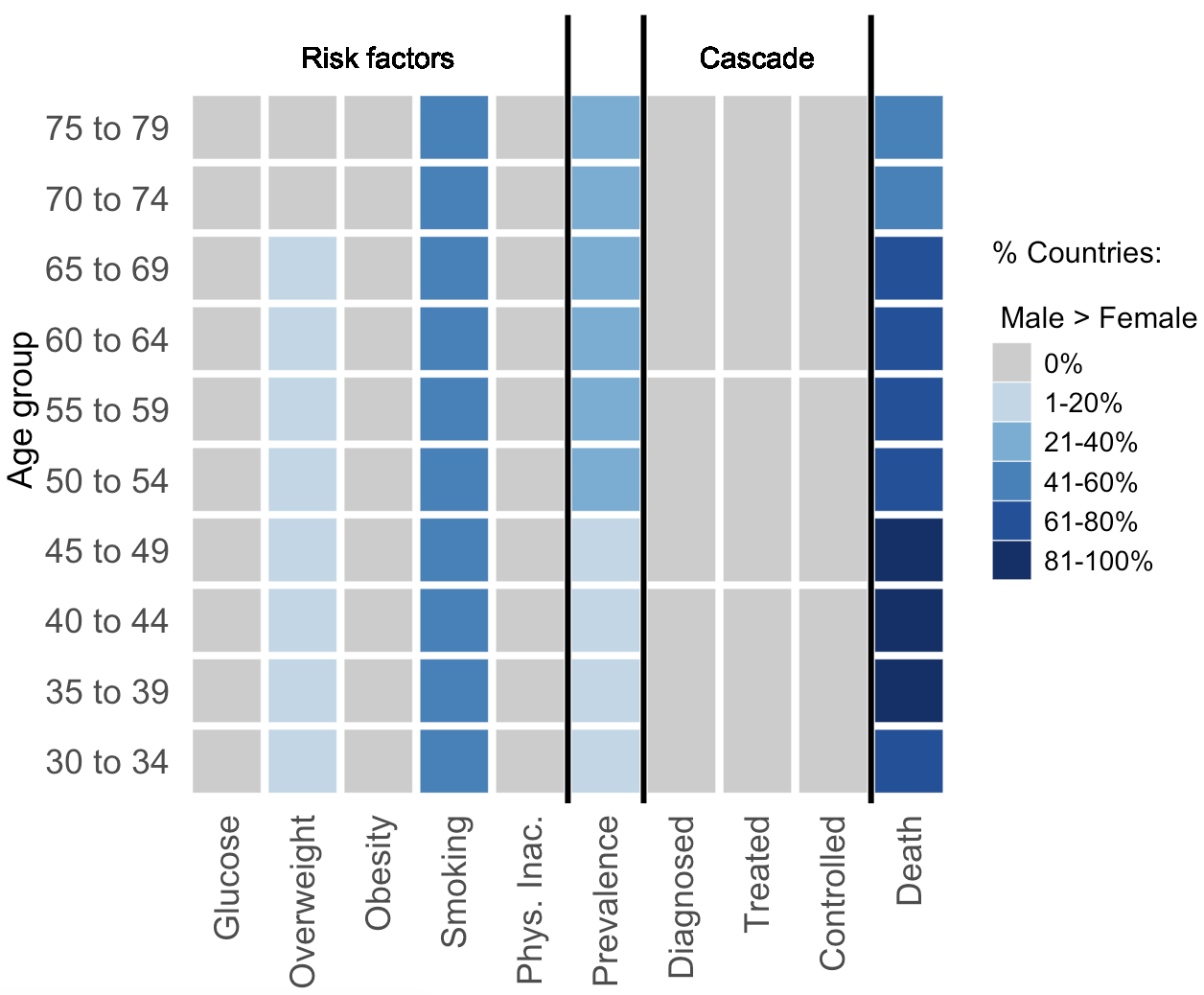 | 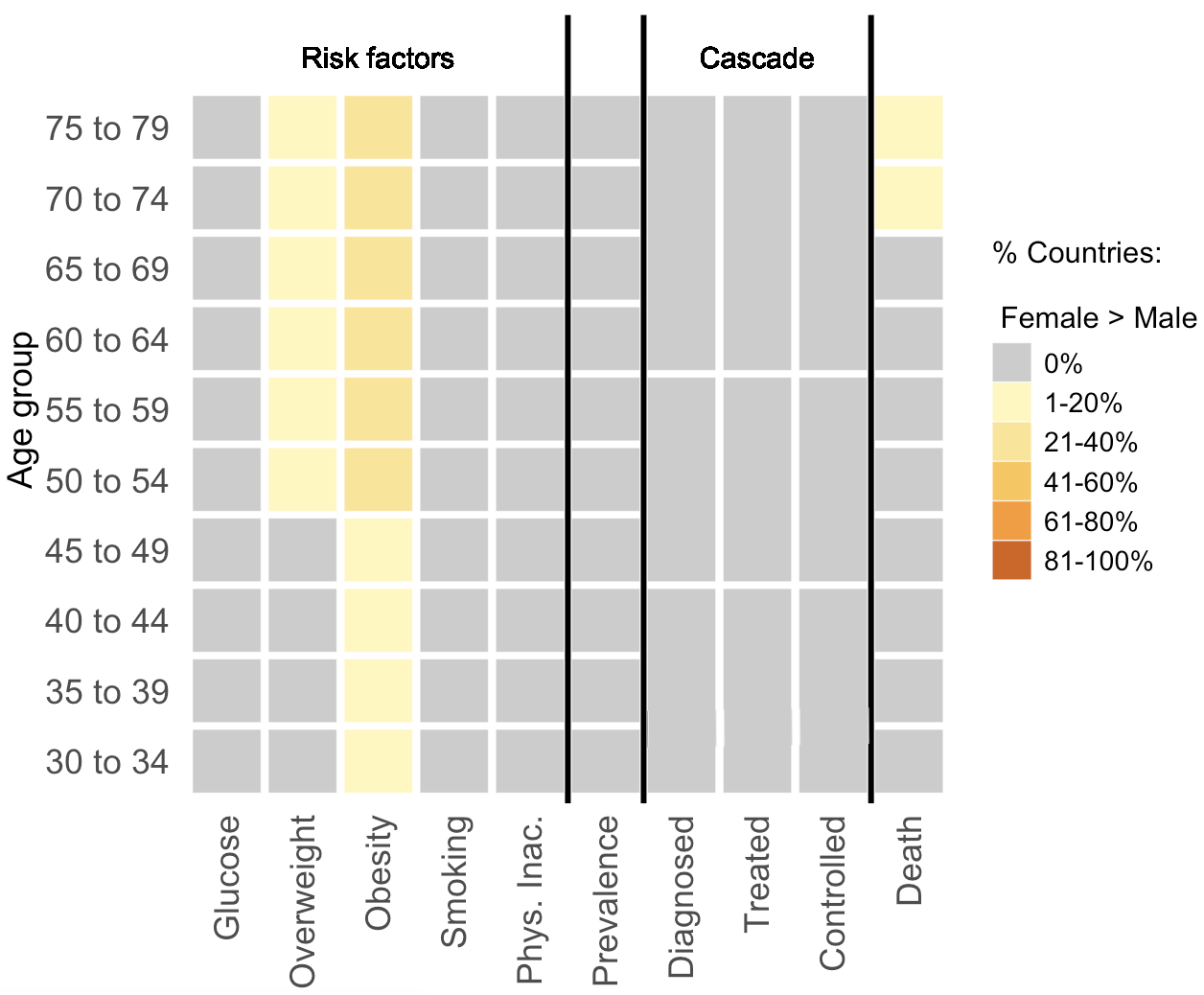 |
| * Risk factors, prevalence, death: 52 countries. Cascade: 8 countries. Phys. Inac.: Physical Inactivity. | |
| South Asia* | |
| 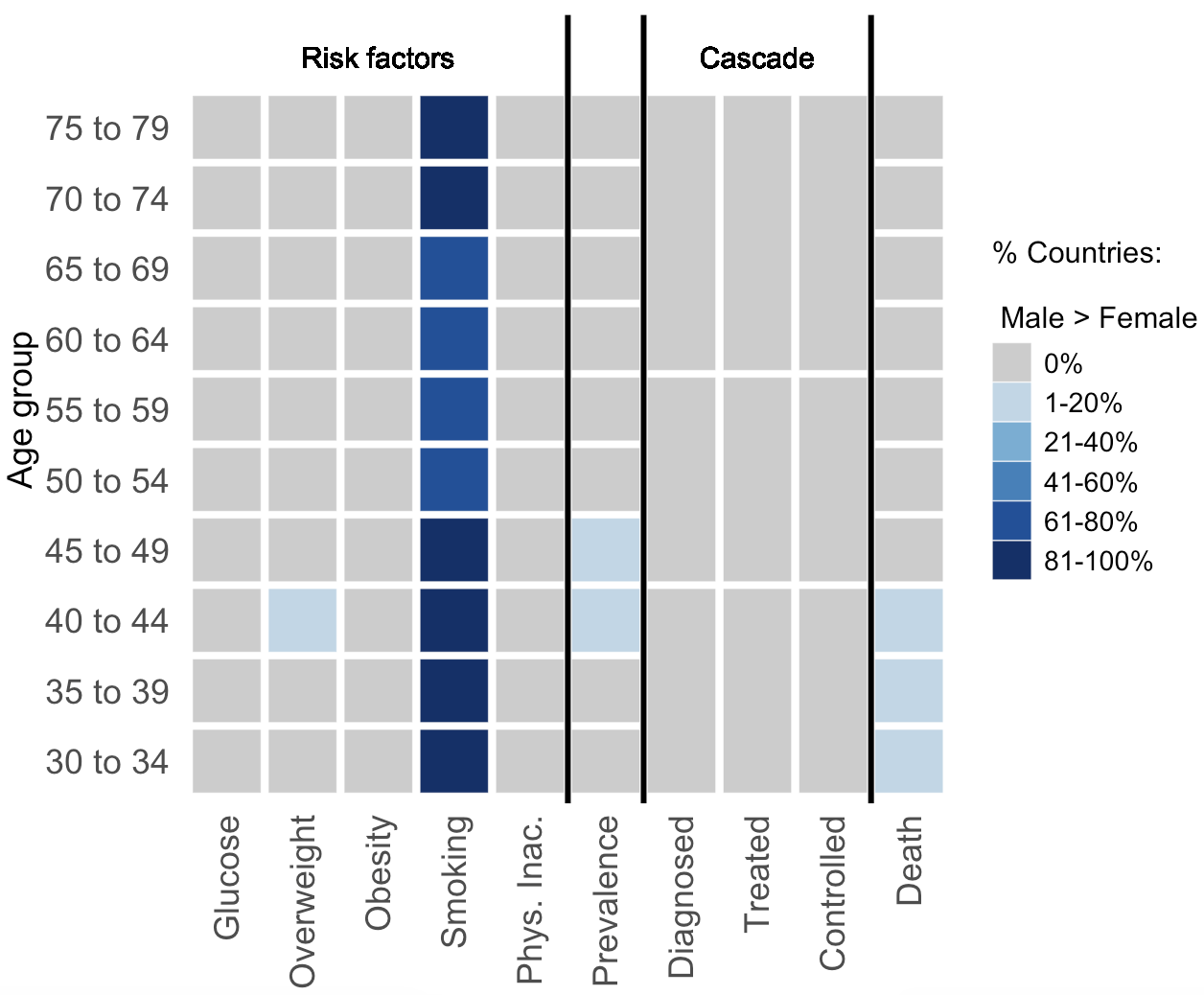 | 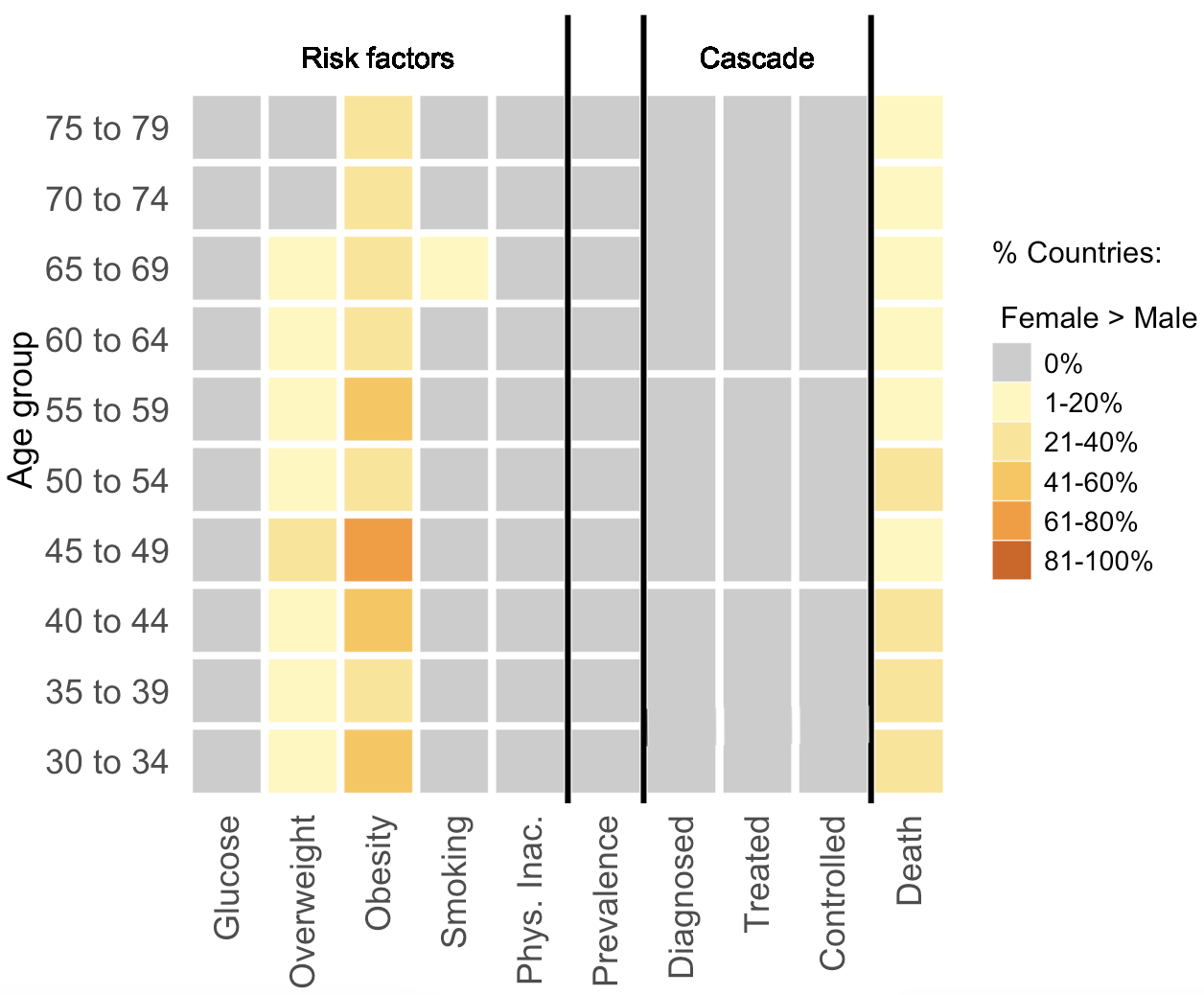 |
| * Risk factors, prevalence, death: 8 countries. Cascade: 4 countries. Phys. Inac.: Physical Inactivity. | |
| Sub-Saharan Africa* | |
| 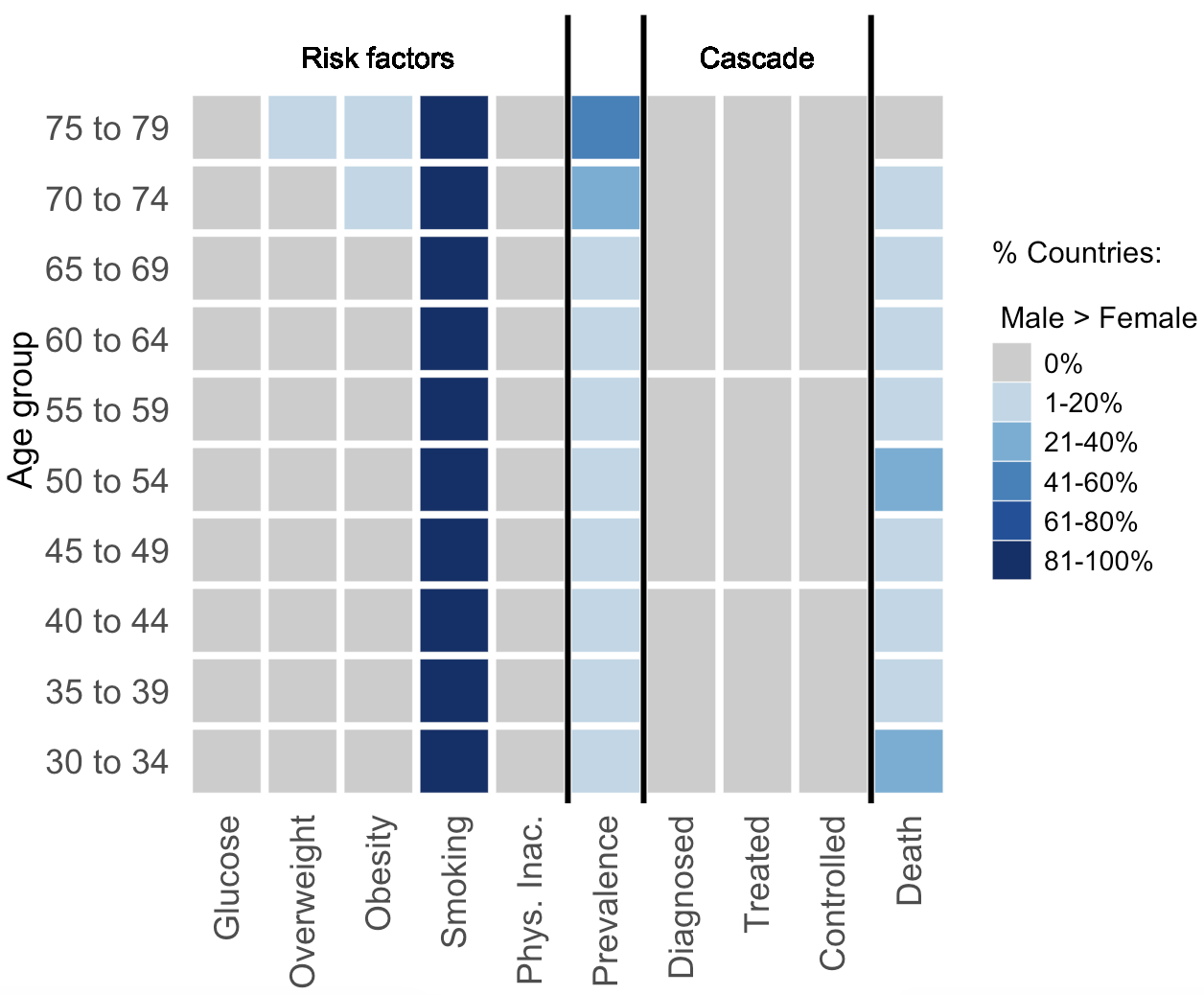 | 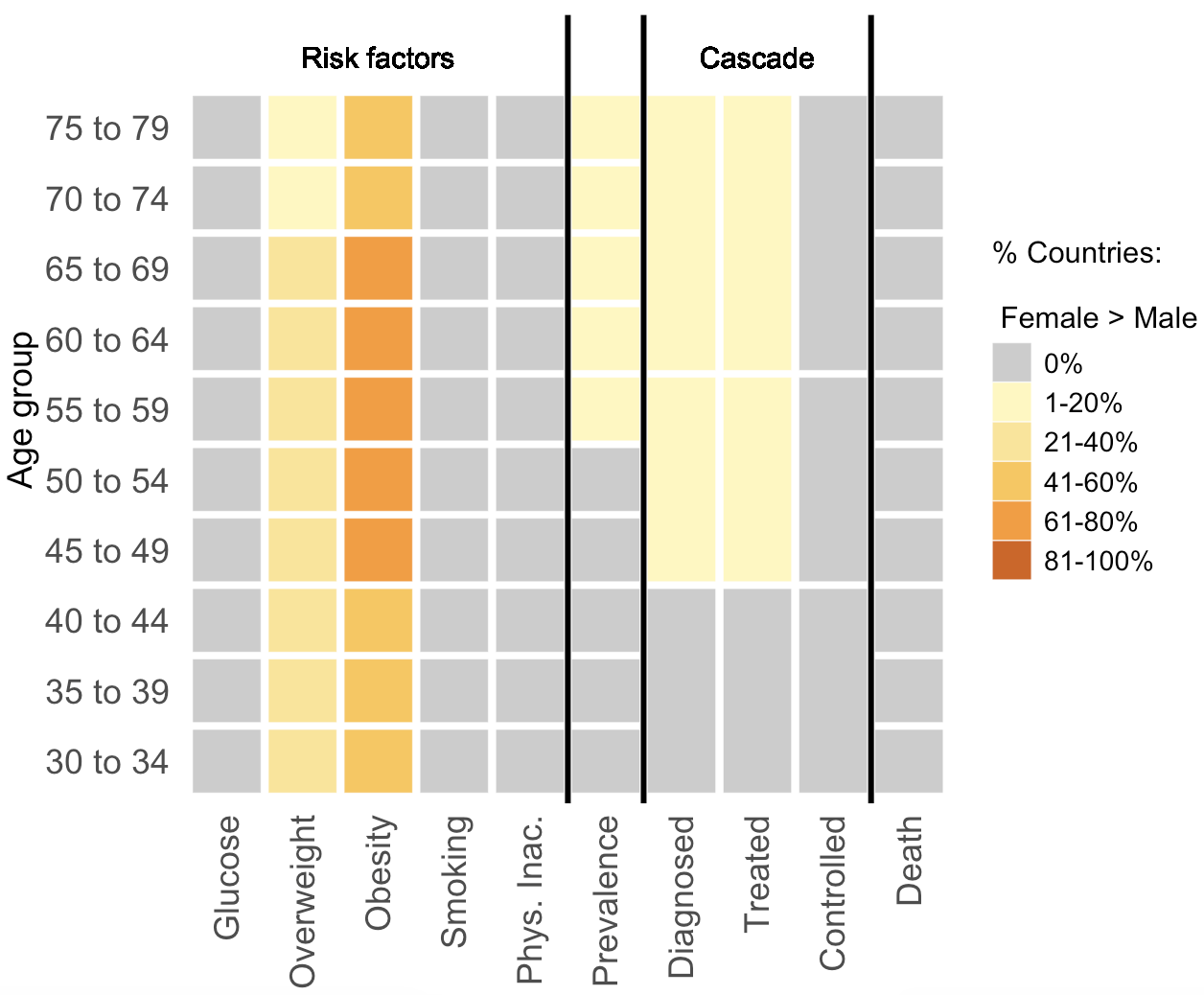 |
| * Risk factors, prevalence, death: 44 countries. Cascade: 11 countries. Phys. Inac.: Physical Inactivity. | |
| Middle East and North Africa* | |
| 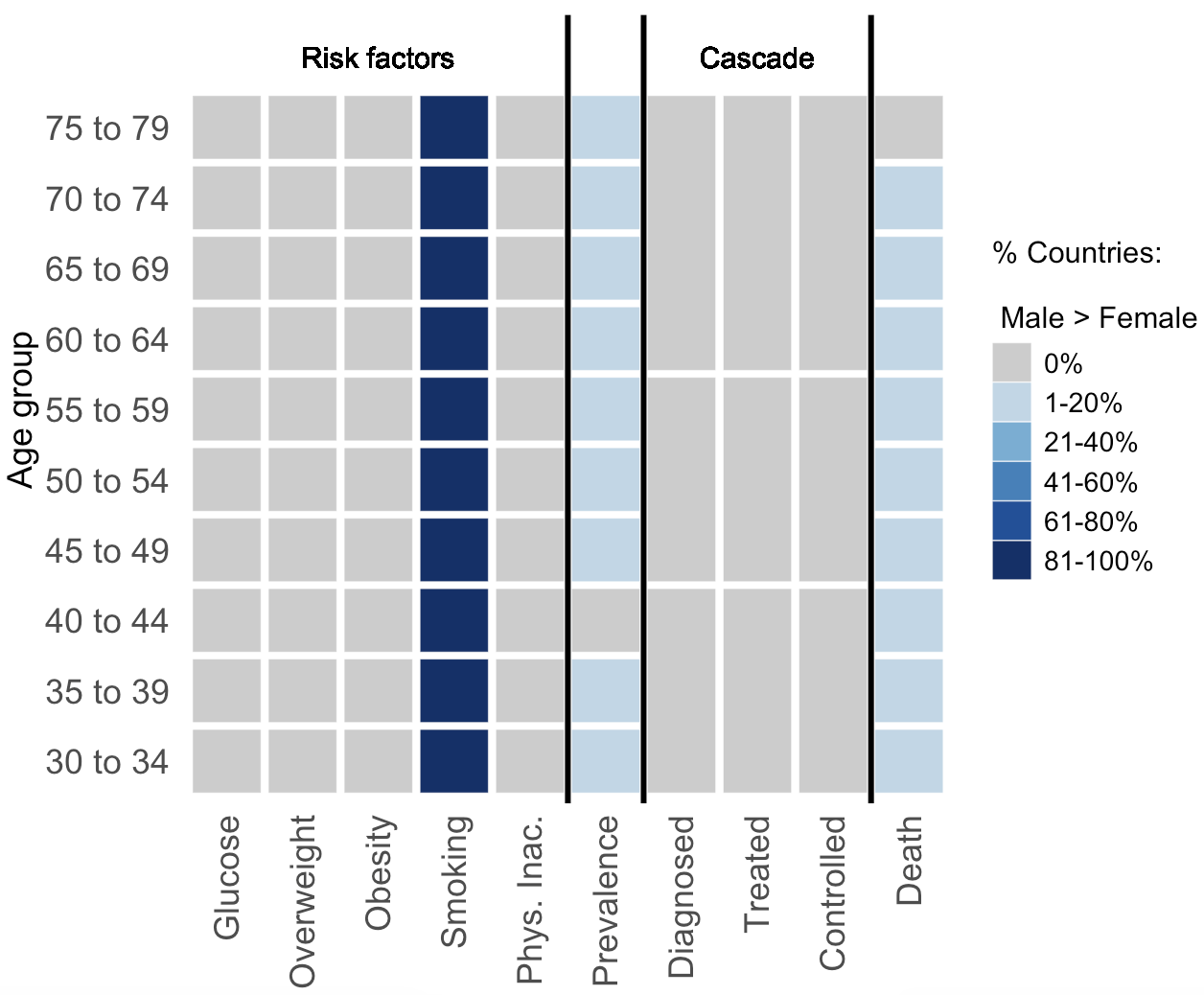 | 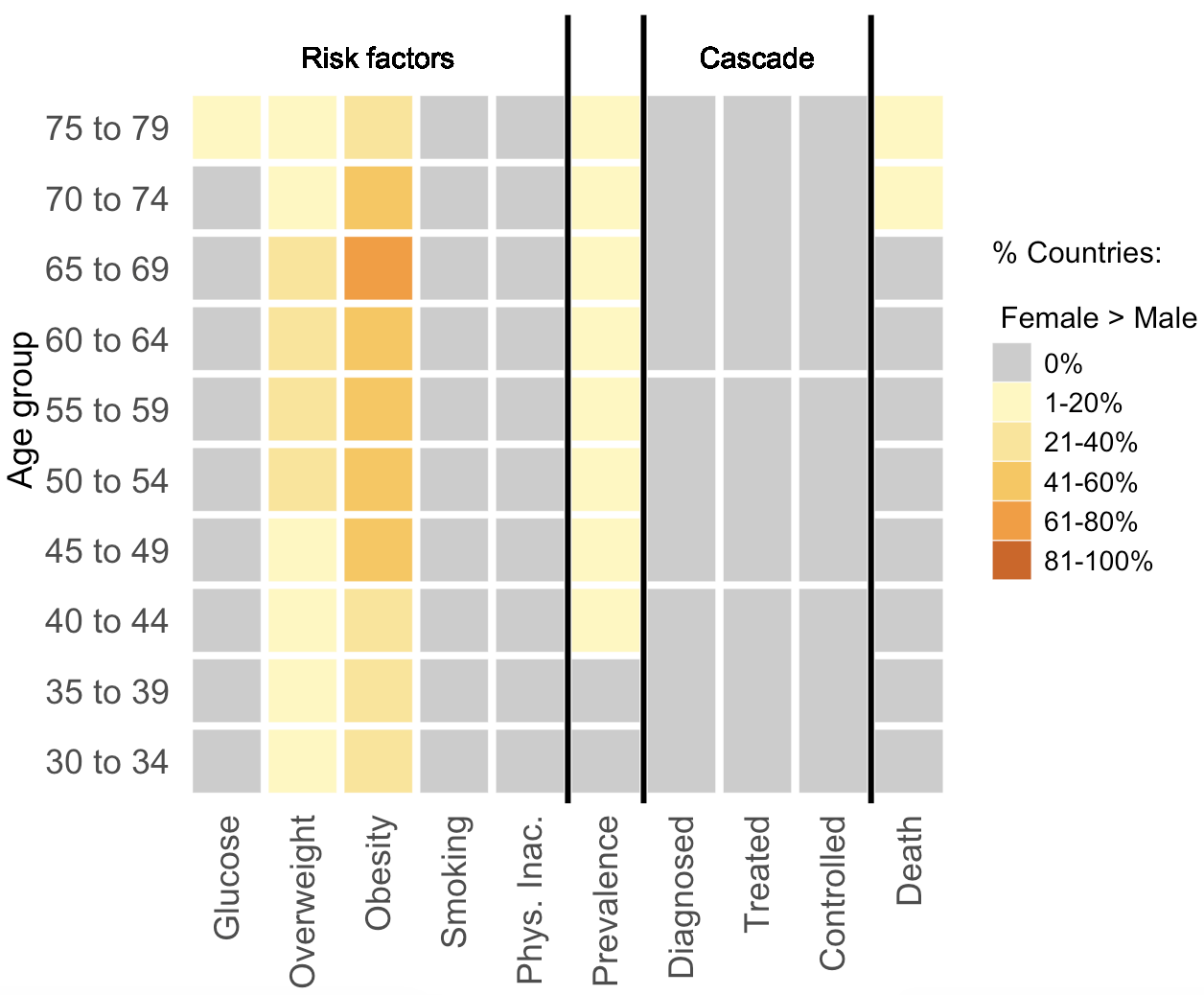 |
| * Risk factors, prevalence, death: 22 countries. Cascade: 6 countries. Phys. Inac.: Physical Inactivity. | |
| Latin America & the Caribbean* | |
| 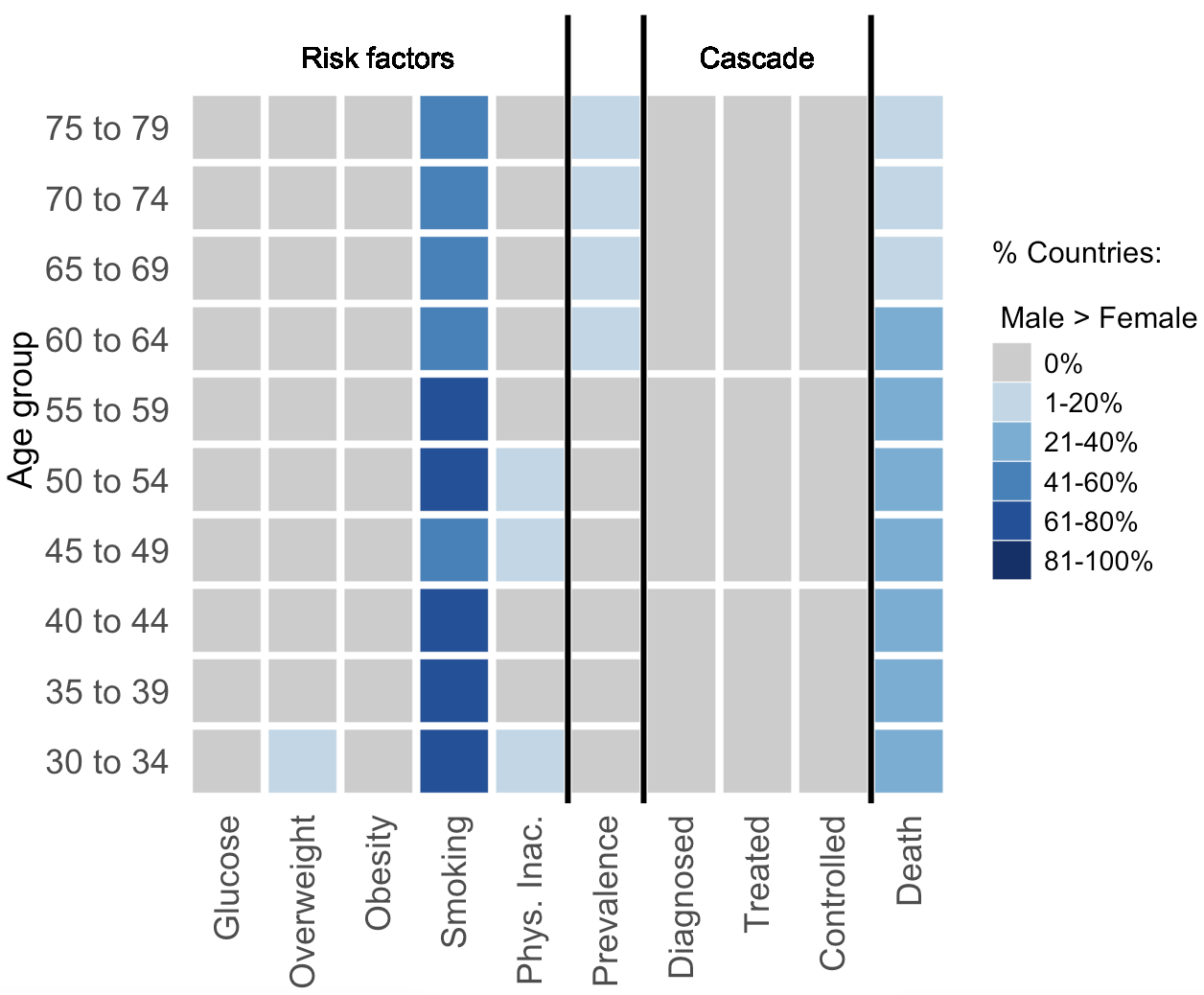 | 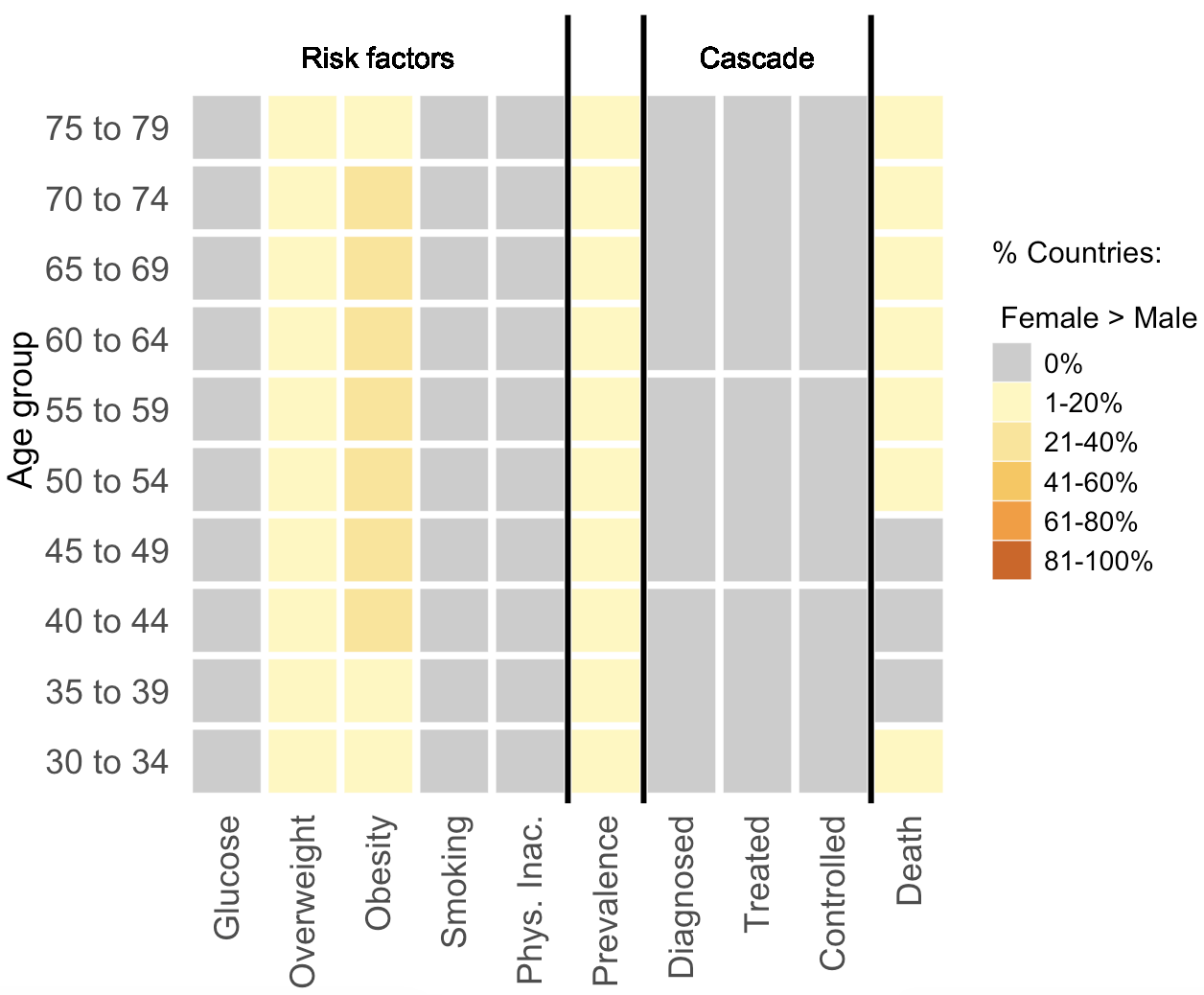 |
| * Risk factors, prevalence, death: 38 countries. Cascade: 2 countries. Phys. Inac.: Physical Inactivity. | |
| East Asia and Pacific* | |
| 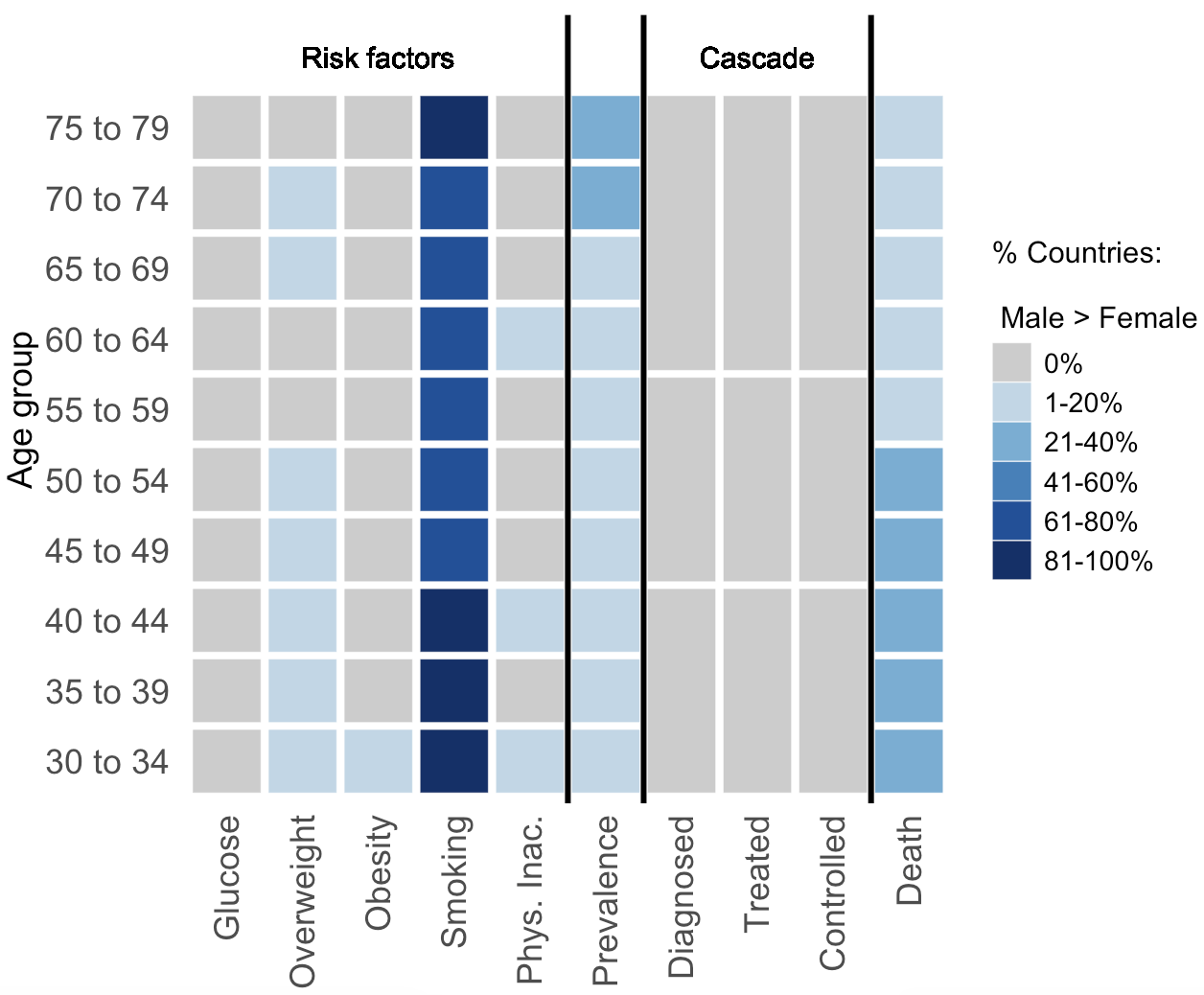 | 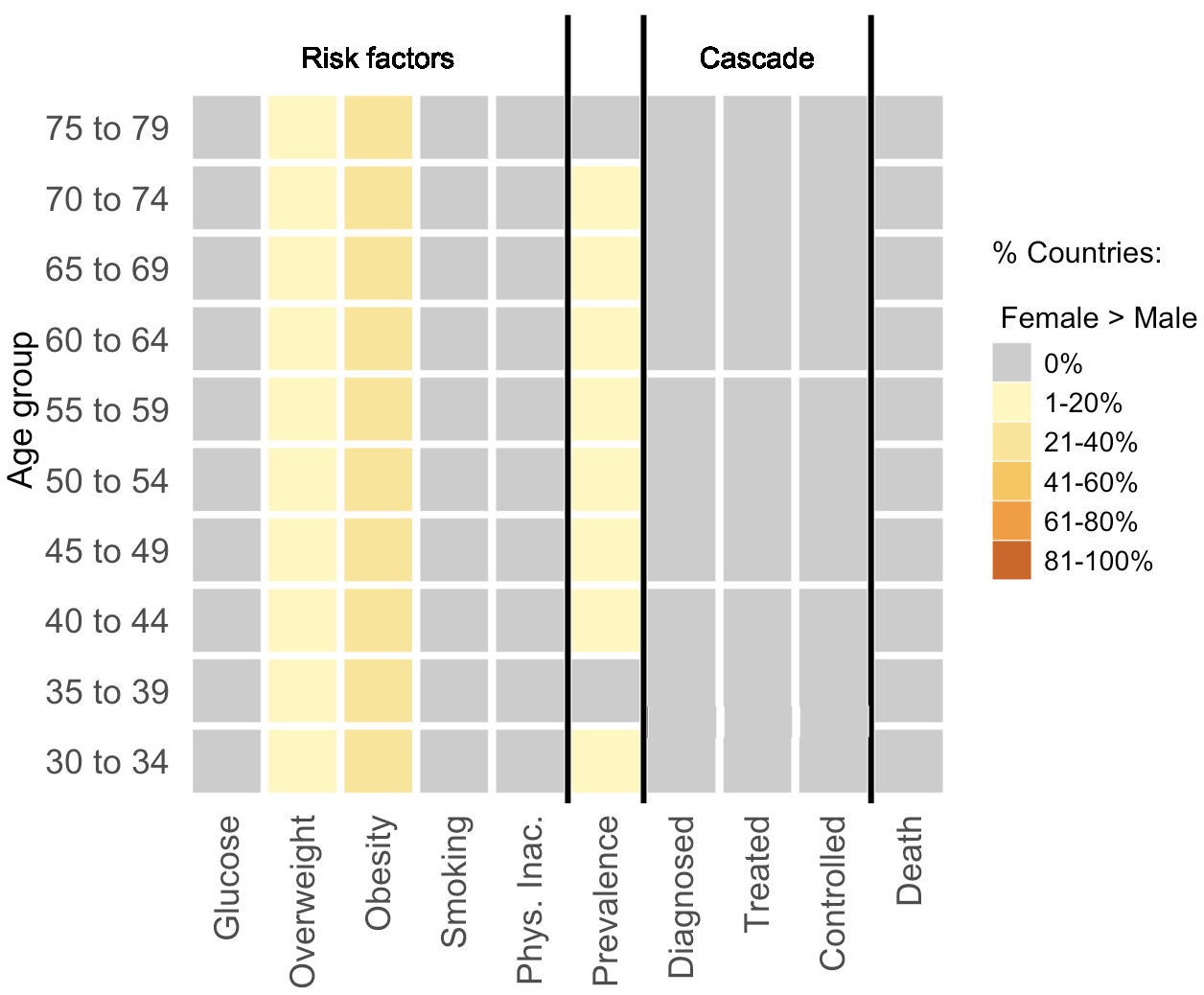 |
| * Risk factors, prevalence, death: 34 countries. Cascade: 8 countries. Phys. Inac.: Physical Inactivity. | |
| North America* | |
| 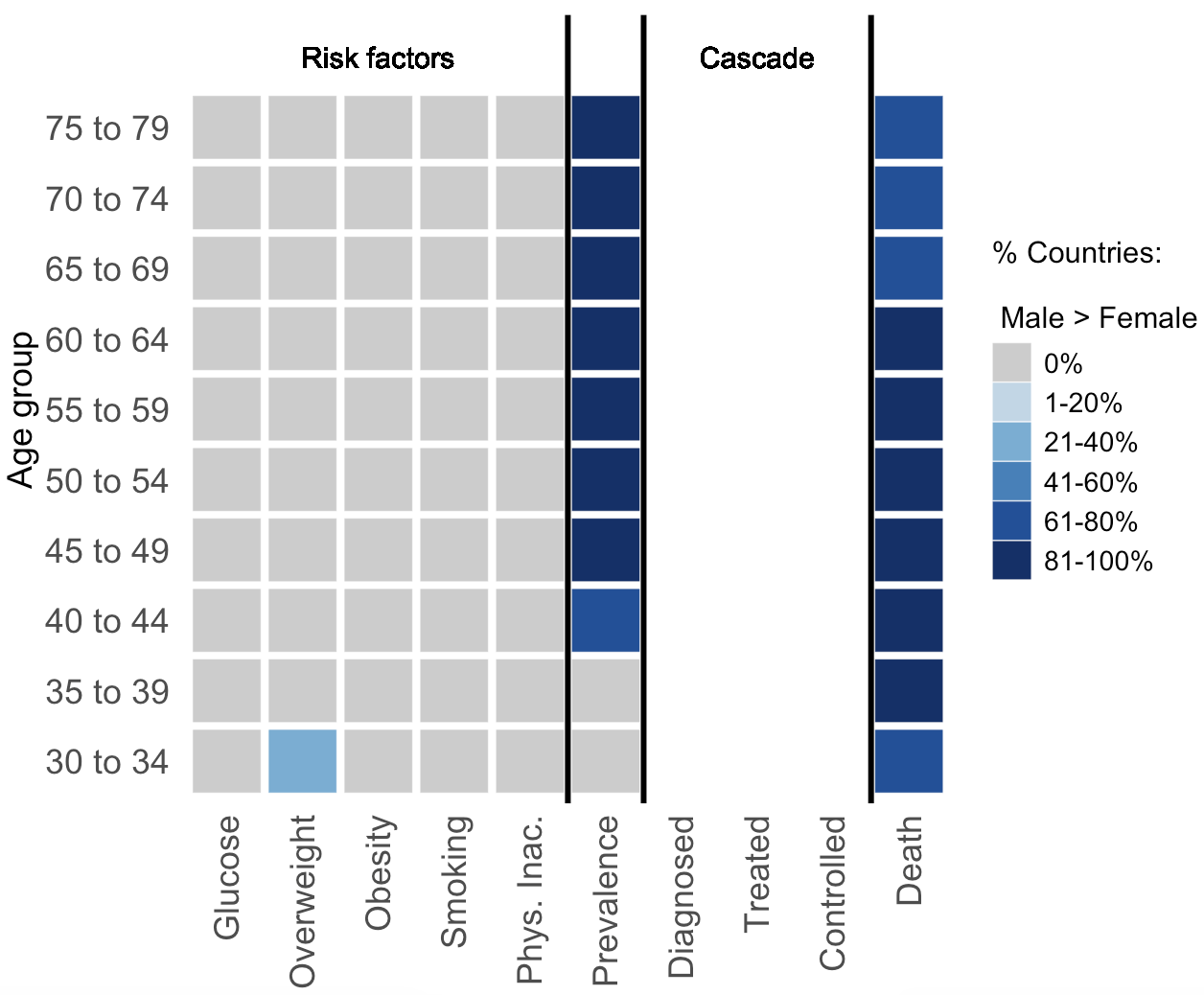 | 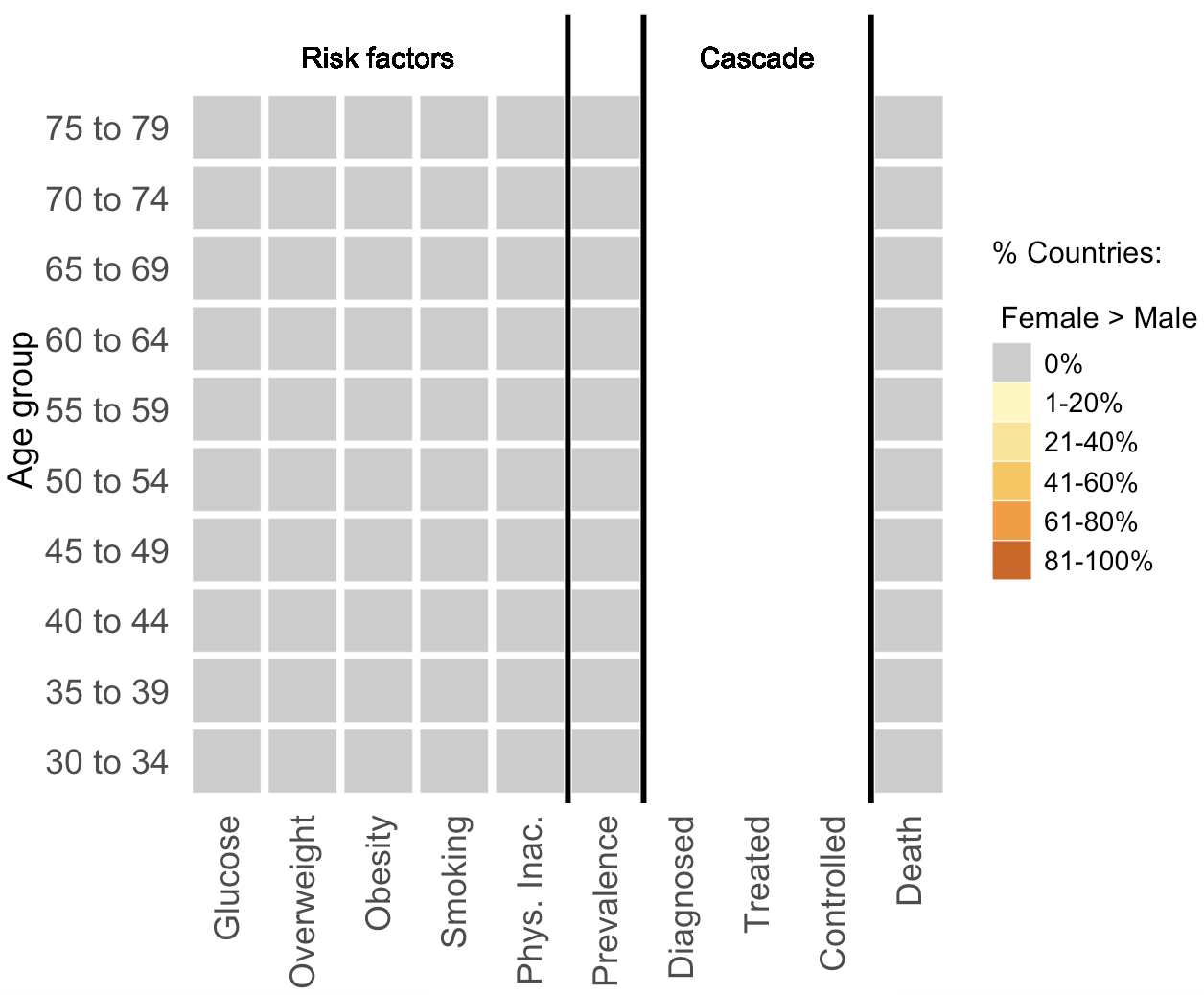 |
| * Risk factors, prevalence, death: 3 countries. Cascade: No countries. Phys. Inac.: Physical Inactivity. | |

**Fig L.** Percentages of countries with significant sex differences in health pathways of HIV and AIDS, by income group (significant when non-overlapping confidence intervals of estimates between females and males).

| High-income countries* | |
| --- | --- |
| 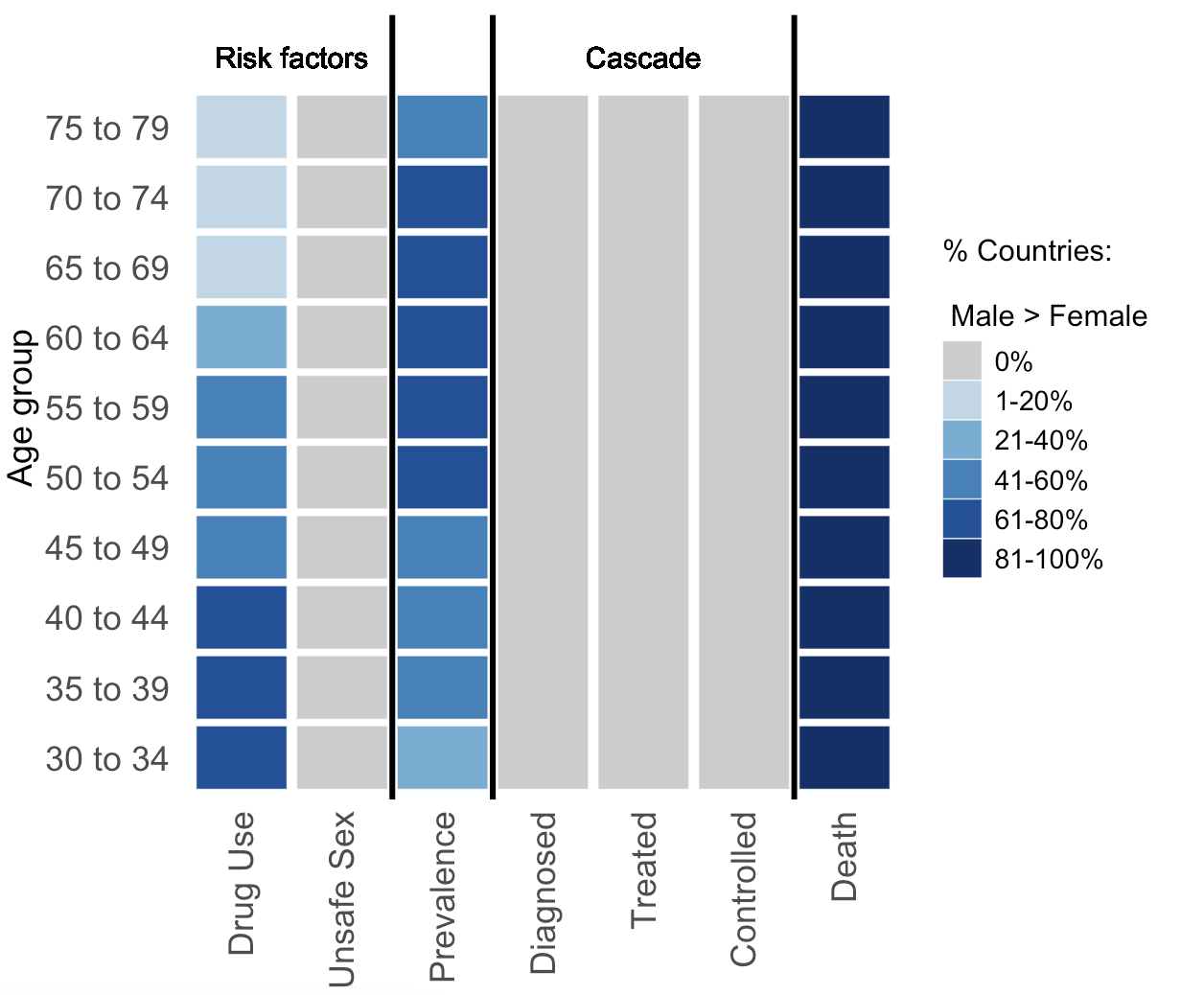 | 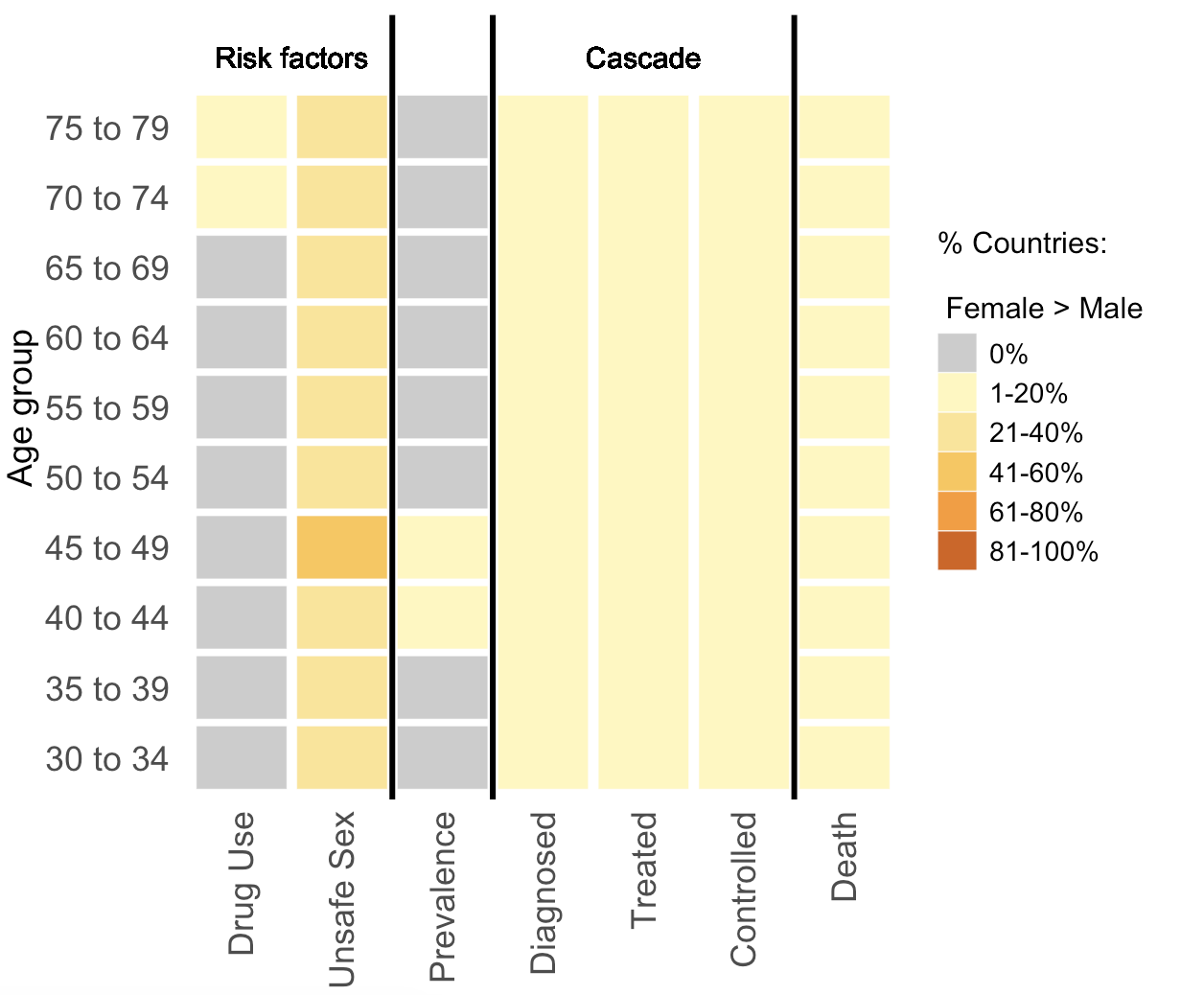 |
| * Risk factors, prevalence, death: 67 countries. Cascade: 16 countries. Phys. Inac.: Physical Inactivity. | |
| Upper-middle-income countries* | |
| 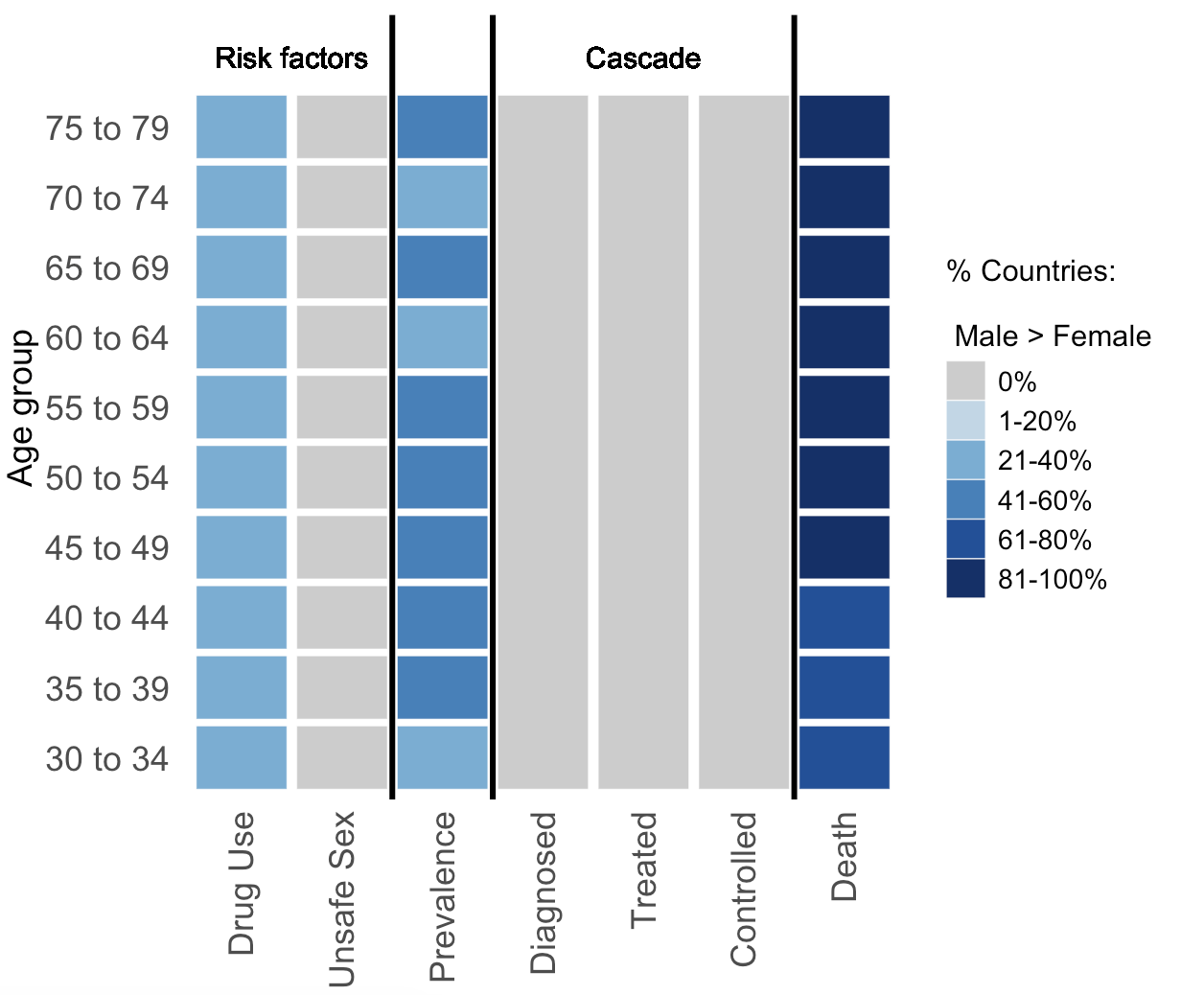 | 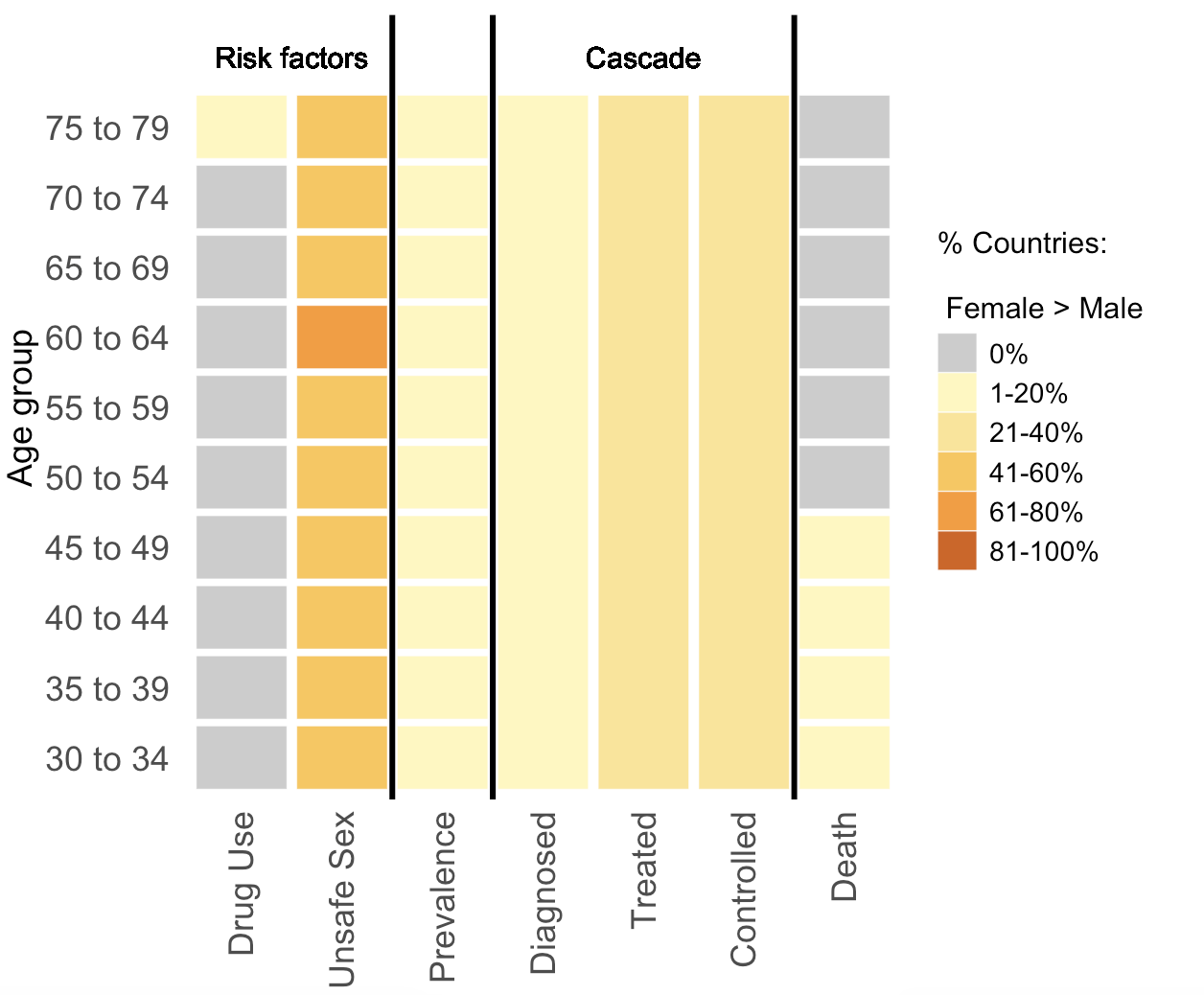 |
| * Risk factors, prevalence, death: 54 countries. Cascade: 25 countries. Phys. Inac.: Physical Inactivity. | |
| Lower-middle-income countries* | |
| 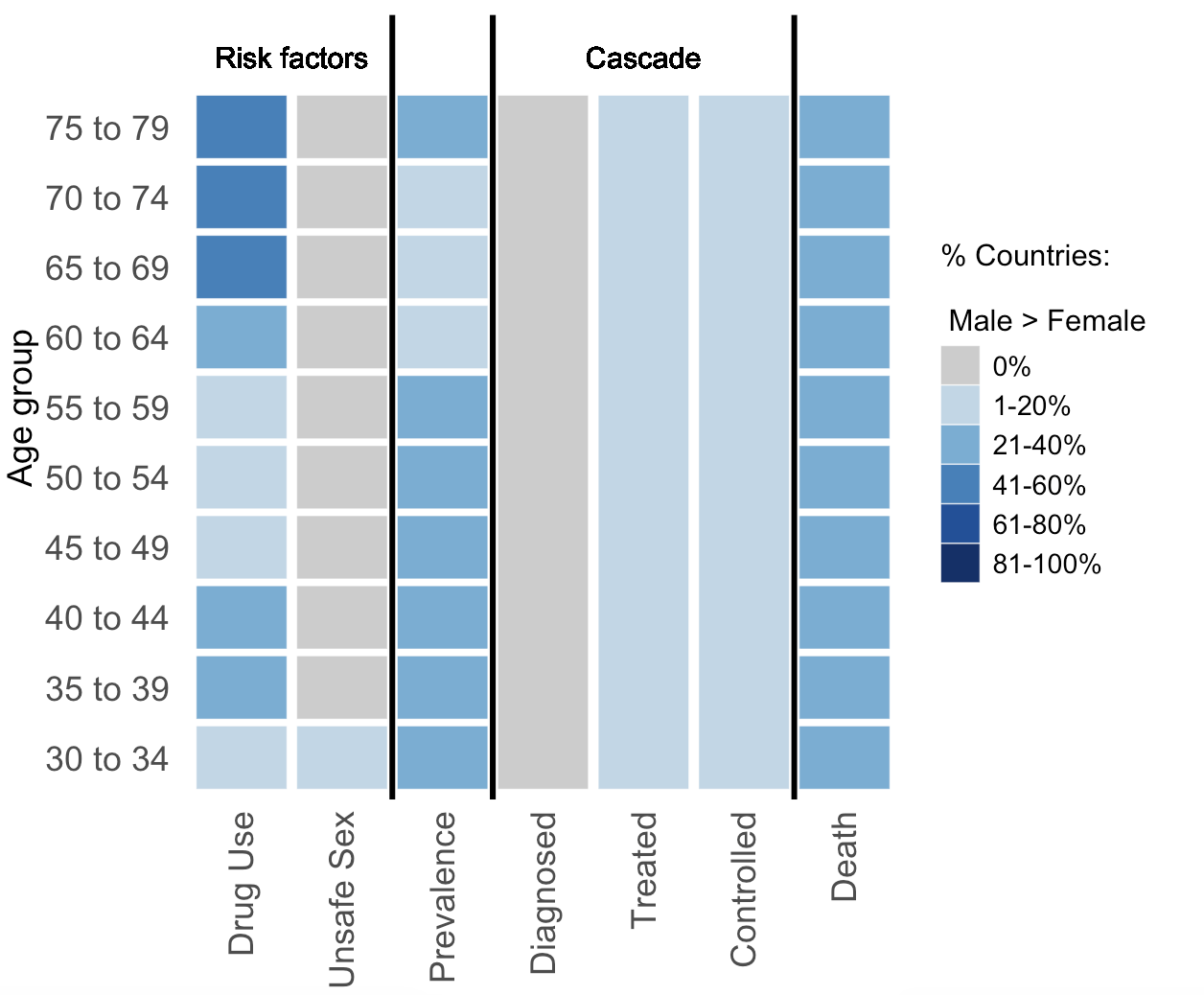 | 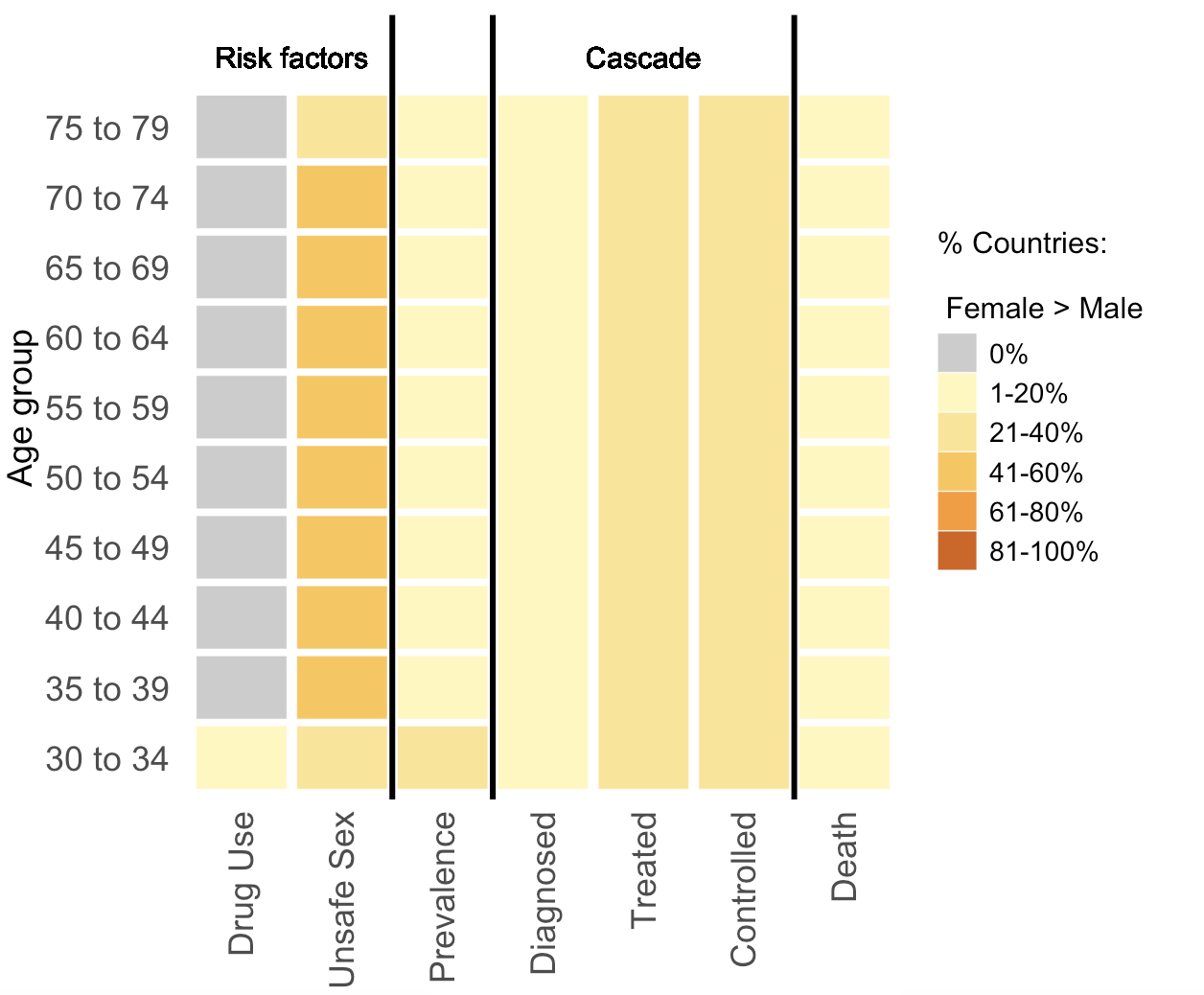 |
| * Risk factors, prevalence, death: 54 countries. Cascade: 28 countries. Phys. Inac.: Physical Inactivity. | |
| Low-income countries* | |
| 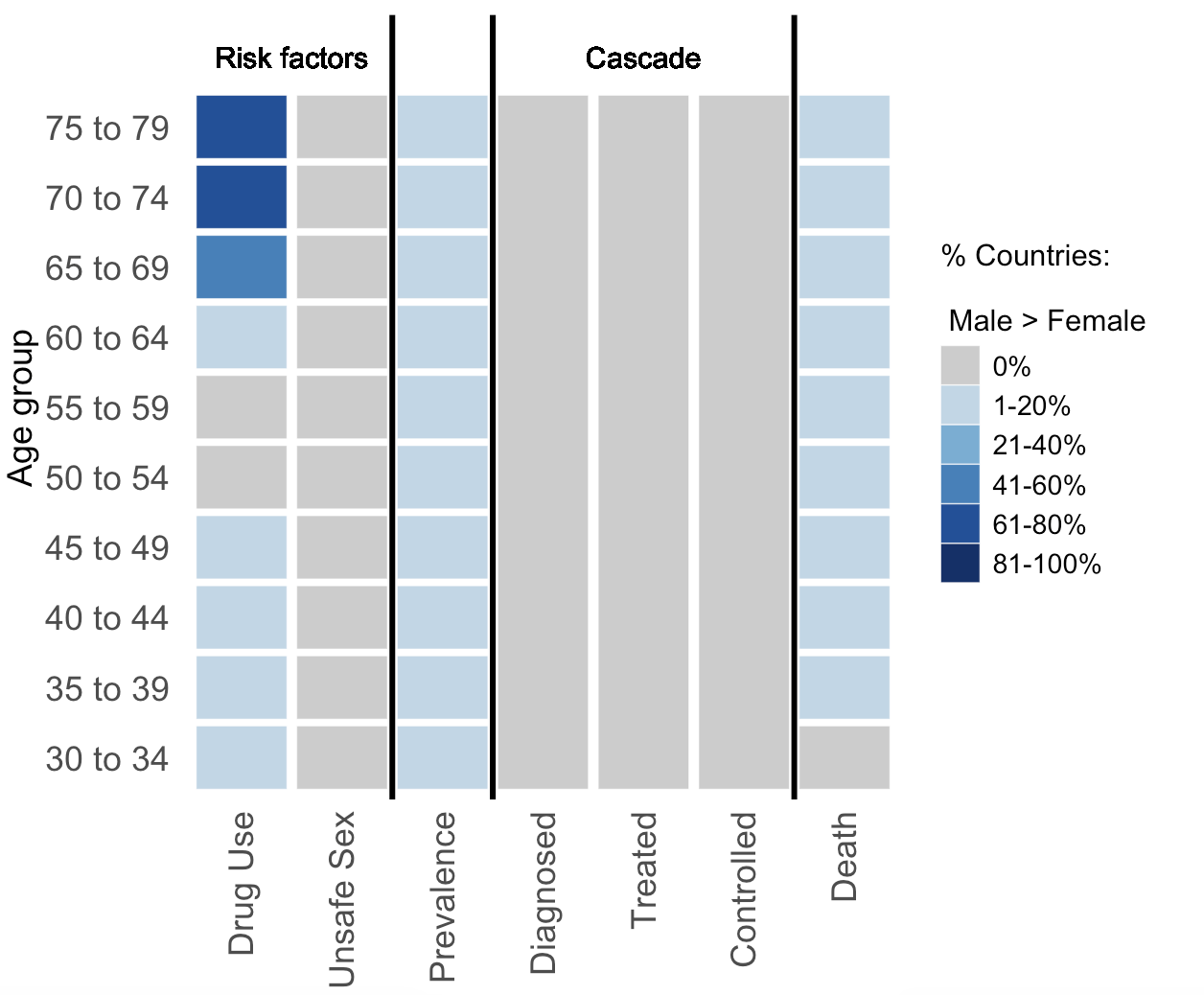 | 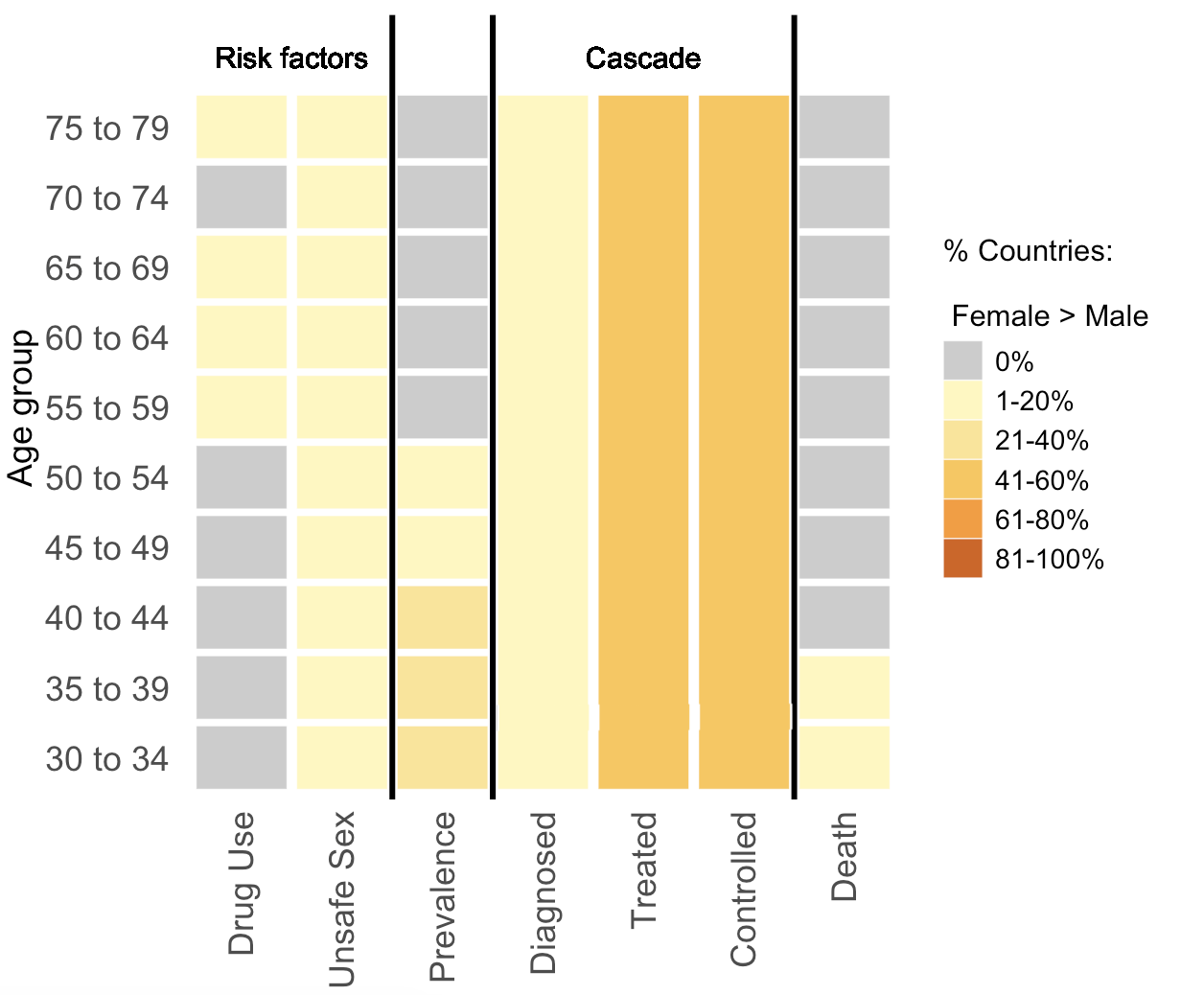 |
| * Risk factors, prevalence, death: 26 countries. Cascade: 7 countries. Phys. Inac.: Physical Inactivity. | |

**Fig M.** Percentages of countries with significant sex differences in health pathways of HIV and AIDS, by region (significant when non-overlapping confidence intervals of estimates between females and males).

| Europe and Central Asia* | |
| --- | --- |
| 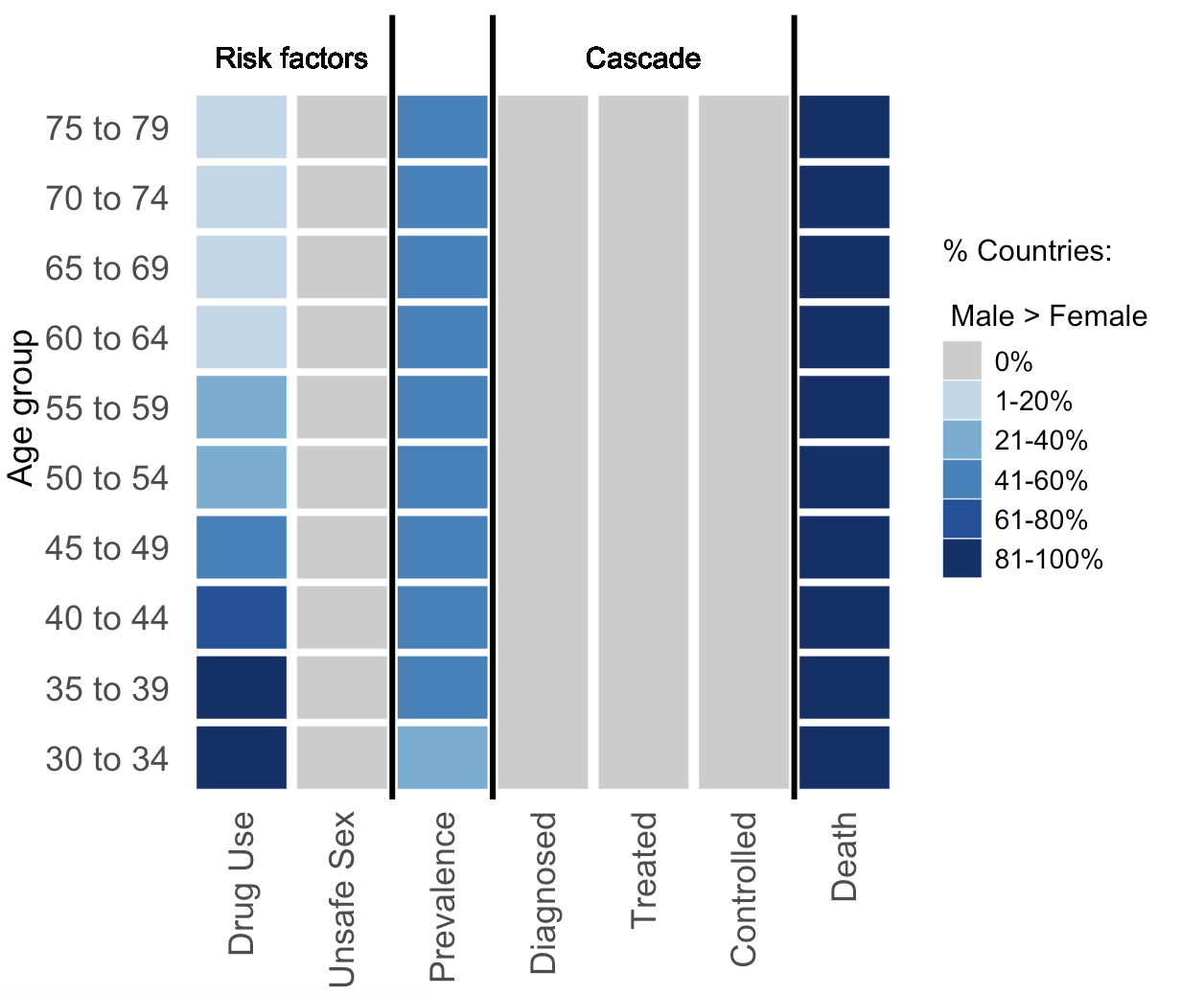 | 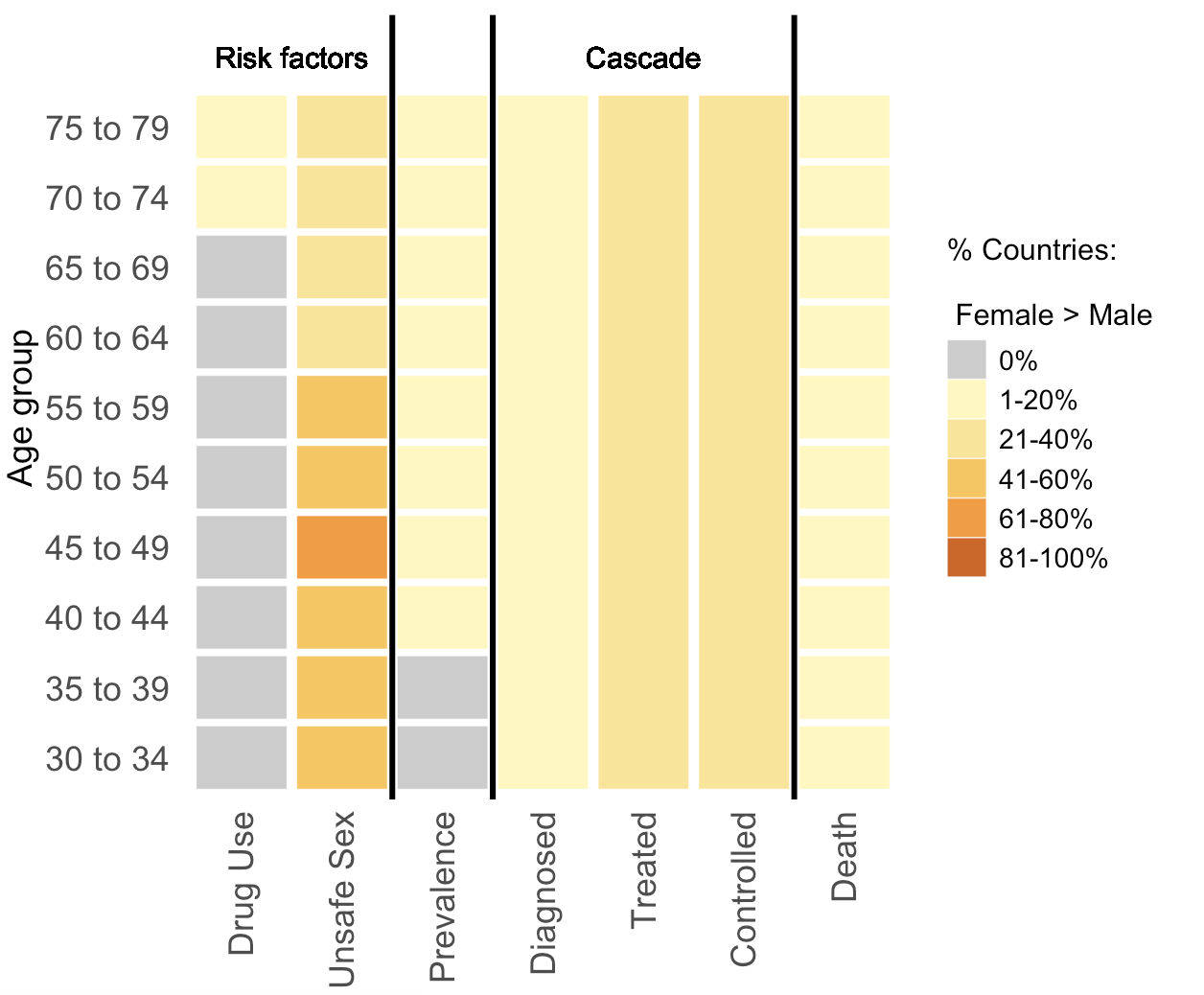 |
| * Risk factors, prevalence, death: 52 countries. Cascade: 20 countries. Phys. Inac.: Physical Inactivity. | |
| South Asia* | |
| 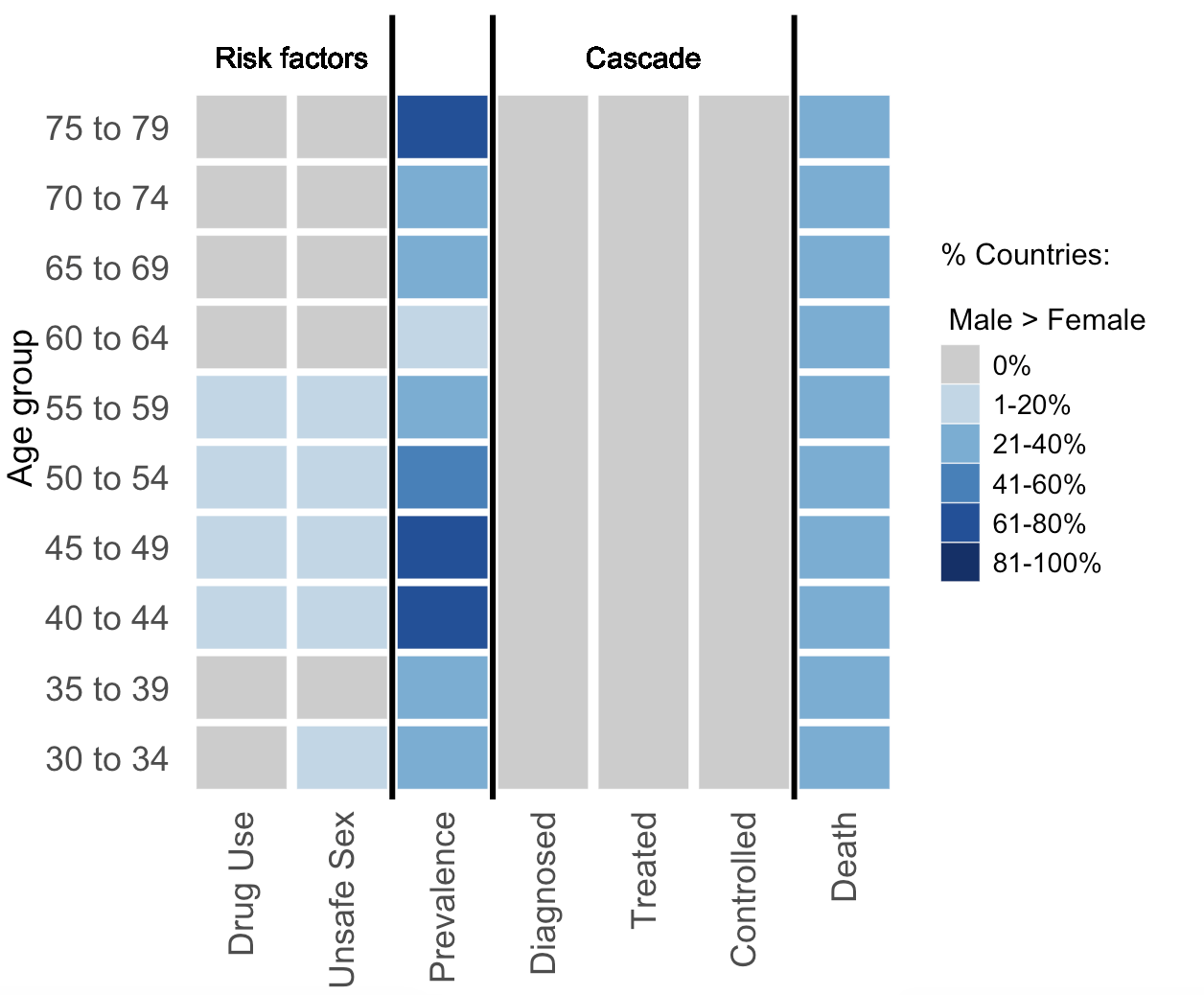 | 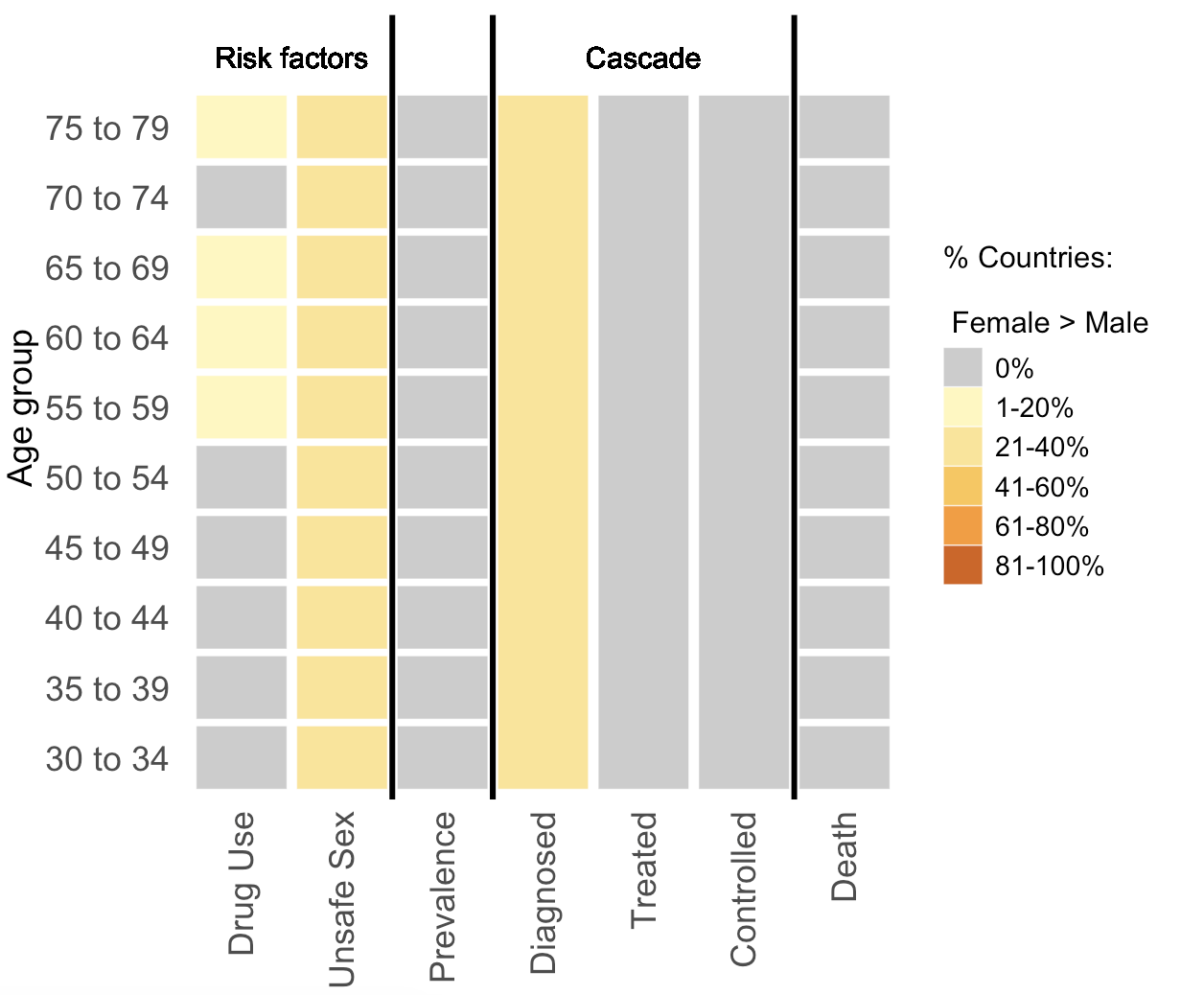 |
| * Risk factors, prevalence, death: 8 countries. Cascade: 3 countries. Phys. Inac.: Physical Inactivity. | |
| Sub-Saharan Africa* | |
| 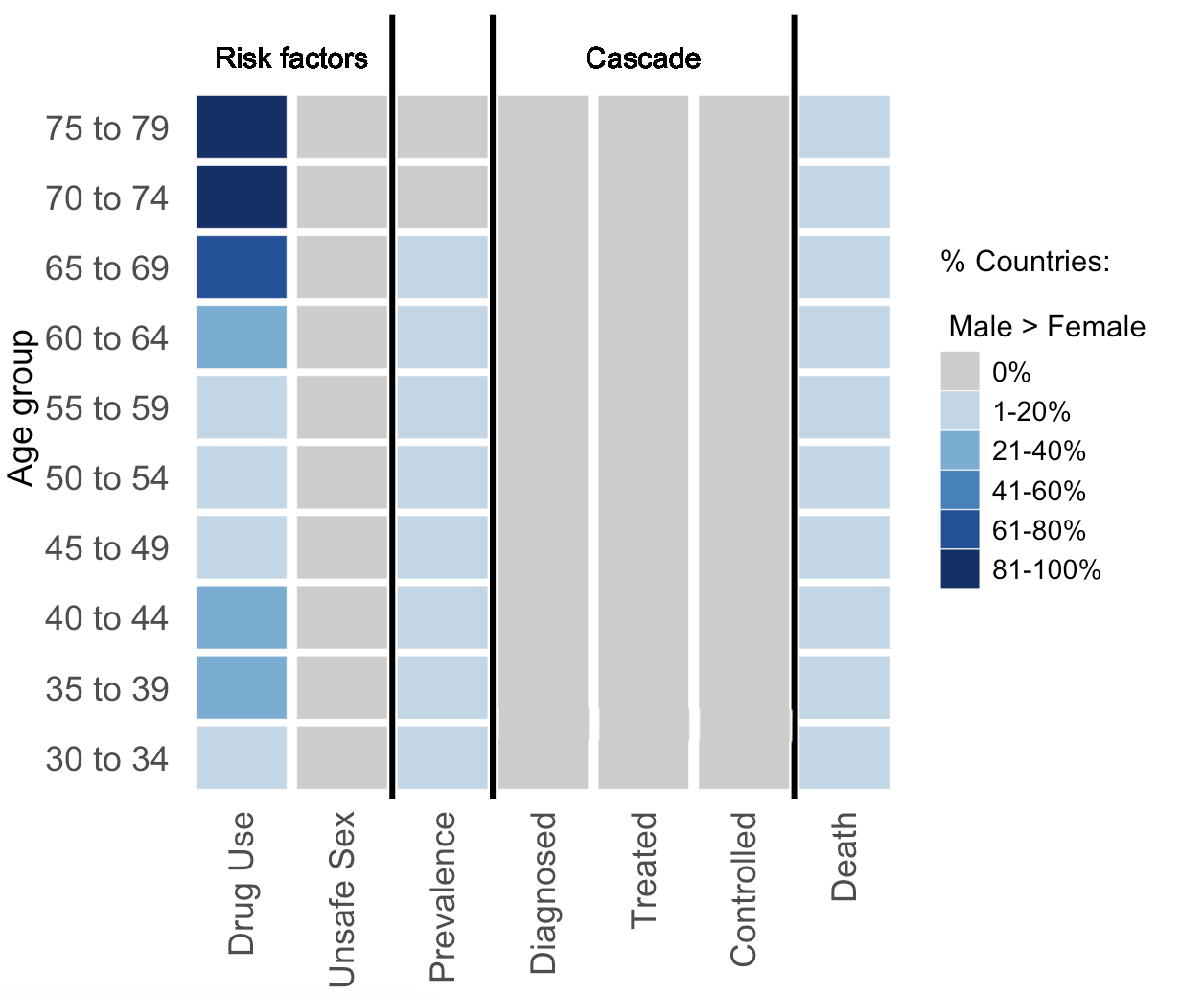 | 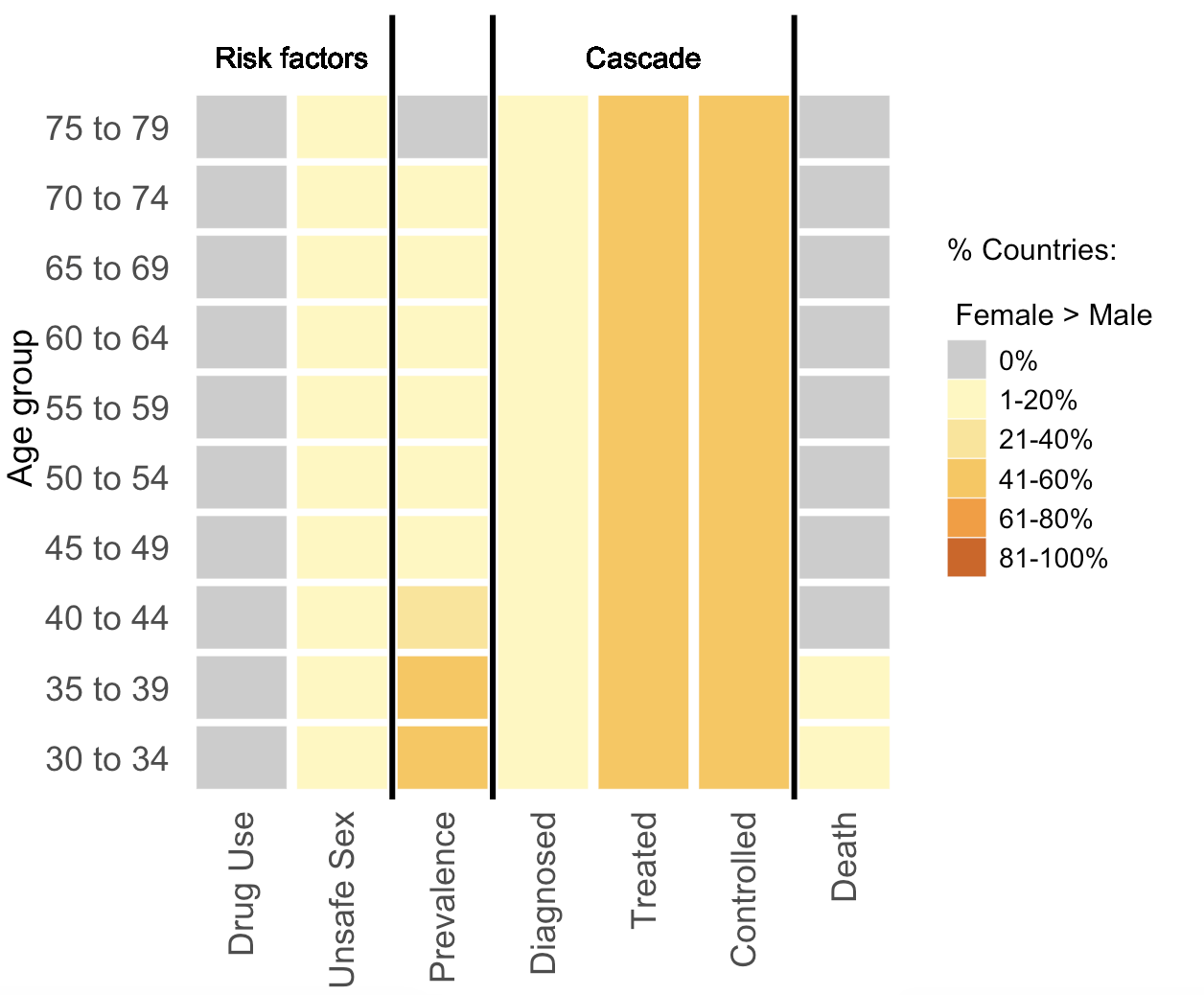 |
| * Risk factors, prevalence, death: 44 countries. Cascade: 21 countries. Phys. Inac.: Physical Inactivity. | |
| Middle East and North Africa* | |
| 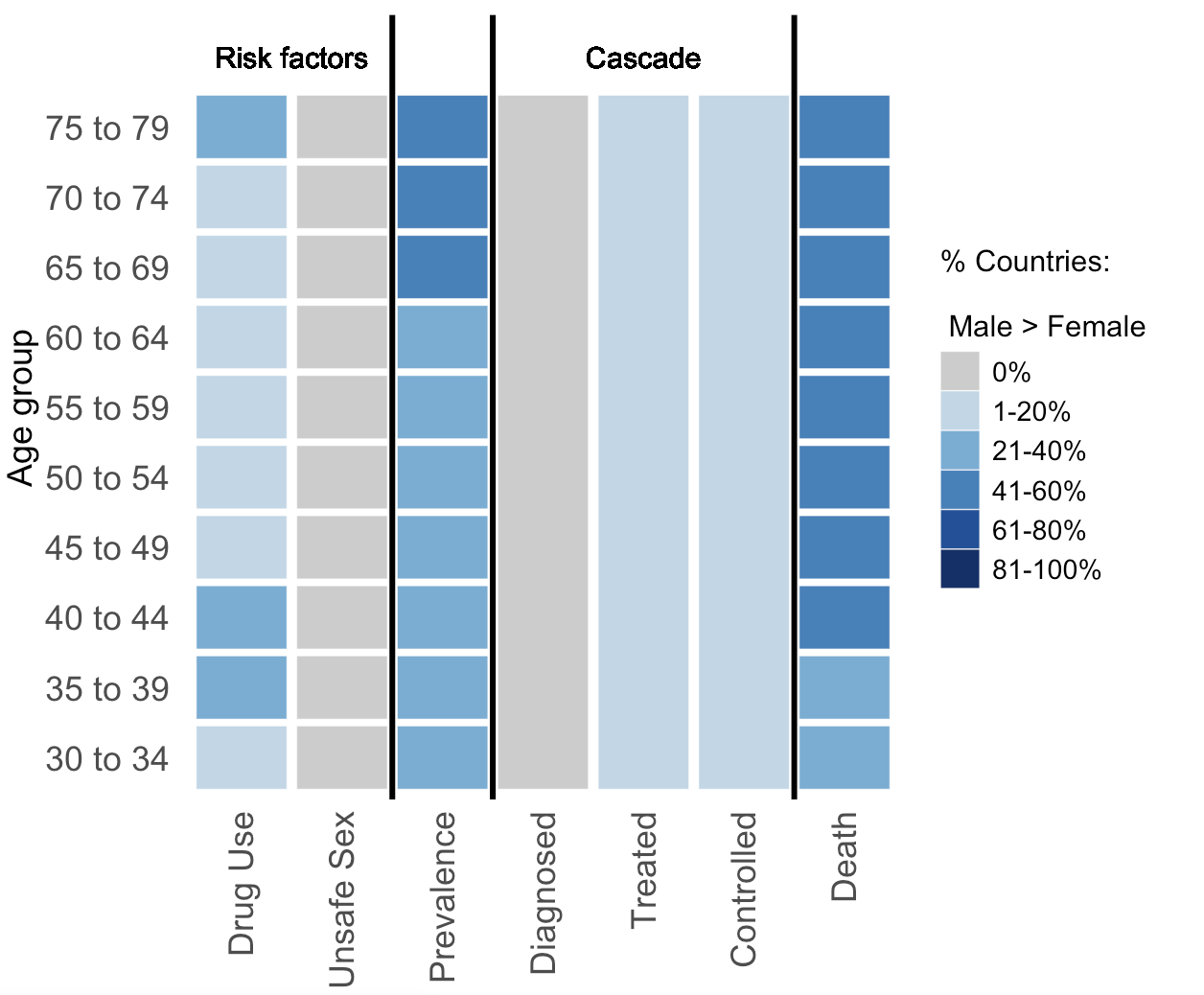 | 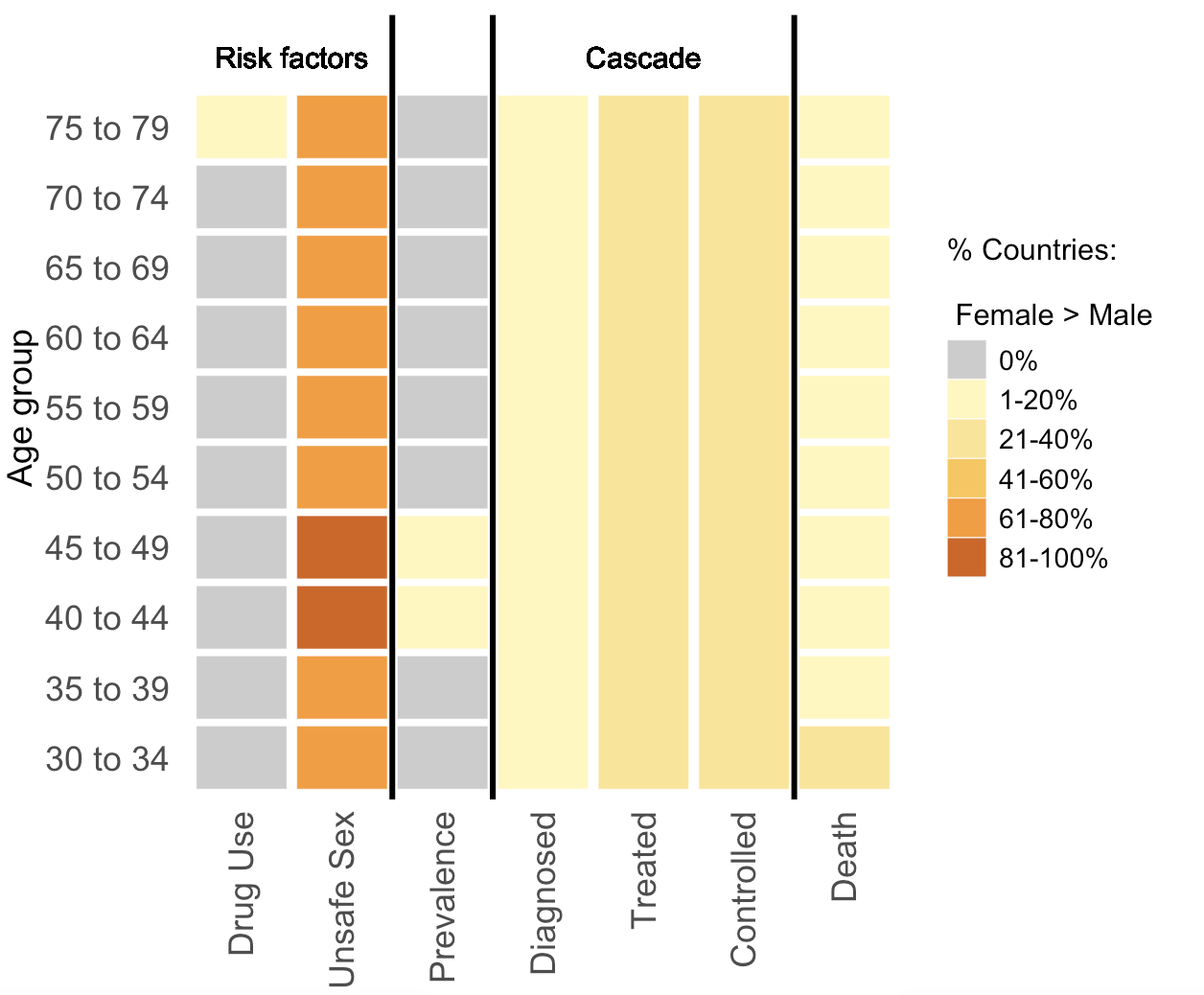 |
| * Risk factors, prevalence, death: 22 countries. Cascade: 8 countries. Phys. Inac.: Physical Inactivity. | |
| Latin America & the Caribbean* | |
| 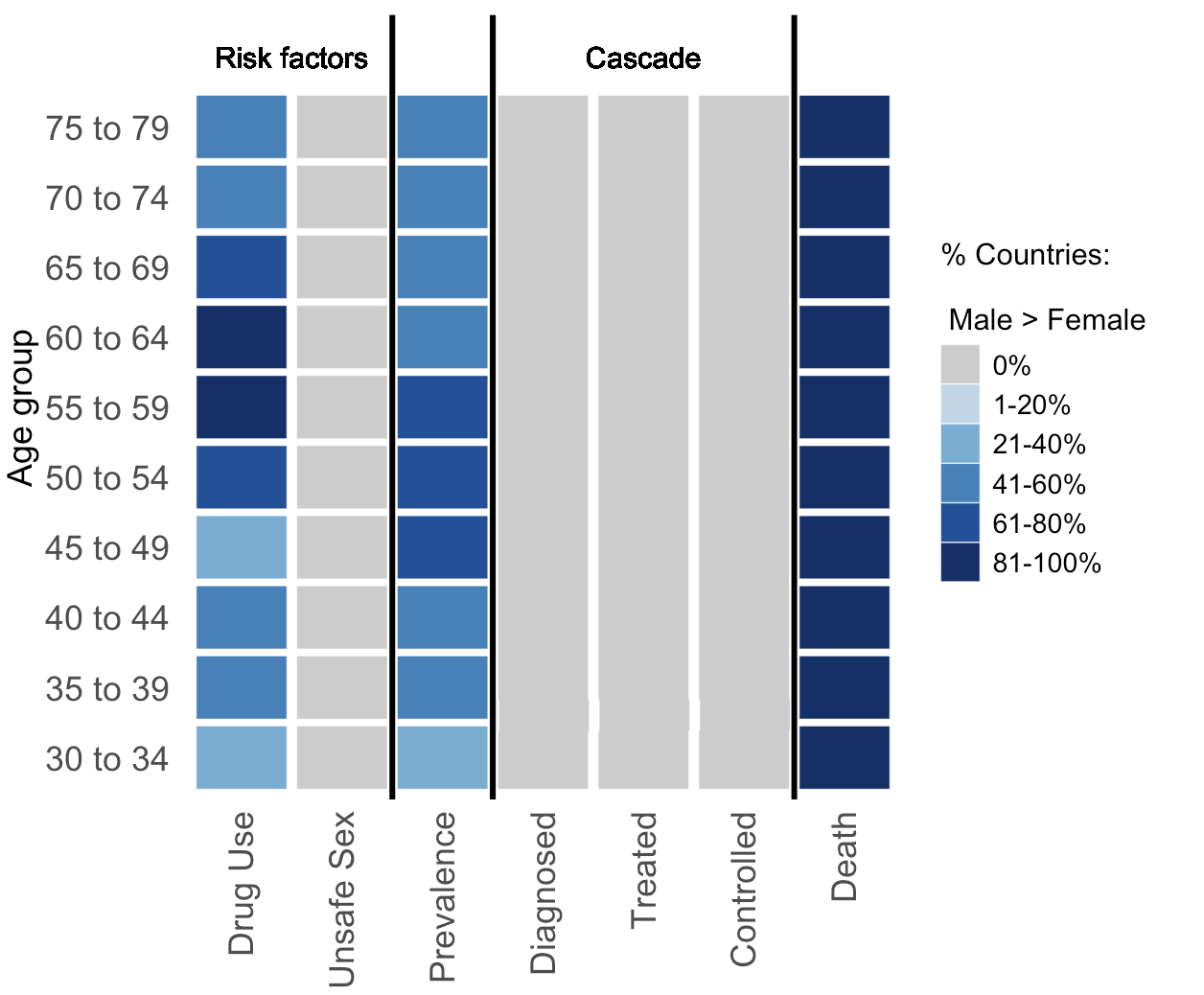 |  |
| * Risk factors, prevalence, death: 38 countries. Cascade: 16 countries. Phys. Inac.: Physical Inactivity. | |
| East Asia and Pacific* | |
|  |  |
| * Risk factors, prevalence, death: 34 countries. Cascade: 8 countries. Phys. Inac.: Physical Inactivity. | |
| North America* | |
|  |  |
| * Risk factors, prevalence, death: 3 countries. Cascade: No countries. Phys. Inac.: Physical Inactivity. | |
