# Supplementary material for: Sex-disaggregated data along the gendered health pathways: A review and analysis of global data on hypertension, diabetes, HIV, and AIDS
Source: PLoS Med. 2025 May 1;22(5):e1004592. doi: 10.1371/journal.pmed.1004592 (PMC12045488; doi:10.1371/journal.pmed.1004592)
Supplement: Attachment — Submitted filename: Plos review.docx [file pmed.1004592.s002.docx]

Thank you for this important analysis. I found the paper interesting, useful, and well done. Here are some points for consideration in both the framing and presentation of data that I think would be important to expand on in subsequent drafts.

1. For the other diseases for which global sex-disaggregated data were not available, was it that there were good global data, but it just wasn’t disaggregated or that theses diseases lacked globally comparable data in general? It would be useful to clarify this, since there are different recommendations depending on the available data.
2. It is difficult to understand the author’s statistical approach from the methods section alone, without reading the annexes. It would be useful to add some of the details from the annex into the last paragraph of the methods section. For example, detail your primary outcomes, “exposure”, and covariates, how many models you built, variable selection for final models etc.
3. I note that the authors have presented stratified results by world region and income level, which is useful. Additionally, I think it would be interesting to examine the effect of country income as an effect modifier between sex and health outcomes. From reviewing the tables, it seems that women are experiencing poorer outcomes in sub-Saharan Africa and maybe other LICs while the inverse is true for men. When the results are presented just as global counts of countries where men and women are experiencing poorer health we may be missing a key part of the story – that country income level really matters for sex differences.
4. I think a more nuanced discussion of how sample bias may be impacting these results in warranted. For example, sex-disaggregated data may be more available in high-income countries (which may also be the countries where men are experiencing poorer health). I think a discussion about how global data availability, country-income level, and health disparities by sex/gender are interacting and impacting these results is warranted.
