# Supplementary material for: Sex-disaggregated data along the gendered health pathways: A review and analysis of global data on hypertension, diabetes, HIV, and AIDS
Source: PLoS Med. 2025 May 1;22(5):e1004592. doi: 10.1371/journal.pmed.1004592 (PMC12045488; doi:10.1371/journal.pmed.1004592)
Supplement: Attachment — Submitted filename: Letter for reviewers .docx [file pmed.1004592.s003.docx]

**Response Letter to the Reviewers**

Comments from the academic editor:

The academic editor was supportive of the decision to issue a major revision outcome. However, he also had some comments. Two of his comments pertain to suggestions raised by some of the reviewers:

(a) the need for more detail on what models were done and

(b) the need for more detail on the sample selection issue. For (b), he noted that the different diseases use a different selection of countries, and this would likely have an impact on how findings could be compared across diseases, such as diabetes vs hypertension.

He also commented that, given that GBD 2021 was released some months ago now, and, additionally, NCD RiSC recently released their diabetes prevalence and treatment paper, the authors may wish to incorporate these new estimates into their study.

Finally, he added that the authors state their search of STEPS surveys since 2013 found n=39 countries – he wondered whether the authors could elaborate in their revised manuscript on why the search was restricted to 2013, and why it appears the search is some years out-of-date.

Dear Editor and Reviewers,

Thank you for the opportunity to revise and resubmit our manuscript ID PMEDICINE-D-24-03084R1 “We Need More Sex-Disaggregated Data: Unveiling the Data Gap and Showing the Benefit of Sex-Disaggregated Data Along the Health Pathways for Hypertension, Diabetes, HIV and AIDS”.

We thank the Editor and the Reviewers for their constructive feedback and suggestions, which have significantly improved the manuscript.

Below is a point-by-point response to the reviewer's comments. Authors’ comments are highlighted in blue and revisions in the manuscript are marked in red.

In response to the reviewers' concerns, we have revised the analysis to focus on 2019 HIV care cascade data rather than 2021, ensuring consistency across analyses and avoiding potential biases caused by COVID-19-related disruptions. This adjustment has been explained in the revised manuscript (see section on Scoping Review). Sex differences in the care cascade for HIV in 2019 are generally consistent with the sex differences in 2021, though in 2019 slightly more countries reported significant sex differences in the HIV cascade steps, especially controlled females (21 out 76 countries with available HIV care cascade data in 2019, 28%, compared to 7 out 73 countries with available HIV care cascade data in 2021, 10%).

To sharpen the focus and enhance clarity, we have also removed the regression analysis linking sex differences in care cascades to sex-specific socio-economic variables and risk factors. This decision ensures the manuscript emphasizes its key findings and primary contributions.

Comments from the reviewers:

**Reviewer #1:**

This manuscript fills a key meta-data gap at the global level. It was very informative to see data presented on risk factors, disease prevalence and mortality for a range of diseases across most countries in the world. The sex and age disaggregation of data provide richer context and nuance to the findings.

Having said that, sometimes sex and gender are used interchangeably in the paper, which is confusing. Some specific examples and recommendations:

1. The title reads "We need more sex-disaggregated data…" but the first sentence of the abstract reads "Health data disaggregated by sex or gender identity is vital…".

Based on the discussion section in the paper, the authors presumably want to say in the abstract that health data disaggregated by sex AND gender are vital. And in the title, I recommend that the authors decide if they want the take home message of this manuscript to be that we need more sex-disaggregated data or whether we need more sex AND gender-disaggregated data. It is entirely appropriate for the authors to decide one way or the other, since both sex and gender disaggregated data are vital to understanding the world' pressing health problems (and for designing appropriate solutions to those problems). But right now, sex is referred to in some places, gender is mentioned in other places, and it is confusing when the terms are used interchangeably.

Thank you for this insightful comment. We have removed from the abstract the phrase "or gender identity" and clarified the distinction between sex and gender throughout the manuscript. We have emphasized that the data analyzed in this study are reported by sex rather than gender identity, as the datasets we analyzed provided only a single identifier for sex or gender identity without clearly distinguishing between them. To the best of our knowledge, all global-level health data are reported by sex rather than gender. These revisions have been incorporated in the introduction (see page 3), to ensure consistent and precise terminology throughout.

Sex and gender significantly shape health outcomes including through their individual or combined influence on a range of factors, such as rates of exposure to risk factors and environments, individual bodily responses to risk exposure, and patterns of health service use (National Academies of Sciences, Engineering, and Medicine, 2022). For example, gender, as a social construction, influences how men and women have different exposure to health-harming products such as tobacco, alcohol, and poor nutrition. Sex plays an important role in health outcomes, including by determining physiological responses to gendered risk exposures (e.g. male and female bodies metabolise alcohol differently), while gender influences health care access and the quality of care received from health services (Mauvais-Jarvis et al., 2020; Naicker et al., 2014).

Understanding differences in risk exposure, health service use, and health outcomes by sex and gender identity (i.e. whether someone identifies as a woman, man or another gender identity) is vital to the identification of interventions which can effectively reduce health inequities, e.g., ensuring that interventions are tailored to the specific needs of women, men, and gender-diverse individuals. However, sex and gender identity are often confused and conflated in health surveys (National Academies of Sciences, Engineering, and Medicine, 2022). As a result, analysis of survey data is challenging - were researchers reporting on sex or gender identity? Does the original language of the survey have different terms to distinguish between sex and gender identity? Additionally, very few surveys outside of a few specific country contexts report on gender identity beyond a simple binary (woman/man) (Heidari et al., 2016). In this study we report on data from datasets and surveys as being sex-disaggregated, but interpret any observed differences through the lens of gender analysis - which enables identification of modifiable factors for sex- and gender-responsive interventions.

2. On page 11, there is a sentence that states "Despite these sex and gender differences, health policies often do not include sex- and gender-specific/responsive approaches." From the results, however, there are no gender differences presented because - as the authors point out - no adequate gender-disaggregated data were available in the data sources they examined. I recommend that the authors delete "gender" in the sentence. The authors have already explained in the discussion section that one key limitation of the data sources they examined was that they did not have adequate gender disaggregated data.

Thank you for this recommendation.In the introduction section, we have explained that this study reports on survey and health service data disaggregated by sex, interpreting observed differences through the lens of gender analysis to identify modifiable factors for sex- and gender-responsive interventions (see page 3, line 19). Furthermore, the sentence highlighted has been revised as follows (see page 12, last paragraph).

[...] In this study we report on data from datasets and surveys as being sex-disaggregated, but interpret any observed differences through the lens of gender analysis - which enables identification of modifiable factors for sex- and gender-responsive interventions.

[...] However, despite a strong evidence base confirming the importance of sex and gender as determinants of disease, health policies often do not include sex-specific responsive approaches.

**Reviewer #2:**

Thank you for this important analysis. I found the paper interesting, useful, and well done. Here are some points for consideration in both the framing and presentation of data that I think would be important to expand on in subsequent drafts.

1. For the other diseases for which global sex-disaggregated data were not available, was it that there were good global data, but it just wasn't disaggregated or that theses diseases lacked globally comparable data in general? It would be useful to clarify this, since there are different recommendations depending on the available data.

Thank you for raising this important point. The primary data gap pertains to the cascade steps (e.g., diagnosis, treatment, and control) rather than to metrics such as risk factor prevalence, disease prevalence, or mortality rates. Through our scoping review we identified three main issues: 1) a general lack of globally comparable cascade data, 2) among the available cascade data, a frequent absence of sex and/or age disaggregation; 3) even when cascade data are disaggregated by sex, studies often fail to address or discuss sex-related differences. To address this point, we have clarified the issue of data availability and its implications in the Scoping Review section of the revised manuscript (see page 4).

**Scoping review for the care cascade data and selection of diseases**

Recognising the scarcity of data on the care cascade compared to risk exposure and health outcomes, we searched for published papers using global datasets on the sex-disaggregated care cascade for eight major conditions and causes: hypertension, diabetes, HIV and AIDS, tuberculosis, dementia, depression, chronic obstructive pulmonary disease, and lung cancer. We looked for global-level datasets that report the cascade steps by sex for more than five countries. More specifically, we searched on MEDLINE (via PubMed) for articles published sex- or gender-disaggregated data on each step of the health pathway until March 26th, 2024. Our search criteria included specific terms: (“Care cascade” OR “cascade of care” OR ((diagnosis OR detection) AND (medication OR treatment) AND (control OR aware*))) AND ((sex-specific OR sex-disaggregated OR “by sex”) OR (gender-specific OR gender-disaggregated OR “by gender”)) AND (trend* OR global OR worldwide). We made no language restrictions. Through the scoping review we identified three key issues related to data gaps and availability: 1) a general lack of globally comparable cascade data, 2) among the available cascade data, a frequent absence of sex and/or age disaggregation; 3) even when cascade data are disaggregated by sex, studies often fail to address or discuss sex-related differences. The review identified global datasets meeting these criteria only for hypertension, diabetes, and HIV and AIDS. These three conditions were selected for further analysis, shaping the subsequent research methods.

2. It is difficult to understand the author's statistical approach from the methods section alone, without reading the annexes. It would be useful to add some of the details from the annex into the last paragraph of the methods section. For example, detail your primary outcomes, "exposure", and covariates, how many models you built, variable selection for final models etc.

Thank you for highlighting this point. To better clarify the key results and messages of this paper, we decided to remove the results of the regression analysis examining associations between sex differences in the cascade of care for hypertension, diabetes, and HIV/AIDS and sex-specific socio-economic variables and risk factors. This decision was made to maintain emphasis on the most relevant findings and insights presented in the manuscript (see section of methods, pages 5-7).

**Data sources**

**Risk Factors, Disease Prevalence, and Death Rates**

Data on risk factors, disease prevalence, and death rates were obtained from the Global Burden of Disease (GBD) dataset. Although the GBD database presents some limitations (GBD, 2021), it remains the most reliable resource for comprehensive global data on risk factors and disease prevalence. While in-country data are valuable, the GBD data are standardised - meaning it is possible to undertake comparisons across countries (Murray et al., 2020; Vos et al., 2020; Roth et al., 2018). To align with the care cascade data and ensure consistency in analyses, the study relied on 2019 data for risk factors, prevalence, and death rates from the GBD 2021 release. [...]

**Cascade of Care**

[...] To ensure consistency and avoid potential biases from COVID-19-related disruptions, the study focused on data from 2019 for hypertension and HIV and AIDS care cascades. Hypertension cascade data for 2019 were available for all included countries, while HIV and AIDS care cascade data were complete for 76 countries. For diabetes, given the limited availability of care cascade data across years and countries, the study pooled data from 2013-2020, yielding information for 39 countries.

**Statistical Analysis**

We examined the sex differences along the health pathway, including risk factor exposure, disease prevalence, stages of the care cascade, and death rates for each condition. We identified significant sex differences in health pathway steps by comparing the 95% confidence intervals between females and males, considering steps with non-overlapping intervals as statistically different. This method provides strong statistical power by adopting a stringent significance threshold (i.e., 0.05 × 0.05 = 0.0025, resulting in a significance level of 0.25%) (Nakagawa and Cuthill, 2007). As confidence intervals for diabetes care cascade were unavailable, we calculated exact 95% binomial confidence intervals. [...]

3. I note that the authors have presented stratified results by world region and income level, which is useful. Additionally, I think it would be interesting to examine the effect of country income as an effect modifier between sex and health outcomes. From reviewing the tables, it seems that women are experiencing poorer outcomes in sub-Saharan  Africa and maybe other LICs while the inverse is true for men. When the results are presented just as global counts of countries where men and women are experiencing poorer health we may be missing a key part of the story - that country income level really matters for sex differences.

The authors appreciate the reviewer’s observation. To address this, country-specific GDP per capita was included in the regression analysis to assess its influence on sex-specific pathway outcomes. Our findings show that GDP per capita is positively associated with both the treated step (step 2, conditional on being diagnosed) and the controlled step (step 3, conditional on being treated). These results align with your observation on the role of country income levels in shaping sex-specific health outcomes. However, to better clarify the key results and messages of this paper, we decided to remove the results of the regression analysis examining associations between sex differences in the cascade of care for hypertension, diabetes, and HIV/AIDS and sex-specific socio-economic variables and risk factors. However, to sharpen the focus and enhance clarity of the manuscript, we have removed the regression analysis linking sex differences in care cascades to sex-specific socio-economic variables and risk factors.

4. I think a more nuanced discussion of how sample bias may be impacting these results in warranted. For example, sex-disaggregated data may be more available in high-income countries (which may also be the countries where men are experiencing poorer health). I think a discussion about how global data availability, country-income level, and health disparities by sex/gender are interacting and impacting these results is warranted.

Thank you for this observation. We agree that a nuanced discussion of sample bias and its potential impact on the results is warranted. To address these issues, we have discussed how sample bias and the interaction between global data availability, income levels, and sex/gender disparities might influence the interpretation of these results (see page 13, line 11).

Secondly, the data available may be affected by sample bias including the underrepresentation of certain populations, and inconsistencies in data collection methods. Reliance on national surveys may result in the exclusion or under-representation of some populations such as those marginalised by sexual or gender identity, or people living in geographically remote or in challenging conditions such as conflict zones. Self-reported data, varying definitions, inconsistencies in measurement techniques and varying definitions of health conditions further complicate comparability.

**Reviewer #3:**

The manuscript presents a scoping review to identify gender and sex differences in the epidemiology (exposure, prevalence, care cascade and mortality) for several major health conditions. The authors identify several consistent sex-based differences for hypertension, diabetes and HIV/AIDS. The authors make a plea for wider availability of sex/gender- and age-disaggregated data. This is a very interesting manuscript with an important message. I think the manuscript can be improved by greater clarity on the data search and inclusion criteria and a clearer presentation with maybe a slightly deeper discussion of the results.

PURPOSE OF REVIEW

This is a statistical review and focuses foremost on statistical rigour of the study, though I do add some less statistical comments. I note that this is not a statistics-heavy paper, so my review is relatively light-touch and I really only have 2 purely statistical comments (comments O.3 and M.3 in the comments below).

OVERALL ASSESSMENT

This is a very interesting study and I broadly agree with the authors' plea for more sex-disaggregated data being made available. I have several overall comments, with the most pertinent comment probably being that I do not think it is very clear at present hwo datsets were identified and selected for inclusion in / exclusion from the study (several of my comments touch upon this).

O.1: While the authors state that they searched "for published papers using global datasets on the sex-disaggregated care cascade", yet only used data from the Global Burden of Disease (GBD) 2019. For the care cascade 3 sources were used (NCD-RisC, STEPS and UNAIDS). I have 2 questions regarding the use of GBP 2019:

1) As far as I know GBD 2021 data are available, why were these not used?

Thank you for bringing this up. The analysis shown in the present study used risk factors, disease prevalence, and death rates data from the 2021 release of GBD (GBD 2021). Although the GBD 2021 data include information on risk factors, disease prevalence, and mortality rates up to 2021, the hypertension cascade of care data were only available up to 2019. For diabetes, care cascade data were available over the period 2013-2020, with availability varying by country. For HIV and AIDS, care cascade data were available over the period 2015-2021 but we selected data in 2019 to keep consistency across conditions. Consequently, to maintain consistency also across the analyses, the study used the 2019 data on risk factors, prevalence, and death rates, also for diabetes and HIV and AIDS. This approach also minimizes potential biases introduced by COVID-19-related disruptions, which could disproportionately affect later data.

While GBD has limitations, it remains the most comprehensive global resource for risk factors and disease prevalence data. Unlike other databases, such as the WHO’s Global Health Estimates, which focus on cause-of-death statistics but lack disease and risk factor prevalence, GBD provides a combination of detailed and globally extensive data. We have explained this point in the section data sources of the revised manuscript (see page 5, section of risk factors, disease prevalence, and death rates).

**Risk Factors, Disease Prevalence, and Death Rates**

Data on risk factors, disease prevalence, and death rates were obtained from the Global Burden of Disease (GBD) dataset. Although the GBD database presents some limitations (GBD, 2021), it remains the most reliable resource for comprehensive global data on risk factors and disease prevalence. While in-country data are valuable, the GBD data are standardised - meaning it is possible to undertake comparisons across countries (Murray et al., 2020; Vos et al., 2020; Roth et al., 2018). To align with the care cascade data and ensure consistency in analyses, the study relied on 2019 data for risk factors, prevalence, and death rates from the GBD 2021 release.

2) While GBD data are fairly comprehensive and definitely global, there are datsets outside of GBD. I would expect that some of the data that the authors highlight as not being available, are probably available on a country-by-country basis, with varying data access requirements and varying data quality (e.g. from routinely collected data, from surveys, ...). I do realise that it is highly impractical to navigate all of these issues for the kind of study presented here and the authors are transparent that they only considered "global" datasets, but I do not think the paper currently is very clear how datsets were searched for and what the exact criteria were for inclusion in the study. Can the authors add details of other datasets that were considered but ultimately decided not to be used (and for what reason)? This would also be useful to understand the dataset identification and selection for the care-cascade analysis. If it was an active decision to only use GBD data, then I think this should be very clearly stated and acknowledged as a limitation.

We agree with the reviewer that we should state our search criteria clearer. Following the suggestion we have extensively revised the section of scoping review. The section includes a detailed explanation of the inclusion criteria of the study (see page 4).

**Scoping review for the care cascade data and selection of diseases**

Recognising the scarcity of data on the care cascade compared to risk exposure and health outcomes, we searched for published papers using global datasets on the sex-disaggregated care cascade for eight major conditions and causes: hypertension, diabetes, HIV and AIDS, tuberculosis, dementia, depression, chronic obstructive pulmonary disease, and lung cancer. We looked for global-level datasets that report the cascade steps by sex for more than five countries. More specifically, we searched on MEDLINE (via PubMed) for articles published sex- or gender-disaggregated data on each step of the health pathway until March 26th, 2024. Our search criteria included specific terms: (“Care cascade” OR “cascade of care” OR ((diagnosis OR detection) AND (medication OR treatment) AND (control OR aware*))) AND ((sex-specific OR sex-disaggregated OR “by sex”) OR (gender-specific OR gender-disaggregated OR “by gender”)) AND (trend* OR global OR worldwide). We made no language restrictions. Through the scoping review we identified three key issues related to data gaps and availability: 1) a general lack of globally comparable cascade data, 2) among the available cascade data, a frequent absence of sex and/or age disaggregation; 3) even when cascade data are disaggregated by sex, studies often fail to address or discuss sex-related differences. The review identified global datasets meeting these criteria only for hypertension, diabetes, and HIV and AIDS. These three conditions were selected for further analysis, shaping the subsequent research methods.

Moreover, the authors agree with the reviewer that additional data may be available from in-country sources. However, the focus of this study was on global comparisons, which require standardized data to ensure fair and consistent comparisons among countries. In-country data, while valuable, can vary in quality, methodologies, and definitions, making it challenging to harmonize them for global analysis. We have clarified this point in the section Risk Factors, Disease Prevalence, and Death Rates of the revised manuscript (see page 5).

**Risk Factors, Disease Prevalence, and Death Rates**

Data on risk factors, disease prevalence, and death rates were obtained from the Global Burden of Disease (GBD) dataset. Although the GBD database presents some limitations (GBD, 2021), it remains the most reliable resource for comprehensive global data on risk factors and disease prevalence. While in-country data are valuable, the GBD data are standardised - meaning it is possible to undertake comparisons across countries (Murray et al., 2020; Vos et al., 2020; Roth et al., 2018). To align with the care cascade data and ensure consistency in analyses, the study relied on 2019 data for risk factors, prevalence, and death rates from the GBD 2021 release.

O.2: I am not sure the authors go far enough with their plea. We do not just need more data, but also standardised data collection tools, analysis frameworks and reporting guidelines to make it easier to conduct secondary, cross-country analyses that can drill down into sub-populations etc. This would have to be lead by large international bodies or consortia (e.g. WHO, GBD, ...).

Thank you for this important point. In addition to more data, there is indeed a pressing need for standardized data collection tools, consistent analysis frameworks, and harmonized reporting guidelines. We have extended the discussion on this point in the revised manuscript (see page 10 and page 14).

[...] While some studies have analysed trends in risk factors, prevalence and treatment for the three conditions we have reviewed, these studies are generally limited by absence of sex disaggregation (e.g., Rahim et al., 2023; Ochmann et al., 2023; Manne-Goehler et al., 2019) or limited geographical scope. For example, a recent analysis of global trends in hypertension prevalence and treatment progress from 1990 to 2019 using data from the NCD Risk Factor Collaboration did not include assessment of sex differences (Zhou et al., 2024; Zhou et al., 2021). Other studies have been limited by their geographical coverage (Zhang et al., 2023). Three papers reported on diabetes risk and preventive measures across low- and middle-income countries and on diagnostic testing for hypertension, diabetes, and hypercholesterolemia within similar economic groups (Rahim et al., 2023; Ochmann et al., 2023; Manne-Goehler et al., 2019), while another study explored the long-term and recent trends in hypertension awareness, treatment, and control across 12 high-income countries (Zhou et al., 2019). These studies provide valuable insights, but are limited to regional, national, or income group levels (Euvrard et al., 2024; Lee et al., 2022; Prenissl et al., 2019), and only a few include sex-disaggregated data, especially regarding the care cascade for specific diseases (Walli-Attaei et al., 2020).

[...] In addition to more disaggregated data, there is a need for inclusive data collection tools and harmonized reporting guidelines. This would enable more robust secondary analyses, including cross-country comparisons and in-depth exploration of subpopulations. Achieving this requires leadership from large international organizations or consortia, such as the WHO or GBD, to ensure global alignment and coordination. To assemble and publish standardised datasets for additional health pathways, several options present themselves. As directing and coordinating authority for international cooperation on health, WHO can standardize data across countries, but it faces resource limitations and many competing priorities. Entities such as Global Health 50/50, Data 2x or Equal Measures (https://equalmeasures2030.org/members/data2x/) can seek private and other funding and operate independently, but they lack the convening power and generally must rely on publicly available data. Condition-specific epistemic communities offer deep expertise in their areas, yet they focus on one condition at a time, making comparisons challenging. Therefore, a collaborative approach might be the most effective solution to more joined up data coalition and analysis along condition-specific pathways. Finally, future iterations of country pathways should incorporate more upstream legal and policy environments, as these significantly influence risk exposures.

O.3: The results as presented in this manuscript rely on the definition of statistical significance used by the authors. I do not think this definition is optimal. On p.6 at the top, the authors state "We determined sex differences by comparing the confidence intervals between females and males, and considered steps with non-overlapping confidence intervals as statistically significant." While technically this statement cannot be ruled to be incorrect (since it simply states a definition), what this statement implicitly suggests is this: if the 95% CIs for the estimates of the same parameter in 2 different groups do not overlap, then that difference in parameter estimates is statistically significant at the 5% significance level. That statement is not fully correct: the difference will certainly be significant at the 5% level, but it will also be significant at a much lower level. In fact, a parameter, estimated in 2 groups, can have a statistically significant (at the 5% level) difference between these groups yet the 95% confidence intervals for the 2 groups overlap. Generally a more valid rule would be to consider statistical significance if the point estimate of one group is not contained within the 95% CI of the other group. The current definition of statistical significance would be considerably lower than the commonly used 5% level (and while that 5% is arbitrary, it is commonly used and that is what most readers of the manuscript would take that your statistical significance implies).

We thank the reviewer for highlighting this relevant point. The reviewer’s rule of determining statistical significance based on whether the point estimate of one group falls outside the 95% confidence interval (CI) of the other group involves comparing both the point estimate of males against the 95% CI of females and vice versa. We noticed that this approach can yield asymmetric conclusions. For example, it could lead to findings such as:

- Females have a significantly larger proportion than males (e.g., the point estimate for females is outside the 95% CI for males), but
- Males do not have a significantly different proportion compared to females (e.g., the point estimate for males is within the 95% CI for females).

This asymmetry might seem counterintuitive and controversial. A common expectation is that if one group (e.g., females) is significantly different from another group (e.g., males), then the reverse should also hold; if females differ from males, males should also differ from females. However, this expectation does not always align if the point estimate of one group is compared with the confidence interval of the other groups and vice versa.

For example, hypertension diagnosis proportions for individuals aged 35–39 in France:

- Males: 0.32 (95% CI: 0.19–0.47)
- Females: 0.48 (95% CI: 0.31–0.66)

Here, the point estimate for females (0.48) is outside the 95% CI for males, indicating a statistically significant difference. However, the point estimate for males (0.32) falls within the 95% CI for females, suggesting no significant difference.

This asymmetry arises from the difference in the relative uncertainty of the estimates for each group. Notably, such asymmetry may complicate interpretation. Given the aim of the study to identify significant differences in health pathways, we argue that comparing the 95% confidence intervals between females and males is the most suitable approach. More specifically, this method provides stronger statistical power by adopting a more stringent significance threshold (i.e., 0.05 × 0.05 = 0.0025, resulting in a significance level of 0.25%). This stricter threshold reduces the likelihood of false positives, ensuring that observed differences are more robust.

The approach has been specified in the section of statistical analysis of the revised manuscript (see page 7).

We examined the sex differences along the health pathway, including risk factor exposure, disease prevalence, stages of the care cascade, and death rates for each condition. We identified significant sex differences in health pathway steps by comparing the 95% confidence intervals between females and males, considering steps with non-overlapping intervals as statistically different. This method provides strong statistical power by adopting a stringent significance threshold (i.e., 0.05 × 0.05 = 0.0025, resulting in a significance level of 0.25%) (Nakagawa and Cuthill, 2007). As confidence intervals for diabetes care cascade were unavailable, we calculated exact 95% binomial confidence intervals.

INTRODUCTION

(No comments from me on this section.)

METHODS

M.1: Unclear how the search was conducted before results were filtered down to those conditions for which disaggregated datasets for at least 5 countries were available. Also what was the range of publication dates for inclusion in this review? Would be good to have a supplement giving the exact search criteria so that the search could be reproduced.

Thank you for raising this important point. We have clarified how the search was conducted in the section of scoping review in the revised manuscript (see page 4).

M.2: Was the search for data limited to Global Burden of Disease? For most countries, there would be more data available from in-country sources. Did the authors try to access such data (I admit this would probably be a fairly mammoth task)?

The initial data search focused on identifying reliable, globally available, sex-disaggregated cascade of care data for common chronic conditions. We identified data for only three conditions (hypertension, diabetes, and HIV/AIDS) from different sources. Subsequently, we aimed to link the cascade of care data to information on risk factors, disease prevalence, and death rates. For this, we primarily relied on the Global Burden of Disease (GBD) dataset for two main reasons:

1. The GBD dataset is one of the most reliable and comprehensive global repositories, covering risk factors, diseases, and causes of death.
2. It already contained all the necessary information to be linked with the cascade of care data we identified.

This point has been clarified in the section data sources of revised manuscript (see page 5, section of risk factors, disease prevalence, and death rates).

Regarding the suggestion to access in-country data, we agree that such data could provide richer insights and improve the granularity of analyses. However, as you correctly noted, this is a significant challenge given the complexities of identifying, accessing, and harmonizing data from multiple countries with varying quality, availability and languages. This is indeed part of our longer-term plan to enhance the comprehensiveness of our study. For the current work, our focus has been on building a framework using globally accessible datasets, which lays the foundation for future integration of in-country data.

M.3: p.6 at the top: The authors state that they calculated "asymptotic 95% binomial confidence intervals". In a frequentist analysis, all CIs are of course asymptotic. However, for a binomial CIs, these are more usually referred to as 'exact' CIs to contrast this with normal approximation based CIs for proportions (which in addition to the usual frequentist asymptotic interpretation of CIs, is further making use of the asymptotic distribution convergence to a normal distribution as dicdated by the Central Limit Theorem).

We agree with the reviewer. We have replaced asymptotic with exact in the manuscript (see page 7, section of statistical analysis, line 7).

“[…] exact 95% binomial confidence intervals.”

M.4: The methods section mentions multivariable regression models being used in hypertension and HIV to "identify socio-economic differences at country level and risk factors influencing sex differences". However the results section does not seem to report results from these analyses and also the discussion does not touch upon this. Please either remove this bit from methods if those regression analysis results will not be included or include and discuss those results.

Thank you for this recommendation. To better clarify the key results and messages of this paper, we decided to remove the results of the regression analysis examining associations between sex differences in the cascade of care for hypertension, diabetes, and HIV/AIDS and sex-specific socio-economic variables and risk factors.

RESULTS

R.1: Figure 2: Several comments for this figure: 1) clearer if this was labelled with panel labels (e.g. A, B, C, D, E, F), 2) colour scales are difficult to read - could you use palettes with higher contrast?, 3) for diabetes, I think the cascade can be shown on the same graphs -- yes the age groups are not the same but the boundaries are shared (i.e. for the cascade you can simply merge the cells for each set of 3 finer-scale age groups into a single cell.

Following the suggestion of the reviewer, figure 2 has been revised to include panel labels and colour palettes with higher contrast. In addition, the health pathways for one condition are shown on the same graph. Differences in the number of countries included in each step are specified in the note, and differences in age groups are reflected by aggregated cells across age groups (Panel C–Panel F) (see page 7).

Notice that for hypertension, risk factors, prevalence, cascade, and death rates refer to the same age groups (5-years age groups) and the same number of countries (200). For diabetes and HIV and AIDS, risk factors, prevalence, and death rates refer to 200 countries and 5-years age, whereas care cascade data were available for fewer countries and more aggregated age groups. Differences in the number of countries included in each step are specified in the footnotes, and differences in age groups are reflected by aggregated cells across age groups (Panel C–Panel F).

**Figure 2.** Sex differences (significant when non-overlapping confidence intervals of estimates between females and males) in global health pathways of hypertension, diabetes, and HIV and AIDS.

**Hypertension Health Pathways (200 countries)**

| **Panel A** | **Panel B** |
| --- | --- |
|  |  |

**Diabetes Health Pathways (200 countries)**

| **Panel C** | **Panel D** |
| --- | --- |
|  |  |

* Diabetes care cascade data are provided only for 39 countries and age groups 30-44, 45-59, and 60-79.

**HIV and AIDS Health Pathways (200 countries)**

| **Panel E** | **Panel F** |
| --- | --- |
|  |  |

* HIV and AIDS care cascade data are provided only for 76 countries and one age group 15+.

R.2: My main comment on the result shown here is that it seems to be mostly an illustrative analysis of what widely-available sex/gender- and age-disaggregated data would allow to do. The authors mainly present results but do not dig much deeper in any of the things they identify. E.g. the authors state "Moreover, by comparing countries along the health pathways, our approach allowed us to identify countries that do not follow the general trends in sex differences, allowing us to probe further into the reasons behind the discrepancies." but then do not do this additional probing in the present manuscript. Likewise, while the authors discuss potential hypotheses that could explain the observed sex/gender differences, they do not pursue any of these further. I think there could be scope here for going a bit further with what the analysis has found. This could be where one would need to go from global datasets to more country-specific datasets.

We thank the reviewer for the important insights and acknowledge the reviewer's suggestion to delve deeper into the observed discrepancies. To address this, we expanded the discussion section to include a detailed examination of specific cases where countries deviate from general trends in sex differences (see page 10).

R.3: This is not a very useful comment for the authors as I cannot think of a good suggestion for improving the presentation of results, but I still just want to state that since the authors present data on 3 conditions, each with multiple risk factors, and their care cascades, I find the results a bit difficult to navigate and getting a good sense of. Figure 2 and Tables 1-3 go a long way, but overall I am not sure I really 'got' the results.

Thank you for highlighting this point. We appreciate the reviewer’s acknowledgment of the value of Figure 2 and Tables 1–3 in conveying the data. To address your concerns, we have reorganized the section of results in the revised manuscript to improve clarity and flow. We aimed to make the findings more accessible and ensure the key takeaways are easy to grasp. We hope the revised section of results provides a clearer structure and enhances understanding the results (see page 7).

DISCUSSION
(No comments from me on this section.)

REPORTING

RP.1: The authors identify the study as a scoping review, but did not indicate that they followed scoping review reporting guidelines. The quality and usefulness of the manuscript can be increased by following PRISMA scoping review extension guidelines (<https://www.equator-network.org/reporting-guidelines/prisma-scr/>).

Thank you for the recommendation. We have extensively revised the section of scoping review for the care cascade data and selection of conditions, following the PRISMA scoping review extension guidelines (see page 4).

OTHER COMMENTS

OC.1: The last word ("age group") from the 1st paragraph in the results section this should be in the plural, i.e. "age groups".

Thank you for pointing out this. We have reviewed the paragraph and used the plural for age groups (see page 7).

OC.2: p.9 "this data" -- more common to consider 'data' to be a plural and hence it should be "these data". Please also check the rest of the manuscript for consistent plural use of 'data'.

Thank you for pointing out this. We have reviewed the manuscript and consistently used the plural for 'data'.

OC.3: p.9 "one-time period" should be "one time period".

Thank you for pointing out this. We have reviewed the manuscript and replaced one-time period with one time period (see page 11).

one time period

Additional references:

Colineaux, H., Soulier, A., Lepage, B., & Kelly-Irving, M. (2022). Considering sex and gender in Epidemiology: a challenge beyond terminology. From conceptual analysis to methodological strategies. Biology of sex Differences, 13(1), 23.

Euvrard, J., Timmerman, V., Keene, C. M., Phelanyane, F., Heekes, A., Rice, B. D., ... & Boulle, A. (2024). The cyclical cascade of HIV care: Temporal care engagement trends within a population-wide cohort. Plos Medicine, 21(5), e1004407.

Hawkes, S., & Buse, K. (2020). The politics of gender and global health. In C. McInnes, K. Lee, J. Youde (Eds.), The Oxford Handbook of Global Health Politics. Oxford University Press, USA. doi:10.1093/oxfordhb/9780190456818.001.000

Heidari, S., Babor, T. F., De Castro, P., Tort, S., & Curno, M. (2016). Sex and gender equity in research: rationale for the SAGER guidelines and recommended use. Research integrity and peer review, 1, 1-9.

Lee, J., Wilkens, J., Meijer, E., Sekher, T. V., Bloom, D. E., & Hu, P. (2022). Hypertension awareness, treatment, and control and their association with healthcare access in the middle-aged and older Indian population: A nationwide cohort study. PLoS Medicine, 19(1), e1003855.

Mauvais-Jarvis, F., Merz, N. B., Barnes, P. J., Brinton, R. D., Carrero, J. J., DeMeo, D. L., ... & Suzuki, A. (2020). Sex and gender: modifiers of health, disease, and medicine. The Lancet, 396(10250), 565-582.

Murray, C. J., Aravkin, A. Y., Zheng, P., Abbafati, C., Abbas, K. M., Abbasi-Kangevari, M., ... & Borzouei, S. (2020). Global burden of 87 risk factors in 204 countries and territories, 1990–2019: a systematic analysis for the Global Burden of Disease Study 2019. The Lancet, 396(10258), 1223-1249.

Naicker, K., Liddy, C., Singh, J., Taljaard, M., & Hogg, W. (2014). Quality of cardiovascular disease care in Ontario’s primary care practices: a cross sectional study examining differences in guideline adherence by patient sex. BMC Family Practice, 15, 1-8.

Nakagawa, S., & Cuthill, I. C. (2007). Effect size, confidence interval and statistical significance: a practical guide for biologists. Biological reviews, 82(4), 591-605.

National Institutes of Health. (2021). To end HIV epidemic, we must address health disparities

Ochmann, S., von Polenz, I., Marcus, M. E., Theilmann, M., Flood, D., Agoudavi, K., ... & Vollmer, S. (2023). Diagnostic testing for hypertension, diabetes, and hypercholesterolaemia in low-income and middle-income countries: a cross-sectional study of data for 994 185 individuals from 57 nationally representative surveys. The Lancet Global Health, 11(9), e1363-e1371.

Prenissl, J., Manne-Goehler, J., Jaacks, L. M., Prabhakaran, D., Awasthi, A., Bischops, A. C., ... & Geldsetzer, P. (2019). Hypertension screening, awareness, treatment, and control in India: a nationally representative cross-sectional study among individuals aged 15 to 49 years. PLoS medicine, 16(5), e1002801.

Rahim, N. E., Flood, D., Marcus, M. E., Theilmann, M., Aung, T. N., Agoudavi, K., ... & Manne-Goehler, J. (2023). Diabetes risk and provision of diabetes prevention activities in 44 low-income and middle-income countries: a cross-sectional analysis of nationally representative, individual-level survey data. The Lancet Global Health, 11(10), e1576-e1586.

Roth, G. A., Abate, D., Abate, K. H., Abay, S. M., Abbafati, C., Abbasi, N., ... & Borschmann, R. (2018). Global, regional, and national age-sex-specific mortality for 282 causes of death in 195 countries and territories, 1980–2017: a systematic analysis for the Global Burden of Disease Study 2017. The Lancet, 392(10159), 1736-1788.

Schiebinger, L., Leopold, S. S., & Miller, V. M. (2016). Editorial policies for sex and gender analysis. The Lancet, 388(10062), 2841-2842.

Tolland, L., & Evans, J. (2019). What is the difference between sex and gender? Office for National Statistics.

Vos, T., Lim, S. S., Abbafati, C., Abbas, K. M., Abbasi, M., Abbasifard, M., ... & Bhutta, Z. A. (2020). Global burden of 369 diseases and injuries in 204 countries and territories, 1990–2019: a systematic analysis for the Global Burden of Disease Study 2019. The Lancet, 396(10258), 1204-1222.

World Health Organization (WHO 2014). Gender, climate change and health.

Zhou, B., Danaei, G., Stevens, G. A., Bixby, H., Taddei, C., Carrillo-Larco, R. M., ... & Ezzati, M. (2019). Long-term and recent trends in hypertension awareness, treatment, and control in 12 high-income countries: an analysis of 123 nationally representative surveys. The Lancet, 394(10199), 639-651.

Zhou, B., Rayner, A. W., Gregg, E. W., Sheffer, K. E., Carrillo-Larco, R. M., Bennett, J. E., ... & Can, G. (2024). Worldwide trends in diabetes prevalence and treatment from 1990 to 2022: a pooled analysis of 1108 population-representative studies with 141 million participants. The Lancet, 404(10467), 2077-2093.
