# Supplementary material for: Sex-disaggregated data along the gendered health pathways: A review and analysis of global data on hypertension, diabetes, HIV, and AIDS
Source: PLoS Med. 2025 May 1;22(5):e1004592. doi: 10.1371/journal.pmed.1004592 (PMC12045488; doi:10.1371/journal.pmed.1004592)
Supplement: Attachment — Submitted filename: Response to Editor_second rev.docx [file pmed.1004592.s004.docx]

Comments from the academic editor:

To me, this paper reads as though the authors had an a priori assumption that there is a paucity of sex-disaggregation, and this is the conclusion they were striving for. I think this is bias reflected not only in the title ("We Need More Sex-Disaggregated Data" -- not very subtle!) but also a lot in the discussion and what this study adds. For example, the authors strenuously and repeatedly argue that other global data groups such as NCD RisC and HPACC (disclaimer: I am a member of both) "did not include assessment of sex differences" or "only a few include sex-disaggregated data." These statements are overstating their case. NCD RisC generated Bayesian models separately by sex in all of their papers. HPACC always includes sex/gender as a variable in individual regression models.

We acknowledge the editor’s concern and appreciate the opportunity to clarify our point. Our intent is not to claim that global data groups do not disaggregate data by sex, but rather to highlight that in many cases, these data are limited to only some points along a health pathway, and that studies using these data do not adequately discuss sex differences. While we recognize that NCD RisC generates models separately by sex, our point is that the extent of sex-disaggregated analysis and discussion of sex differences remains limited in several key studies. To address the editor’s concerns, we have (1) revised the title of the paper to the following “Sex-Disaggregated Data Along the Gendered Health Pathways: A Review and Analysis of Global Data on Hypertension, Diabetes, HIV, and AIDS”; and (2) revised the 5th paragraph in the discussion as follows to more accurately reflect this distinction (see page 13, line 414).

Although global data groups such as NCD RisC include data disaggregated by sex, studies analyzing trends in risk factors, prevalence, and treatment for the three conditions we reviewed often provide limited discussion on sex differences or focus primarily on aggregate populations without explicitly assessing sex-based disparities. While several studies report estimates separately for females and males, they mostly do not include a detailed assessment of sex differences in outcomes (e.g., [6,32–34]). For example, a recent analysis of global trends in hypertension prevalence and treatment from 1990 to 2019 using data from the NCD Risk Factor Collaboration presented estimates stratified by sex but did not deeply examine sex differences in trends and outcomes [6,32]. Other studies, such as those by Rahim and colleagues [35] and Lee and colleagues [36], focus on total population estimates without sex-specific analyses. Additionally, studies have limitations in geographical coverage [33,36,37]. For example, three recent papers examined diabetes risk and preventive measures across low- and middle-income countries and the diagnostic testing for hypertension, diabetes, and hypercholesterolemia within similar economic groups [17,34,35], while another study explored trends in hypertension awareness, treatment, and control across 12 high-income countries [21]. Moreover, sex-disaggregated analyses, particularly regarding the care cascade for specific diseases, remain limited [11].

Another point is that in the discussion, the authors basically just go right into their talking points on how more disaggregated is needed, and the findings from the paper (which are legitimately interesting!) are basically buried. For example, in the abstract, the authors write, "Significant sex differences were found in each step along the pathways for many countries. Compared to females, males exhibited higher disease prevalence and death rates and lower rates of health care seeking, diagnosis and treatment adherence in many countries." Yet in the discussion, they write, "Discussion: "In our study, we found few countries with significant differences between males and females at any one of the points on the cascade for each of the three conditions." So which is it?

Thank you for this comment. It is important to distinguish between the overall health pathways and the care cascade. The health pathways include risk factors, disease prevalence, the care cascade, and death. The care cascade (proportion of diagnosed, treated, and controlled cases) is just one part of these pathways.

Our findings show that significant sex differences were observed at each step along the health pathways. Specifically, many countries (sometimes more than 100) reported significant sex differences in risk factors, disease prevalence, and death rates. However, when focusing solely on the care cascade, we found fewer countries (approximately 10 for hypertension, 1 for diabetes, and 20 for HIV/AIDS) with significant sex differences at any specific point in the cascade.

The sentence in the abstract refers to the entire health pathway:

"Significant sex differences were found in each step along the pathways for many countries. Compared to females, males exhibited higher disease prevalence and death rates and lower rates of health care seeking, diagnosis, and treatment adherence in many countries."

In contrast, the sentence in the discussion refers only to the care cascade and explores why fewer sex differences were found within it compared to other points in the health pathway:

"In our study, we found few countries with significant differences between males and females at any one of the points on the cascade for each of the three conditions."

To improve clarity, we have revised both sentences.

See abstract (page 2):

“Significant sex differences were found in each step along the pathways. In many countries, males exhibited higher disease prevalence and death rates than females, while in some countries, they also reported lower rates of health care seeking, diagnosis, and treatment adherence.”

See page 12, line 388:

“In our study, while many countries showed significant sex differences in risk factors, disease prevalence, and death, fewer exhibited significant differences between males and females within the care cascade for hypertension, diabetes, and HIV/AIDS. For example, for HIV and AIDS, while almost 60% of the countries show significant sex differences in drug use, unsafe sex, disease prevalence, and death rates, only 13% of the countries report significant sex differences in diagnosis, treatment and control."

Again, I suggest 2 key points:

1.	The authors in the first paragraph need to summarize the primary findings of the analysis. They can include an argument about the need for sex-specific data but it needs to be commensurate with the true findings from the paper.

2.	Even more critically, the authors need to meaningfully address the fact that their data mostly support that men are overburdened and underserved by health care systems. Perhaps this is not the finding they had expected to find, perhaps it is politically inconvenient, and perhaps it goes against some of their group's intended advocacy. But to me it absolutely jumps off the page. Yet, it is kind of buried. To me, the main finding of this paper is that men are an underserved population. This is not to say that women should be ignored, but this key finding that should be front and center and that the authors need to meaningfully discuss.

Thank you for this suggestion. We appreciate your insights and have revised the discussion section accordingly. We have restructured the discussion section to provide a clearer summary of the primary findings. The revised section first presents the observed sex differences, emphasizing that males experience a double burden—higher risk factors, disease prevalence, and mortality, while also being underserved by health care systems. This critical finding - which has been a finding from many previous studies, including those undertaken by co-authors on the paper - is now more prominently discussed. The second part of the discussion provides arguments on the need for sex-specific data, reinforcing the importance of addressing disparities in health care access and outcomes (see page 11, line 352).

The panels in Figure 2 are very difficult to interpret. They are inscrutable. I would suggest a key change. Have a single figure by disease and then have a spectrum shading that ranges from more countries male>female on one end to more countries female>male on the other end. Grey could be the neutral shade, rather than having 0% as a floor. I know the authors have an a priori argument about the need for sex-disaggregated cascades, but separating this figure does not help readers or further their argument. For example, look at the hypertension panels (Panels A and B) and the overweight column. It is yellow in both. What information does that convey? It means that sex differences are essentially balanced across the dataset, which is what the reader will intuitively be thinking. The figures could be pushed to the appendix or placed next to this new figure, which would thus illustrate both the between-country average and distribution.

Thank you for this suggestion. We have revised Figure 2, with single figures by disease and a spectrum shading that ranges from more countries Male > Female to more countries Female > Male. Figures of health pathways separated by sex have been moved to the Supporting Information and revised to make them consistent with the colour gradient of the new Figure 2 in the manuscript.

With respect to the regressions, I am fine with them leaving them out. I kind of agree that presenting the unadjusted estimates is more in line with the goal of the paper.

-----------------------------------------------------------

Comments from the reviewers:

Reviewer #2:

The authors have adequately addressed all of my comments

Reviewer #3 (statistics):

I thank the reviewers for comprehensively responding to my queries. My concerns have largely been addressed.

I have 3 outstanding comments:

1.	Statistical significance definition. I thank the reviewers for clarifying the implications of their methodology (i.e. difference being significant at a much lower level than the typical 5% that casual readers may have assumed). The implication that p-values for the differences are <=0.0025 is largely correct, but only if a few further assumptions (e.g. independence of the 2 estimators) are met.

Thank you for pointing this out. We have addressed it in the manuscript with the following clarification (see page 7, line 227).

By treating the male and female estimates as independent, […]

2.	Given the really stringent definition of significance, I think it is key in the results and discussion sections to not equate a lack of significance to a lack of a difference. For example the authors state that "Male smoking rates surpass those of females in 174 (87%) countries" but what they really mean is that smoking rates among men are significantly (according to the definition used in this manuscript) higher those among women in these countries. There may be other countries where these rates are higher, but not significantly so. I would suggest that the authors carefully review and revise the language used throughout the results and discussion sections.

We carefully reviewed and revised the language used throughout the results and discussion sections.

3.	The graphs are much easier to read now - thank you. The editors may want to check whether specific colours need to be changed for the benefit of colour-blind readers.

Thank you for this suggestion. We have revised Figure 2 accordingly.

-----------------------------------------------------------

General editorial requests:

(Note: not all will apply to your paper, but please check each item carefully)

1. We ask every co-author listed on the manuscript to fill in a contributing author statement, making sure to declare all competing interests. If any of the co-authors have not filled in the statement, we will remind them to do so when the paper is revised. If all statements are not completed in a timely fashion this could hold up the re-review process. If new competing interests are declared later in the revision process, this may also hold up the submission. Should there be a problem getting one of your co-authors to fill in a statement we will be in contact. Please do not add or remove authors without first discussing this with the handling editor. You can see our competing interests policy here:

The declaration of competing interests is included (see title page).

2. *Please upload any figures associated with your paper as individual TIF or EPS files with 300dpi resolution at resubmission; please read our figure guidelines for more information on our requirements. While revising your submission, please upload your figure files to the PACE digital diagnostic tool. PACE helps ensure that figures meet PLOS requirements. To use PACE, you must first register as a user. Then, login and navigate to the UPLOAD tab, where you will find detailed instructions on how to use the tool. If you encounter any issues or have any questions when using PACE, please email us at [PLOSMedicine@plos.org](mailto:PLOSMedicine@plos.org).

Figures uploaded individually.

3. Please ensure that the paper adheres to the PLOS Data Availability Policy which requires that all data underlying the study's findings be provided in a repository or as Supporting Information. For data residing with a third party, authors are required to provide instructions with contact information (web or email address) for obtaining the data. Please note that a study author cannot be the contact person for the data. PLOS journals do not allow statements supported by "data not shown" or "unpublished results." For such statements, authors must provide supporting data or cite public sources that include it.

We have added a data availability statement to ensure compliance with the PLOS Data Availability Policy (see title page).

Data Availability

Data on risk factors, disease prevalence, and death rates are available from the Global Burden of Disease Collaborative Network: Global Burden of Disease Study 2021 (GBD 2021), Institute for Health Metrics and Evaluation (IHME), Seattle, United States [<https://vizhub.healthdata.org/gbd-results/>]. Data on hypertension and diabetes care cascades can be accessed through NCD-RisC [https://ncdrisc.org/index.html], and HIV and AIDS care cascade data are available on the AIDSInfo website [<https://aidsinfo.unaids.org/>]. The code used in the analysis is available from Github [https://github.com/alessandroferaldi/Sex.Diff.Health.Pathways].

4. We expect all researchers with submissions to PLOS in which author-generated code underpins the findings in the manuscript to make all author-generated code available without restrictions upon publication of the work. In cases where code is central to the manuscript, we may require the code to be made available as a condition of publication. Authors are responsible for ensuring that the code is reusable and well documented. Please make any custom code available, either as part of your data deposition or as a supplementary file. Please add a sentence to your data availability statement regarding any code used in the study, e.g. "The code used in the analysis is available from Github [URL] and archived in Zenodo [DOI link]" Please review our guidelines and ensure that your code is shared in a way that follows best practice and facilitates reproducibility and reuse. Because Github depositions can be readily changed or deleted, we encourage you to make a permanent DOI'd copy (e.g. in Zenodo) and provide the URL.

The code used in the analysis is available from Github [https://github.com/alessandroferaldi/Sex.Diff.Health.Pathways].

FORMATTING – GENERAL

5. Title: Please revised the title to adhere to PLOS convention. The title should be non-declamatory and should include a colon followed by the study design at the end of the title.

Title revised (see title page).

Sex-Disaggregated Data Along the Gendered Health Pathways: A Review and Analysis of Global Data on Hypertension, Diabetes, HIV, and AIDS

6. Abstract: Please structure your abstract using the PLOS Medicine headings (Background, Methods and Findings, Conclusions). Please combine the Methods and Findings sections into one section.

Abstract headings revised and Methods and Findings sections combined into one section.

7. At this stage, we ask that you include a short, non-technical Author Summary of your research to make findings accessible to a wide audience that includes both scientists and non-scientists. The Author Summary should immediately follow the Abstract in your revised manuscript. This text is subject to editorial change and should be distinct from the scientific abstract. Ideally each sub-heading should contain 2-3 single sentence, concise bullet points containing the most salient points from your study. In the final bullet point of 'What Do These Findings Mean?', please include the main limitations of the study in non-technical language. Please see our author guidelines for more information:

Author Summary included (see page 3).

Why Was This Study Done?

- Despite strong evidence that sex and gender influence health outcomes, many health policies lack sex-specific approaches.
- Prior research on health trends presents results separated by sex, limiting discussions on sex differences and geographic coverage.

What Did the Researchers Do and Find?

- We reviewed global datasets and published papers on sex-disaggregated care cascades for eight major health conditions and analyzed sex differences along the health pathway.
- Findings revealed that males face a double burden of higher prevalence of risk factors and diseases while also experiencing lower access to diagnosis and treatment.
- The study shows the lack of sex-disaggregated care cascade data compared to risk exposure and health outcomes, creating gaps in understanding health inequities.

What Do These Findings Mean?

- The results highlight the need for strategies that encourage greater male participation in preventive and healthcare services.
- Findings underscore the importance of comprehensive sex-disaggregated data to better understand health inequities and inform gender-responsive interventions.
- More standardized and harmonized global datasets are needed to monitor sex differences and guide equitable healthcare policies.

8. Please express the main results with 95% CIs as well as p values. When reporting p values please report as p<0.001 and where higher as the exact p value p=0.002, for example. Throughout, suggest reporting statistical information as follows to improve clarity for the reader "22% (95% CI [13%,28%]; p</=)". Please be sure to define all numerical values at first use.

In our paper, we report proportions of countries, not effect estimates from statistical models. For example, 107 out of 204 countries (52%) show significant sex differences in hypertension mortality (higher in males). While we could calculate 95% binomial confidence intervals for this proportion (e.g., "53% (95% CI [45%, 60%]; p<0.05)"), applying this format may not be the most appropriate way to convey our findings. We also believe this approach may cause confusion by conflating the proportion of countries with individual significance levels. Sex-differences are significant at 0.0025 level, but confidence intervals for proportions of countries are 95% confidence intervals. Given this distinction, we deem our current reporting approach is both appropriate and clear for the reader.

9. Please include page numbers and line numbers in the manuscript file. Use continuous line numbers (do not restart the numbering on each page).

Page and line numbers.

10. Please cite the reference numbers in square brackets. Citations should precede punctuation.

Reference numbers cited in square brackets.

FIGURES AND TABLES

11. Please provide titles and legends for all figures and tables (including those in Supporting Information files).

Titles and legends provided for all figures and tables.

12. Please define all abbreviations used in each figure/table (including those in Supporting Information files).

Abbreviations defined.

13. Please consider avoiding the use of red and green in order to make your figure more accessible to those with color blindness.

Figures revised, avoiding the use of red and green.

SUPPLEMENTARY MATERIAL

14. Please note that supplementary material will be posted as supplied by the authors. Therefore, please amend it according to the relevant comments outlined here.

Supplementary Information is in line with the editorial requests.

15. Please cite your Supporting Information as outlined here:

Supporting Information cited.

REFERENCES

16. PLOS uses the numbered citation (citation-sequence) method and first six authors, et al.

Citations revised.

17. Please ensure that journal name abbreviations match those found in the National Center for Biotechnology Information (NCBI) databases and are appropriately formatted and capitalised.

Name abbreviations match those in NCBI.

18. Where website addresses are cited, please include the complete URL and specify the date of access (e.g. [accessed: 12/06/2023]).

Website addresses cited.

19. Please also see for further details on reference formatting.

Done.
